# Differential distribution and enrichment of non-coding RNAs in exosomes from normal and Cancer-associated fibroblasts in colorectal cancer

**DOI:** 10.1186/s12943-018-0863-4

**Published:** 2018-08-03

**Authors:** Mercedes Herrera, Carlos Llorens, Marta Rodríguez, Alberto Herrera, Ricardo Ramos, Beatriz Gil, Antonio Candia, María Jesús Larriba, Pilar Garre, Julie Earl, Mercedes Rodríguez-Garrote, Trinidad Caldés, Félix Bonilla, Alfredo Carrato, Vanesa García-Barberán, Cristina Peña

**Affiliations:** 1 Department of Medical Oncology, Hospital Universitario Puerta de Hierro de Majadahonda, Majadahonda, Madrid, Spain; 2 Department of Oncology-Pathology, Karolinska Institutet, Stockholm, Sweden; 3 Biotechvana, Scientific Park, University of Valencia, Valencia, Spain; 4 Department of Molecular Cell Biology, Institute for Cancer Research, University Hospital-The Norwegian Radium Hospital, and Centre for Cancer Biomedicine, Faculty of Medicine, University of Oslo, Oslo, Norway; 5 Pathology Department, Fundación Instituto de Investigación Jiménez Díaz, CIBERONC, Madrid, Spain; 6 Unidad de Genómica, Campus de Cantoblanco, Scientific Park of Madrid, Madrid, Spain; 7 Grupo de Investigación de oncología traslacional, Departamento de tumores digestivos, Hospital doce de Octubre, Madrid, Spain; 8 Pathology Department, Hospital Universitario de Guadalajara, Guadalajara, Spain; 9 Instituto de Investigaciones Biomédicas Alberto Sols, Consejo Superior de Investigaciones Científicas - Universidad Autónoma de Madrid, CIBERONC, Madrid, Spain; 10 Laboratorio de Oncología Molecular, Hospital Clínico San Carlos. Centro de Investigación Biomédica en Red de Cancer (CIBERONC), Madrid, Spain; 11 Medical Oncology Department, Ramón y Cajal University Hospital, IRYCIS, CIBERONC, Alcalá University, Madrid, Spain; 12 Centro de Estudios Biosanitarios, Madrid, Spain

**Keywords:** Colon Cancer, Liquid biopsy, Tumor microenvironment, Exosomes, Non-coding RNAs, Next generation sequencing

## Abstract

Exosome production from cancer-associated fibroblasts seems to be an important driver of tumor progression. We report the first in-depth biotype characterization of ncRNAs, analyzed by Next Generation Sequencing and Bioinformatics, expressed in established primary human normal and cancer-associated fibroblasts (CAFs) from cancer and normal mucosa tissues from 9 colorectal cancer patients, and/or packaged in their derived exosomes. Differential representation and enrichment analyses based on these ncRNAs revealed a significant number of differences between the ncRNA content of exosomes and the expression patterns of the normal and cancer-associated fibroblast cells. ncRNA regulatory elements are specifically packaged in CAF-derived exosomes, supporting a specific cross-talk between CAFs and colon cancer cells and/or other stromal cells, mediated by exosomes. These sncRNAs are potential biomarkers present in cancer-associated fibroblast-derived exosomes, which should thereby contribute to developing new non-invasive diagnostic, prognostic and predictive methods for clinical applications in management of cancer patients.

**Electronic supplementary material:**

The online version of this article (10.1186/s12943-018-0863-4) contains supplementary material, which is available to authorized users.

Tumor progression is deeply influenced by the local microenvironment. Fibroblasts usually named as cancer-associated fibroblasts (CAFs), are one of the most abundant and active cell types of the tumor microenvironment (TME). CAFs seem to regulate many aspects of tumorogenesis involving interactions between the malignant cells and other cells of the TME [1].

Exosomes, released by cells are important mediators of intercellular communication. In the tumor context, exosomes released by cancer cells transmit signals to cancer cells and also to stromal cells generating an active TME which promotes tumor progression [2]. Exosomes are also released by both cancer cells and stromal cells not only into the cancer microenvironment, but also into circulation [2]. Significant amounts of miRNAs packaged into exosomes were detected in many types of liquid biopsy samples [2], and certain types of circulating miRNAs strongly correlates with progression of different cancer types [3], however, few information about others ncRNAs contained in exosomes is unknown.

In the past decade large-scale analyses have focused on the comprehensive identification of non-coding RNAs (ncRNAs) and of new ones such us the long ncRNAs (lncRNAs). lncRNAs are poorly conserved and regulate gene expression by diverse mechanisms that are not yet fully understood. Recent data suggest that they may serve as master drivers of carcinogenesis in various types of tumors, such as breast, prostate and colon ones [4].

## Results and discussion

### Identification and quantification of ncRNA biotypes in cell and exosomal fractions from human primary normal and cancer-associated fibroblasts

Primary NFs and CAFs were propagated from 9 colorectal cancer patients (Additional file 1). RNA samples from CAF and NF derived exosomes were combined to get 3 pools of NF-derived exosomes consisting of 3 samples each (NF-EXO samples) vs 3 pools of CAF-derived exosomes consisting of 3 samples each (CAF-EXO samples). RNA samples from cells were combined in the same way (NF-CELL versus CAF-CELL samples).

**Fig. 1:**
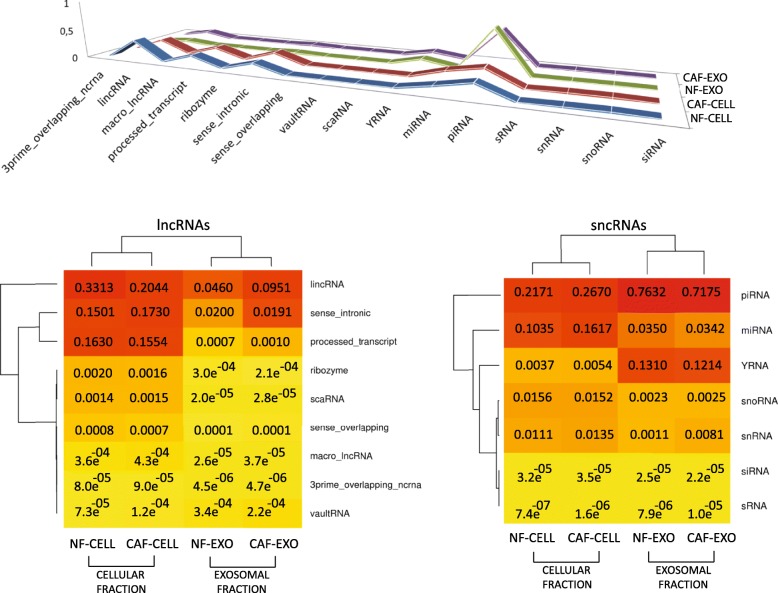
Above, 3D line chart, where the most prominent lines based on the average relative counts of reads mapped to each ncRNA biotype in NF-CELL, CAF-CELL, NF-EXO and CAF-EXO samples, are emphasized. Below, two heatmaps with double clustering based on the same information (to the left, heatmap based on lncRNA frequencies; to the right, the one for sncRNAs). The following ranges (0, 0.001), (0.0011, 0.05), (0.1, 0.9) were defined to color the breaks in yellow, orange and red, respectively. Dendrograms were inferred using the complete linkage with the Euclidean distance measure as clustering method

The expression patterns of 1668 lncRNA and 1761 sncRNA species related to 16 biotypes were analyzed by Next Generation Sequencing and differential expression analyses. The fractions of cell samples (NF-CELL and CAF-CELL) were more similar to each other than to the samples of the exosomal fraction (NF-EXO and CAF-EXO) and vice-versa (Fig. 1). Thus, biotypes such as sense intronic RNAs, lincRNAs and miRNAs appear to be higher in the cell fraction while YRNAs and piRNAs are more enriched in the exosomal fraction. For an absolute and average relative counts of reads mapped to each ncRNA biotype and samples details about the dispersion and biological variation, see Additional files 2, 3 and Additional file 4: Figure S1.

### Differential content and enrichment of ncRNAs in fibroblast-derived exosomes compared with their respective NFs or CAFs

**Fig. 2:**
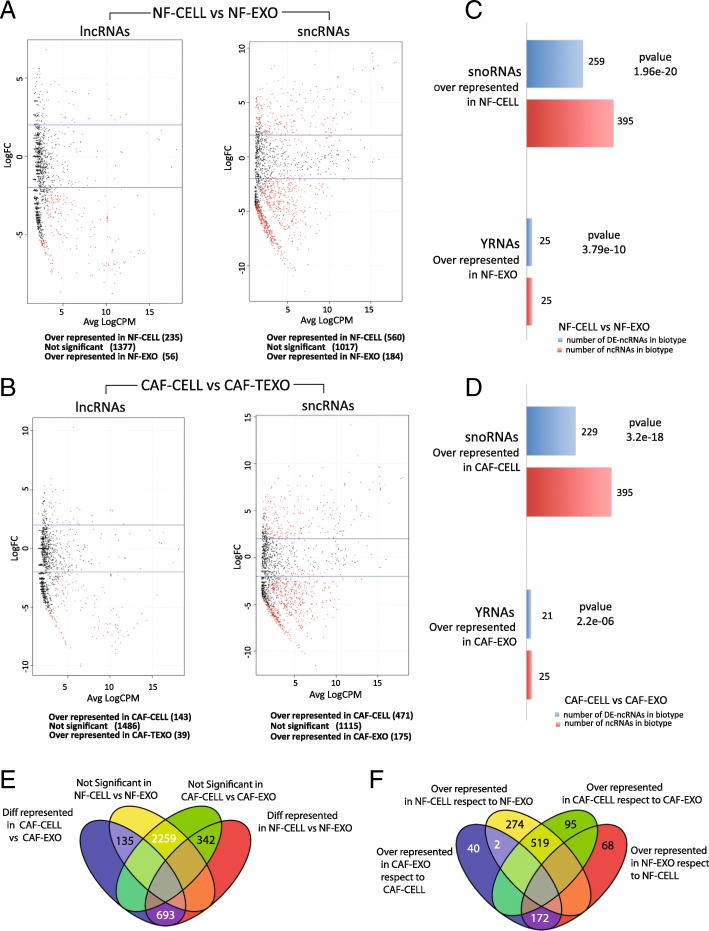
**a** Volcano plots from the differential expression analyses of lncRNAs and sncRNAs between NF-CELL versus NF-EXO samples. Red dots mean differentially distributed ncRNAs supported by FDR < 0.05. Below each plot we summarize the results of each analysis. **b** Volcano plots from the differential expression analyses of lncRNAs and sncRNAs between CAF-CELL and CAF-EXO samples. **c** Bar-plot showing the results of the differential enrichment analyses of biotypes performed between NF-CELL and NF-EXO samples accompanied by the *p* values for each biotype. **d** Results of the differential enrichment analysis of biotypes in CAF-CELL and CAF-EXO. **e** Venn diagram showing the relationships between significant and non-significant ncRNAs of both analyses, NF-CELL versus NF-EXO and CAF-CELL versus CAF-EXO. **f** Venn diagram showing the relationships among all over-represented ncRNAs of both comparisons

Differentially distributed ncRNAs between the exosomal and cellular contents in both NF and CAF data are shown in Fig. 2a and b and Additional files 5 and 6. Samples of the cellular fraction are significantly more enriched in “snoRNAs” than the exosomal fraction, which in turn resulted significantly enriched in YRNAs (Fig. 2c and d). The functional role/s of YRNAs is a current subject of debate. Specifically on cancer research, [5] recently reported enrichment of YRNAs in extracellular vesicles released from the human colon cancer LIM1863 cell line. Our results are consistent with that study, supporting the idea of a potential role for encapsulated YRNAs in exosomes.

According to Fig. 2e and f, 2 ncRNAs, respectively expressed by 2 related but different snRNA loci named U1 and RNVU1–17, are over-represented in the cell compartment when the analysis is based on NF cells and their exosomes, while they are over-represented in CAF-derived exosomes when the analysis is performed between CAF and their exosomes, which points to these 2 sncRNAs as potential biomarkers worth investigating. This suggests that the expression patterns and exosomal distribution of differentially expressed ncRNAs are apparently orchestrated by the normal or cancer-associated condition of fibroblast cells (See also Additional files 7 and 8).

### Deregulation and selective distribution of ncRNAs in CAFs and derived exosomes in comparison to NFs and their exosomes

**Fig. 3:**
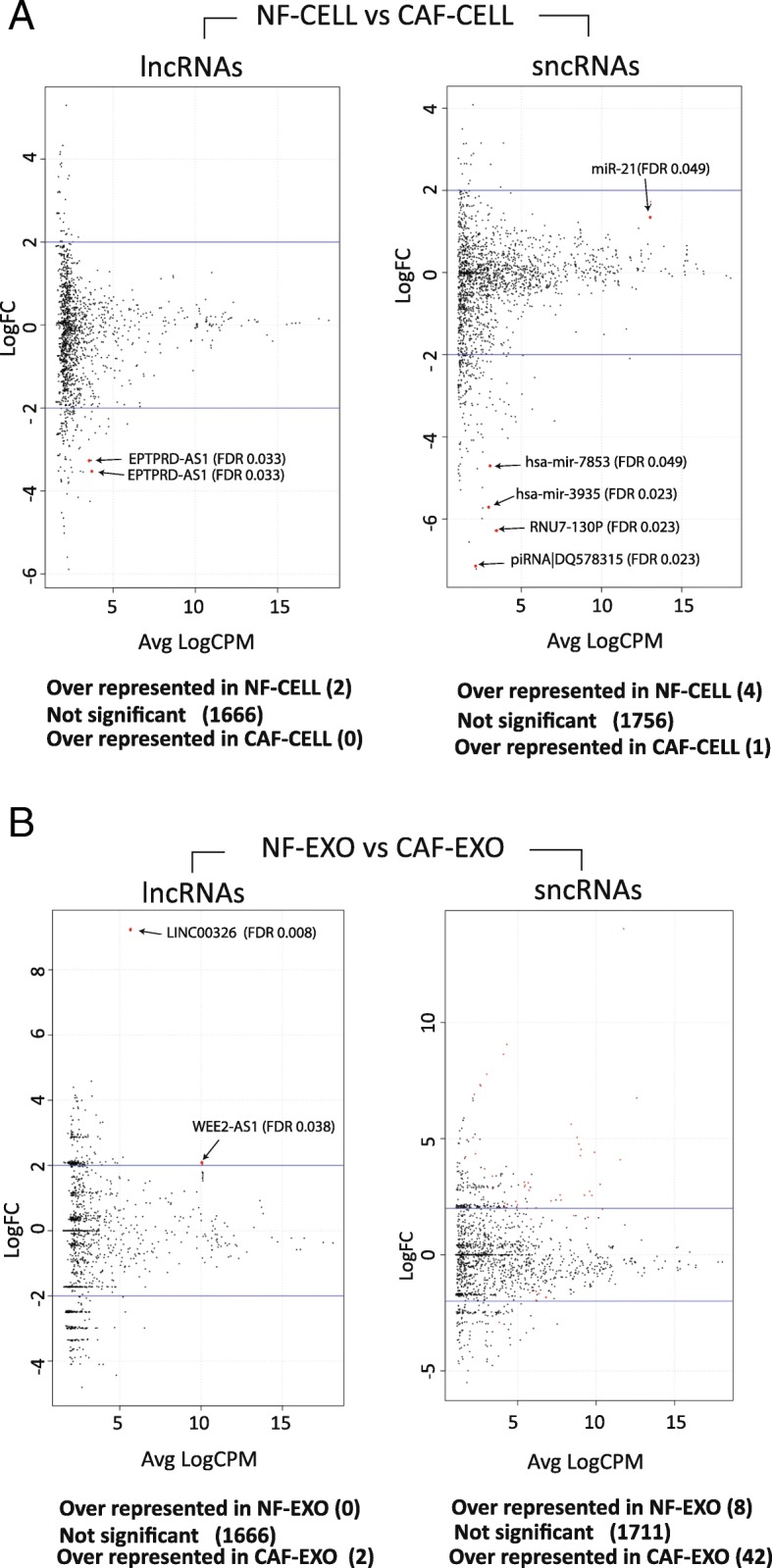
**a** Volcano plots from the differential expression analyses of lncRNAs and sncRNAs in NF-CELL and CAF-CELL samples. Red dots are significant ncRNAs supported by FDR < 0.05 and are 2× increased to let the reader see them clearly. Names for significant ncRNAs are also included. **b** Volcano plots from the differential expression analyses of lncRNAs and sncRNAs in NF-EXO and CAF-EXO samples created using similar criteria for the volcano plot previously shown in plot A. For more information about the 42 significant sncRNAs (red dots) see Additional file 11: Table S1

The analyses performed between NF-CELL versus CAF-CELL samples showed 2 lncRNAs under-expressed in CAF-CELL samples. Regarding sncRNAs, 2 miRNAs (MIR3935, MIR7853), 1 piRNA (piR-46,427) and 1 snRNA (RNU7-130P) were significantly under-expressed in CAF-CELL samples, while 1 miRNA (miR-21-related) was over-expressed (Fig. 3a and Additional file 9).

Fifty-two ncRNAs were differentially distributed between NF- and CAF-derived exosomes (Fig. 3b). 2 were lncRNAs (LINC00326 and WEE2-AS1), which were over represented in CAF-EXO samples (Additional file 10). The remaining 50 are summarized in Additional file 11: Table S1 and correspond to 8 sncRNAs that were under-represented and 42 sncRNAs over-represented in CAF-EXO samples (Additional file 12).

About the 42 sncRNAs over-represented in CAF-EXO, twelve of them correspond to sequences with no homologs, while the remaining 30 split into 4 families, designated as Families 1, 2, 3 and 4, each of which consisted of paralog sequences mapping in different loci (Additional file 13, Additional file 11: Table S1).

Among the over-represented sncRNAs of CAF-EXO samples, 26 snRNAs split into 2 and 23 paralogs, designated as Family 1 and 4, respectively, and a single sequence (RNU11) with no other homologs in our analyses. snRNAs are involved in splicing regulation, which are frequently altered in cancer [6]. Nevertheless, there is not much information about splicing alteration depending on snRNA regulation. Our data show over-distribution of many snRNAs in exosomes derived from CAFs. Remarkably, we also showed in this study that 2 of these sncRNAs, U1 and RNVU1–17, were more represented in CAF-derived exosomes than in their parental cells, but less than in NF-derived exosomes. This suggests that the tumor-associated condition plays a role in the expression patterns of these snRNAs.

We found up to 7 piRNAs over-represented in CAF-EXO samples. Three of them are paralogs constituting Family 2, while the remaining 4 are sequences with no homologs detected in our analyses. piRNAs interact with PIWI proteins and play a role in gene regulation and silencing by methylation or euchromatic histone modifications, which are mechanism clearly linked to the hallmarks of cancer [7], but they are also associated with exosomes and other microvesicles. The number of studies on piRNAs in cancer has increased during the last decade although their specific tumorigenic functions remain unclear [8].

Of the 9 miRNAs observed, 2 of them, mir6087 and its paralog AL353644.1, constitute Family 3. It is worth noting that miR-6087 has been related to colorectal cancer survival [9].

### Target genes for over-represented sncRNAs in CAF exosomes and associated gene ontologies

Prediction of target genes for CAF-EXO over-distributed sncRNAs (supported by FDR < 1E-04) is summarized in Additional file 14: Table S2. The piRNAs belonging to Family 2 were predicted to interact with 65 target genes, and similarly piR-57,251, which had 62 target genes. The Family 4 were predicted to interact with 15 target genes; and the RNU11 snRNA, with 7 target genes (remaining predictions are provided as Additional files 10 and 12).

The biological process identified by GO and metabolic pathway annotations (Additional file 15: Figure. S2) represents functions or processes of immediate relevance to tumor progression and microenvironment. Presumably, the effect of CAF-derived exosomes could affect not only tumor cells but also other cells from the tumor microenvironment, prompting tumor progression.

In summary, the ncRNA species act in the regulation of diverse cell functions [10]. Our data support a regulatory role of the deregulated ncRNAs in CAFs, possibly involved with CAFs’ activation process or specifically with the cross-talk between stromal and cancer cells. The levels of some of these regulatory elements are differentially represented in CAF-derived exosomes pointing to the clinical relevance of our study, since the identification of these markers in exosomes could be translated into two kinds of new clinical tools. Firstly, these ncRNA could be potentially used as new biomarkers for patient prognoses and prediction of response to therapy in cancer. But secondarily and more important, cancer exosomes are delivered to circulating body fluids, which gives an opportunity to develop new tools for oncology patients management by minimally invasive blood analyses. Moreover, the low cost together with the feasibility of a non-invasive method offer the chance to define intervals during treatment and the course of the disease to monitor cancer progression and patients’ evolution.

## Additional files

Additional file 1: Supplementary material and methods. (DOCX 28 kb)

Additional file 2: Absolute and average relative counts of reads mapped to each ncRNAs biotype per sample and fraction. (XLSX 15 kb)

Additional file 3: Excel document with the two count files used as input to EdgeR for differential expression analysis; one with the counts of reads of all samples mapped on the lncRNA references and another with the read counts of reads mapped on scnRNAs. (XLSX 221 kb)

Additional file 4: **Figure S1.** logFC-based MDS-plots (one for each type of lncRNA), where the first dimension corresponds to differences due to the type of sample (i.e. whether it is a normal cell, a tumor cell or an exosomal sample) and the second dimension corresponds to the differences between the samples themselves as biological replicates. The inferred dispersion and the Biological Coefficient of Variation (BCV) of all assayed samples for lncRNAs are 0.21201 and 0.4604, respectively, while the coefficients for sncRNA content are 0.25164 and 0.5016. These two coefficients reveal some interesting variation among samples. In this respect the multidimensional scaling (MDS) plots where the differences in ncRNA content between cellular and exosomal fractions and the heterogeneity of CAF samples, and especially their exosomes, are indicated as the main cause of this variation. Moreover, since each sequenced sample was prepared as a pool of three others and CAF samples and their exosomes are, as expected, more heterogenous because of their tumoral condition, then their variability should also be greater than that of NFs and their exosomes. **Figure S1** also gives some differences that exist between NF and CAF exosomes. (PNG 104 kb)

Additional file 5: Excel file with two documents summarizing the results obtained from the differential expression NF-CELL versus NF-EXO analyses for differentially distributed lncRNAs and sncRNAs. (XLSX 73 kb)

Additional file 6: Excel file with two documents summarizing the results obtained from the differential expression CAF-CELL versus CAF-EXO analyses for differentially distributed lncRNAs and sncRNAs. (XLSX 60 kb)

Additional file 7: Mini web site presenting a dynamic venn diagram intersecting the relationships of significance from the assayed ncRNAs in the differential expression analyses performed between NF- and CAF- exosomes versus their respective cellular environments (i.e. NF-CELL versus NF-EXO and CAF-CELL versus CAF-EXO). Clicking on any intersected number, the web site opens a dialog summarizing the ncRNAs species that correspond to the intersection. (ZIP 362 kb)

Additional file 8: Mini web site presenting a dynamic venn diagram showing the relationships between the results cellular or exosomal over represented in the two analyses performed between NF- and CAF- exosomes. Clicking on any intersected number, the web site opens a dialog summarizing the ncRNAs species that correspond to the intersection. (ZIP 354 kb)

Additional file 9: Excel file with two documents summarizing the results obtained from the differential expression NF-CELL versus CAF-CELL analyses for differentially expressed lncRNAs and sncRNAs. (XLSX 10 kb)

Additional file 10: Excel document with two documents summarizing the results for the significant lncRNAs in the differential expression analysis between NF-EXO and CAF-EXO samples and target predictions for those significant in CAF-EXO samples (positive logFCs). (XLS 40 kb)

Additional file 11: **Table S1**. sncRNAs distributed differently in CAF-EXO samples from in NF-EXO ones. Highly significant lncRNAs and sncRNAs (FDR < 1E-04) are highlighted in bold. (DOCX 79 kb)

Additional file 12: Excel document with two documents summarizing the results for the significant sncRNAs in the differential expression analysis between NF-EXO and CA-FEXO samples and target predictions for those significant in CAF-EXO samples (positive logFCs). (XLSX 292 kb)

Additional file 13 : Multiple alignment of the 42 sncRNAs over represented in CAF-EXO samples. (FASTA 8 kb)

Additional file 14 : **Table S2**. Target genes for CAF-EXO over-distributed sncRNAs supported by FDR < 1E-04. (DOCX 14 kb)

Additional file 15 : **Figure S2**. Heatmap with dendrogram showing the over-represented sncRNAs in CAF exosomes supported by FDRs below 1E-04 with the GO terms and metabolic pathways annotated to the predicted target genes summarized in Table 2. The number of target genes is used to color the breaks. If a sncRNA has no target gene assigned to a metabolic pathway (absence), the intersecting cell is colored white; if a ncRNA has one target gene assigned to a pathway, the cell is colored gray; and if more than two target genes are assigned to a pathway, it is colored black. The clustering was inferred by using the complete linkage with the Euclidean distance measure. (PNG 578 kb)

## Abbreviations

CAF: Cancer associated fibroblast
ECM: Extracellular matrix
lncRNA: Long non-coding RNA
logFC: Log Fold Change
MDS: Multidimensional Scalin
miRNA: miRNA
ncRNA: Non-Coding RNA
NF: Normal fibroblast
piRNA: PIWI Interacting RNA
RNA-Seq: RNA Sequencing
sncRNA: Short non-coding RNA
snoRNA: Small nucleolar RNA
snRNA: Small nuclear RNA
TME: Tumor microenvironment
