# Supplementary material for: Differential distribution and enrichment of non-coding RNAs in exosomes from normal and Cancer-associated fibroblasts in colorectal cancer
Source: Mol Cancer. 2018 Aug 3;17:114. doi: 10.1186/s12943-018-0863-4 (PMC6091058; doi:10.1186/s12943-018-0863-4)
Supplement: Supplementary file 1 — Supplementary material and methods. (DOCX 28 kb) [file 12943_2018_863_MOESM1_ESM.docx]

**SUPPLEMENTARY MATERIAL**

**MATERIAL AND METHODS**

**Human samples**

Fresh tissue from 9 patients operated for colorectal primary tumors at the Puerta de Hierro-Majadahonda University Hospital was used for the propagation of primary CAFs and NFs. Informed written consent was obtained from all participants after an explanation of the nature of the study, as approved by the Research Ethics Board of Puerta de Hierro Majadahonda University Hospital.

**Establishment of cancer-associated and normal fibroblasts in primary colon cancer**

All the fibroblast strains were derived in the same way from explant cultures following procedures described elsewhere (1).

**Isolation, characterization and quantification of exosomes**

CAF and NF human primary cultures were cultured for 48h in DMEM supplemented by 1% exosome-depleted FBS. The latter was depleted of bovine exosomes by passage through a 0.22-µm PVDF filter (Millipore) and ultracentrifugation at 120,000 *g* for 90 minutes. Supernatant fractions collected were pelleted by centrifugation at 500 *g* for 10 minutes at 4ºC to eliminate cells. Exosomes were isolated from culture medium with ExoQuick™ TC Kit (System Bioscience) in line with the manufacturer’s instructions.

**RNA isolation**

Total RNA was extracted from cells and exosomes by the *mir*Vana^TM^ miRNA Isolation Kit (Ambion Inc.). The quality of total RNA, including small RNA, extracted from cell and exosome fractions of CAFs and NFs was analyzed by an Agilent 2100 Bioanalyzer (http://www.chem.agilent.com) equipped with an RNA 6000 pico LabChip kit, following the manufacturer’s recommendations.

**Library preparation and Illumina sequencing**

Libraries were prepared according to the instructions of the “NEBNext Multiplex Small RNA Library Prep Set for Illumina” kit from New England Biolabs. The input yield of total RNA to start the library protocol was 1 µg of each pool quantified by bioanalyzer using RNA 6000 Nano Chips (333ng of each sample/pool when pooling 3 samples). Before starting the protocol, we concentrated the RNA pools to 6 µl using a SpeedVac instrument. The library amplification step included a PCR step of 13 cycles.

The libraries obtained were validated and quantified by an Agilent 2100 Bioanalyzer using High-Sensitivity DNA Chips. An equimolecular pool of libraries of each type (exosomes and cells) was purified by acrylamide gel electrophoresis selecting the size region from 140pb to 400pb. The obtained size-selected pools of libraries were titrated by quantitative PCR using the “Kapa-SYBR FAST qPCR kit forLightCycler480” (Kapa BioSystems) and a reference standard for quantification. These two acrylamide-purified pools of libraries were denatured prior to being seeded on a flow-cell at a density of 2.2pM. The libraries were sequenced using a “NextSeq™ 500 High Output Kit”. A 1x75 single-end sequencing run on a NextSeq500 sequencer was performed (in an attempt to manage an intermediate-read size sequence allowing us to capture both the lncRNAs and the sncRNAs of each sample in the same sequencing run).

**Differential expression and enrichment analyses**

Quality Analysis and preprocessing: Quality control on Fastq libraries used the FastQC tool. Subsequently, Fastq files were preprocessed by Cutadapt (2) and Prinseq (3) to eliminate primers and low-quality sequences.

RefSeq ncRNA databases: We created a sequence database of 15,702 lncRNAs belonging to 9 different ncRNA biotypes. The database contains 15,685 ncRNAs longer than 300 nucleotides annotated in the human genome GRCh38 available in the Ensembl Release 84 (4), which includes 3prime overlapping RNAs, lincRNAs, macro lncRNAs, processed transcript, ribozymes, both sense-intronic and sense-overlapping RNAs, vaultRNAs and scaRNAs. This database was completed with 17 representative sequences of non-human lincRNAs obtained from lncRNAdb (5). Here it is worth noting that scaRNAs are currently not considered long ncRNAs, but more properly small ncRNAs classified as snoRNAs. However, we decided to include scaRNAs in the database of lncRNAs for the sake of the bioinformatic protocol, as scaRNAs are greater than 300 nucleotides. In parallel, we created a sequence database with 11,335 sncRNAs belonging to 7 different biotypes. This includes annotations of YRNAs, miRNAs, sRNA, snRNA and snoRNAs retrieved from the GRCh38 release and piwi-interacting RNAs (piRNAs) and small-interfering RNAs (siRNAs) downloaded from the piRNABank (6) and the siRNADB (sirna.sbc.su.se/).

Splitting lncRNAs and sncRNAs: BLAT (7) was used to map preprocessed Fastq libraries against the refseq database of lncRNAs created in the former step. Then two scripts were created ad hoc to identify reads overlapping on refseq lncRNA fragments of more than 300 nucleotides, and then to extract them into a new collection of Fastq files specifically containing these lncRNA-related reads (authors can provide these two scripts on request). The remaining reads were considered sncRNA candidates and then stored in another collection of sncRNA-specific Fastq libraries. It is plausible to think that under this strategy we may lose information of those sncRNAs mapping within the sequence of lncRNAs, but in these cases what we assume and count is the lncRNA container as a unit of greater size.

Differential expression analyses between cell and exosomal fractions used the RNAseq pipeline of the GPRO suite (8). LncRNAs and sncRNAs were assayed in independent analyses to avoid size bias using the following workflow. First) Raw reads were mapped to the refseq database of lncRNAs using Bowtie2 (9). Second) Corset (10) created a file with clusters of ncRNA species and a count file integrating the raw counts of reads mapped to each cluster for each assayed sample. There were no clusters of more than one species, so we assumed each cluster to be representative of a specific ncRNA species. Third) EdgeR (11) was used to perform the differential expression analyses with the trimmed means of M values as method of normalization and each sample considered as a biological replicate. It was also used to infer the dispersion and Biological Coefficient of Variation (BCV) among samples.

Differential enrichment analyses performed used GOseq (12). Enrichment categories supported by Pvalues < 0.05 in the resulting Wallenious distribution from GOseq analyses were considered significant. As performed in differential expression analyses, lncRNAs and sncRNAs were assayed via separate analyses. It is also worth stressing that, although the standard usage of GOseq is oriented to testing the enrichment of GO categories by taking into account the size bias from RNAseq data, the statistical principle and algorithms behind the tool are exactly the same (and therefore perfectly valid) as those required when investigating the enrichment of any other categorizable feature in RNAseq data when selection bias is taken into account.

**Non-coding RNA gene names**

NcRNAs gene names were collected from the Ensembl BIOMART platform (34) and the lncRNAdb (5).

**Predictions of gene targets and related gene ontologies and metabolic pathways**

We created a sequence database with 3,072 transcript sequences from 361 human genes associated in some way with colorectal cancer according to information retrieved from the Online Mendelian Inheritance in Man (OMIM) database (13). The sequences were downloaded from the GRCh38 set of refseq sequences available in the Ensembl Release 84. This database was used as a reference to predict RNA-RNA interactions for lncRNAs and sncRNAs whose significance in differential expression analyses was supported by FDRs < 1E-04, using the software LncTar (14) setting normalized binding free energy (ndG) < -0.20 for lncRNAs and < -2.0 for sncRNAs as threshold parameters. Information of the Gene Ontologies (GO) and the KEGG metabolic pathways associated with the colorectal genes predicted as targets was retrieved from the Colorectal Cancer Atlas (15).

**REFERENCES**

1. Herrera M, Herrera A, Larriba MJ, Ferrer-Mayorga G, García de Herreros A, Bonilla F, et al. Colon Cancer-associated Fibroblast Establishment and Culture Growth. BIO-PROTOCOL 2016;6(7). doi:10.21769/BioProtoc.1773.

2. Martin M. Cutadapt removes adapter sequences from high-throughput sequencing reads. EMBnet.journal 2011;17(1):10.

3. Schmieder R, Edwards R. Fast identification and removal of sequence contamination from genomic and metagenomic datasets. PLoS One 2011;6(3):e17288.

4. Cunningham F, Amode MR, Barrell D, Beal K, Billis K, Brent S, et al. Ensembl 2015. Nucleic Acids Res. 2015;43(D1):D662–D669.

5. Quek XC, Thomson DW, Maag JL, Bartonicek N, Signal B, Clark MB, et al. lncRNAdb v2.0: expanding the reference database for functional long noncoding RNAs. Nucleic Acids Res. 2015;43(D1):D168–D173.

6. Sai Lakshmi S, Agrawal S. piRNABank: a web resource on classified and clustered Piwi-interacting RNAs. Nucleic Acids Res. 2008;36(suppl_1):D173–D177.

7. Kent WJ. BLAT--the BLAST-like alignment tool. Genome Res. 2002;12(4):656–64.

8. Futami R, Muñoz-Pomer A, Viu JM, Dominguez-Escribá L, Covelli L, Bernet GP, et al. GPRO: the professional tool for management, functional analysis and annotation of omic sequences and databases. Biotechvana Bioinforma. 2011-SOFT3. http://www.biotechvana.com. cited November 15, 2017

9. Langmead B, Trapnell C, Pop M, Salzberg SL. Ultrafast and memory-efficient alignment of short DNA sequences to the human genome. Genome Biol. 2009;10(3):R25.

10. Davidson NM, Oshlack A. Corset: enabling differential gene expression analysis for de novo assembled transcriptomes. Genome Biol. 2014;15(7):410.

11. Robinson MD, McCarthy DJ, Smyth GK. edgeR: a Bioconductor package for differential expression analysis of digital gene expression data. Bioinformatics 2010;26(1):139–40.

12. Young MD, Wakefield MJ, Smyth GK, Oshlack A. Gene ontology analysis for RNA-seq: accounting for selection bias. [Internet]. Genome Biol. 2010;11(2):R14.

13. Sayers EW et al. Database resources of the National Center for Biotechnology Information. Nucleic Acids Res. 2009;37(Database issue):D5-15.

14. Li J et al. LncTar: a tool for predicting the RNA targets of long noncoding RNAs. Brief. Bioinform. 2015;16(5):806–12.

15. Chisanga D et al. Colorectal cancer atlas: An integrative resource for genomic and proteomic annotations from colorectal cancer cell lines and tissues. Nucleic Acids Res. 2016;44(D1):D969-74.
