# Supplementary figures and images for: Differential distribution and enrichment of non-coding RNAs in exosomes from normal and Cancer-associated fibroblasts in colorectal cancer
Source: Mol Cancer. 2018 Aug 3;17:114. doi: 10.1186/s12943-018-0863-4 (PMC6091058; doi:10.1186/s12943-018-0863-4)

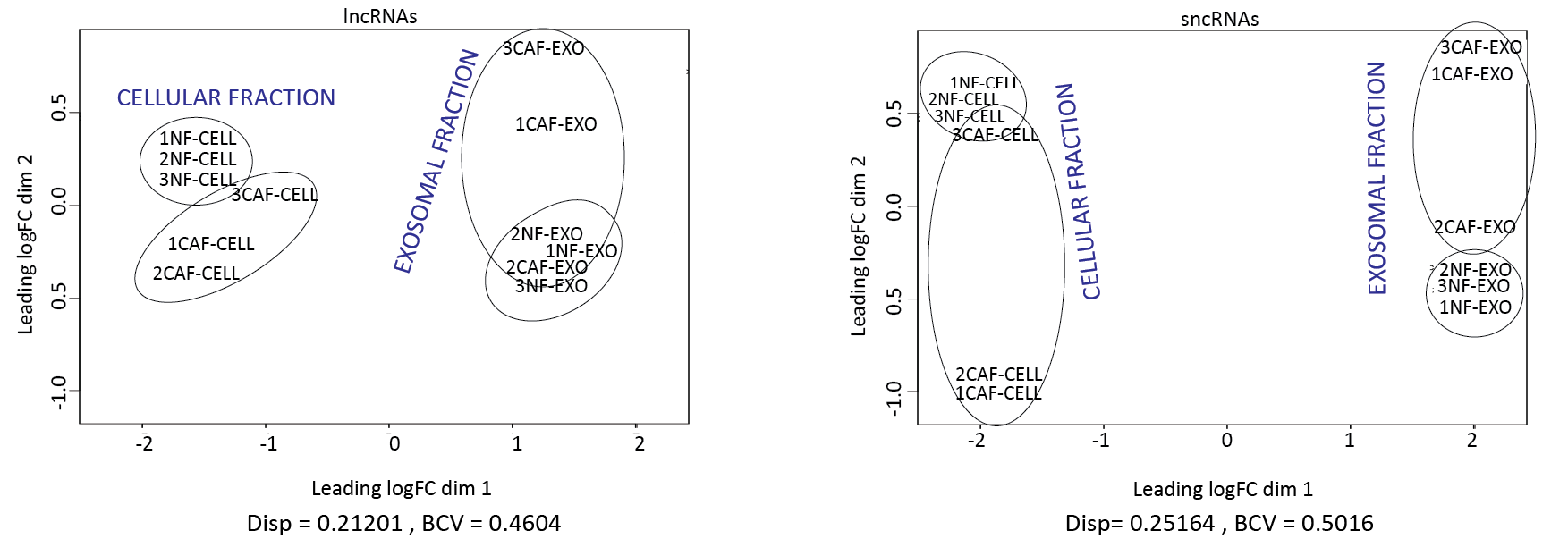

Supplement: Supplementary file 4 — Figure S1. logFC-based MDS-plots (one for each type of lncRNA), where the first dimension corresponds to differences due to the type of sample (i.e. whether it is a normal cell, a tumor cell or an exosomal sample) and the second dimension corresponds to the differences between the samples themselves as biological replicates. The inferred dispersion and the Biological Coefficient of Variation (BCV) of all assayed samples for lncRNAs are 0.21201 and 0.4604, respectively, while the coefficients for sncRNA content are 0.25164 and 0.5016. These two coefficients reveal some interesting variation among samples. In this respect the multidimensional scaling (MDS) plots where the differences in ncRNA content between cellular and exosomal fractions and the heterogeneity of CAF samples, and especially their exosomes, are indicated as the main cause of this variation. Moreover, since each sequenced sample was prepared as a pool of three others and CAF samples and their exosomes are, as expected, more heterogenous because of their tumoral condition, then their variability should also be greater than that of NFs and their exosomes. Figure S1 also gives some differences that exist between NF and CAF exosomes. (PNG 104 kb) [file 12943_2018_863_MOESM4_ESM.png]

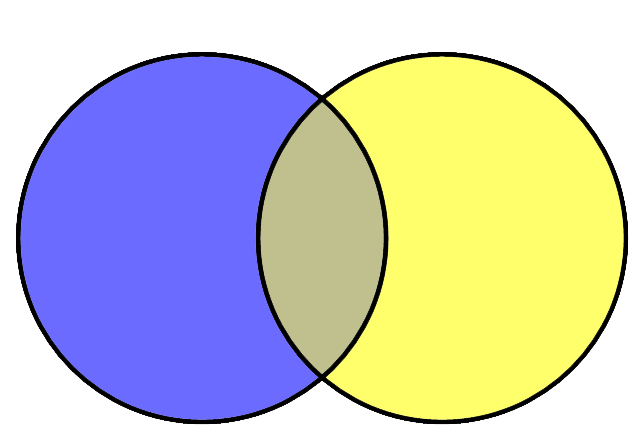

Supplement: Supplementary file 7 — Mini web site presenting a dynamic venn diagram intersecting the relationships of significance from the assayed ncRNAs in the differential expression analyses performed between NF- and CAF- exosomes versus their respective cellular environments (i.e. NF-CELL versus NF-EXO and CAF-CELL versus CAF-EXO). Clicking on any intersected number, the web site opens a dialog summarizing the ncRNAs species that correspond to the intersection. (ZIP 362 kb) [file 12943_2018_863_MOESM7_ESM.zip › Additional File 7/data/venn2.png]

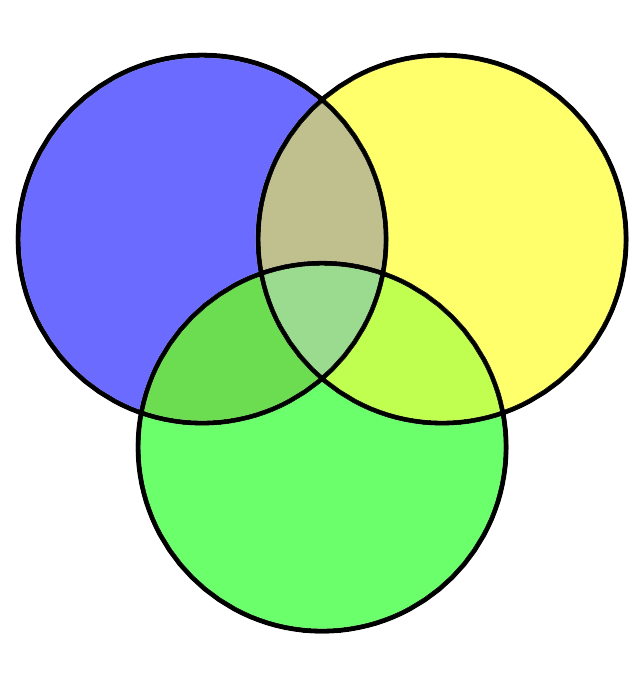

Supplement: Supplementary file 7 — Mini web site presenting a dynamic venn diagram intersecting the relationships of significance from the assayed ncRNAs in the differential expression analyses performed between NF- and CAF- exosomes versus their respective cellular environments (i.e. NF-CELL versus NF-EXO and CAF-CELL versus CAF-EXO). Clicking on any intersected number, the web site opens a dialog summarizing the ncRNAs species that correspond to the intersection. (ZIP 362 kb) [file 12943_2018_863_MOESM7_ESM.zip › Additional File 7/data/venn3.png]

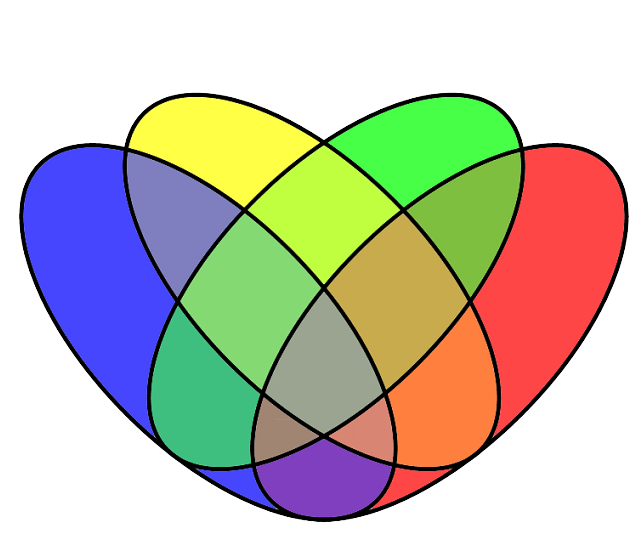

Supplement: Supplementary file 7 — Mini web site presenting a dynamic venn diagram intersecting the relationships of significance from the assayed ncRNAs in the differential expression analyses performed between NF- and CAF- exosomes versus their respective cellular environments (i.e. NF-CELL versus NF-EXO and CAF-CELL versus CAF-EXO). Clicking on any intersected number, the web site opens a dialog summarizing the ncRNAs species that correspond to the intersection. (ZIP 362 kb) [file 12943_2018_863_MOESM7_ESM.zip › Additional File 7/data/venn4.png]

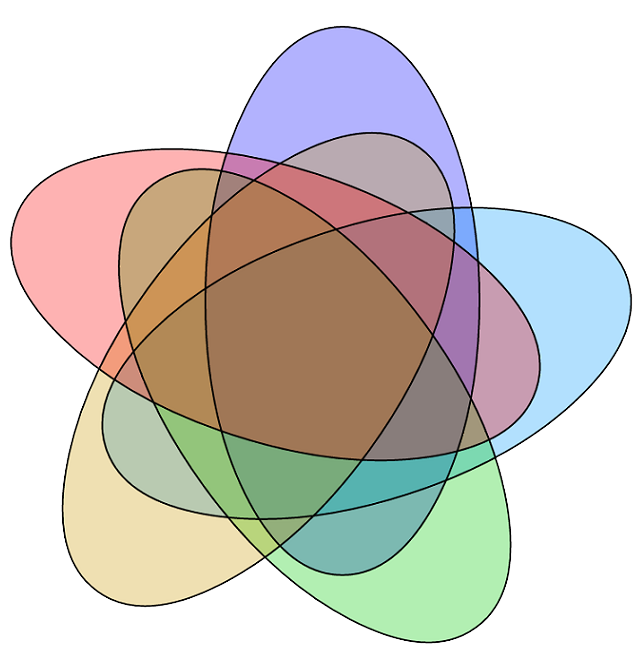

Supplement: Supplementary file 7 — Mini web site presenting a dynamic venn diagram intersecting the relationships of significance from the assayed ncRNAs in the differential expression analyses performed between NF- and CAF- exosomes versus their respective cellular environments (i.e. NF-CELL versus NF-EXO and CAF-CELL versus CAF-EXO). Clicking on any intersected number, the web site opens a dialog summarizing the ncRNAs species that correspond to the intersection. (ZIP 362 kb) [file 12943_2018_863_MOESM7_ESM.zip › Additional File 7/data/venn5.png]

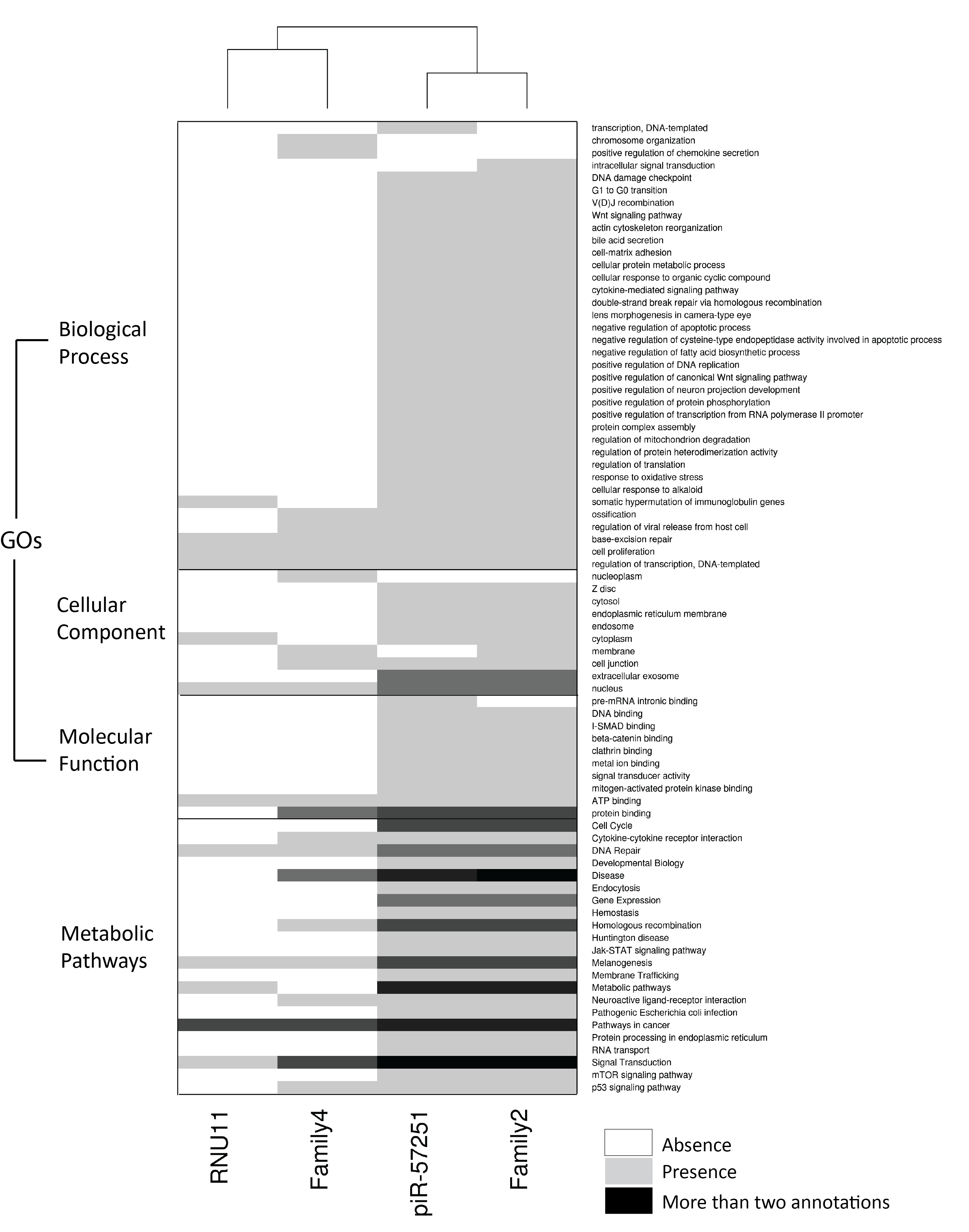

Supplement: Supplementary file 15 — : Figure S2. Heatmap with dendrogram showing the over-represented sncRNAs in CAF exosomes supported by FDRs below 1E-04 with the GO terms and metabolic pathways annotated to the predicted target genes summarized in Table 2. The number of target genes is used to color the breaks. If a sncRNA has no target gene assigned to a metabolic pathway (absence), the intersecting cell is colored white; if a ncRNA has one target gene assigned to a pathway, the cell is colored gray; and if more than two target genes are assigned to a pathway, it is colored black. The clustering was inferred by using the complete linkage with the Euclidean distance measure. (PNG 578 kb) [file 12943_2018_863_MOESM15_ESM.png]
