# Supplementary material for: Differential distribution and enrichment of non-coding RNAs in exosomes from normal and Cancer-associated fibroblasts in colorectal cancer
Source: Mol Cancer. 2018 Aug 3;17:114. doi: 10.1186/s12943-018-0863-4 (PMC6091058; doi:10.1186/s12943-018-0863-4)
Supplement: Supplementary file 7 — Mini web site presenting a dynamic venn diagram intersecting the relationships of significance from the assayed ncRNAs in the differential expression analyses performed between NF- and CAF- exosomes versus their respective cellular environments (i.e. NF-CELL versus NF-EXO and CAF-CELL versus CAF-EXO). Clicking on any intersected number, the web site opens a dialog summarizing the ncRNAs species that correspond to the intersection. (ZIP 362 kb) [file 12943_2018_863_MOESM7_ESM.zip › Additional File 7/venn.html]

## Venn diagram

This script creates venn diagrams of up to 5 samples using up to 5 csv files as arguments. This is, to create a 2-samples venn diagram you
must to upload 2 csvs, to a create a 4-samples venn diagram upload 4 csvs and so on. Each csv must contain a list with the items to compare
among samples.

### Requirements

Here you have an example of zip input accepted by the script.

### Parameters

Input column separator
; (semicolon)
"\t" (tab)
, (comma)
" " (space)

### Run the analysis

Upload the input csv and click on RUN button the script will return you a zip file containing the results.
  
  

  
  

  
  

  
  

  
  

  
  

  
  

Download


135

2259

693

342

**Elements in Diff Represented in CAF-CELL vs CAF-EXO**

**Elements in Not Significant in NF-CELL vs NF-EXO**

**Elements in Not Significant in CAF-CELL vs CAF-EXO**

**Elements in Diff Represented in NF-CELL vs NF-EXO**

**Elements in Diff Represented in CAF-CELL vs CAF-EXO and Not Significant in NF-CELL vs NF-EXO**
  


---

Grc38\_ENST00000362104|miRNA

---

Grc38\_ENST00000362105|miRNA

---

Grc38\_ENST00000362116|miRNA

---

Grc38\_ENST00000362125|miRNA

---

Grc38\_ENST00000362134|miRNA

---

Grc38\_ENST00000362147|miRNA

---

Grc38\_ENST00000362162|miRNA

---

Grc38\_ENST00000362173|miRNA

---

Grc38\_ENST00000362195|miRNA

---

Grc38\_ENST00000362215|miRNA

---

Grc38\_ENST00000362218|miRNA

---

Grc38\_ENST00000362227|miRNA

---

Grc38\_ENST00000362263|miRNA

---

Grc38\_ENST00000362265|miRNA

---

Grc38\_ENST00000362283|miRNA

---

Grc38\_ENST00000362295|miRNA

---

Grc38\_ENST00000362298|miRNA

---

Grc38\_ENST00000362317|miRNA

---

Grc38\_ENST00000364699|snoRNA

---

Grc38\_ENST00000364995|snoRNA

---

Grc38\_ENST00000365731|miRNA

---

Grc38\_ENST00000384121|snRNA

---

Grc38\_ENST00000384335|snoRNA

---

Grc38\_ENST00000384384|snoRNA

---

Grc38\_ENST00000384679|snoRNA

---

Grc38\_ENST00000384853|miRNA

---

Grc38\_ENST00000384863|miRNA

---

Grc38\_ENST00000384871|miRNA

---

Grc38\_ENST00000384879|miRNA

---

Grc38\_ENST00000384881|miRNA

---

Grc38\_ENST00000384889|miRNA

---

Grc38\_ENST00000384898|miRNA

---

Grc38\_ENST00000384901|miRNA

---

Grc38\_ENST00000384906|miRNA

---

Grc38\_ENST00000384915|miRNA

---

Grc38\_ENST00000384918|miRNA

---

Grc38\_ENST00000384965|miRNA

---

Grc38\_ENST00000384970|miRNA

---

Grc38\_ENST00000384992|miRNA

---

Grc38\_ENST00000385004|miRNA

---

Grc38\_ENST00000385009|miRNA

---

Grc38\_ENST00000385011|miRNA

---

Grc38\_ENST00000385015|miRNA

---

Grc38\_ENST00000385025|miRNA

---

Grc38\_ENST00000385026|miRNA

---

Grc38\_ENST00000385043|miRNA

---

Grc38\_ENST00000385050|miRNA

---

Grc38\_ENST00000385055|miRNA

---

Grc38\_ENST00000385060|miRNA

---

Grc38\_ENST00000385077|miRNA

---

Grc38\_ENST00000385199|miRNA

---

Grc38\_ENST00000385209|miRNA

---

Grc38\_ENST00000385223|miRNA

---

Grc38\_ENST00000385230|miRNA

---

Grc38\_ENST00000385231|miRNA

---

Grc38\_ENST00000385240|miRNA

---

Grc38\_ENST00000385258|miRNA

---

Grc38\_ENST00000385299|miRNA

---

Grc38\_ENST00000390180|miRNA

---

Grc38\_ENST00000401182|miRNA

---

Grc38\_ENST00000408492|miRNA

---

Grc38\_ENST00000408687|miRNA

---

Grc38\_ENST00000411509|lincRNA

---

Grc38\_ENST00000419813|lincRNA

---

Grc38\_ENST00000454224|lincRNA

---

Grc38\_ENST00000458220|processed\_transcript

---

Grc38\_ENST00000459126|snoRNA

---

Grc38\_ENST00000515031|lincRNA

---

Grc38\_ENST00000521276|miRNA

---

Grc38\_ENST00000532619|lincRNA

---

Grc38\_ENST00000550268|lincRNA

---

Grc38\_ENST00000551631|processed\_transcript

---

Grc38\_ENST00000559298|lincRNA

---

Grc38\_ENST00000578242|miRNA

---

Grc38\_ENST00000578311|miRNA

---

Grc38\_ENST00000579844|miRNA

---

Grc38\_ENST00000579969|snoRNA

---

Grc38\_ENST00000583823|snoRNA

---

Grc38\_ENST00000584034|miRNA

---

Grc38\_ENST00000585078|snoRNA

---

Grc38\_ENST00000597780|sense\_intronic

---

Grc38\_ENST00000606229|sense\_intronic

---

Grc38\_ENST00000606526|snoRNA

---

Grc38\_ENST00000606724|miRNA

---

Grc38\_ENST00000608229|lincRNA

---

Grc38\_ENST00000611454|snRNA

---

Grc38\_ENST00000613376|lincRNA

---

Grc38\_ENST00000617791|lincRNA

---

Grc38\_ENST00000623290|lincRNA

---

Grc38\_ENST00000624184|sense\_overlapping

---

Grc38\_ENST00000627071|lincRNA

---

Grc38\_ENST00000629919|miRNA

---

mirBASE\_sha-miR-21

---

piRNA|DQ582566

---

piRNA|DQ594740

---

Grc38\_ENST00000339037|lincRNA

---

Grc38\_ENST00000363314|snRNA

---

Grc38\_ENST00000363327|snRNA

---

Grc38\_ENST00000383902|snRNA

---

Grc38\_ENST00000384181|snRNA

---

Grc38\_ENST00000384328|snRNA

---

Grc38\_ENST00000384472|snRNA

---

Grc38\_ENST00000384476|snRNA

---

Grc38\_ENST00000384610|snRNA

---

Grc38\_ENST00000384770|snRNA

---

Grc38\_ENST00000411247|snRNA

---

Grc38\_ENST00000441255|lincRNA

---

Grc38\_ENST00000457339|lincRNA

---

Grc38\_ENST00000508096|sense\_overlapping

---

Grc38\_ENST00000516337|miRNA

---

Grc38\_ENST00000516502|snRNA

---

Grc38\_ENST00000516659|snRNA

---

Grc38\_ENST00000517228|miRNA

---

Grc38\_ENST00000517280|snRNA

---

Grc38\_ENST00000528381|lincRNA

---

Grc38\_ENST00000529733|lincRNA

---

Grc38\_ENST00000550723|lincRNA

---

Grc38\_ENST00000562127|lincRNA

---

Grc38\_ENST00000579051|miRNA

---

Grc38\_ENST00000584185|miRNA

---

Grc38\_ENST00000602813|lincRNA

---

Grc38\_ENST00000606801|snRNA

---

Grc38\_ENST00000612638|miRNA

---

Grc38\_ENST00000613023|snRNA

---

Grc38\_ENST00000614084|miRNA

---

Grc38\_ENST00000614774|snRNA

---

Grc38\_ENST00000617733|miRNA

---

Grc38\_ENST00000619109|snRNA

---

Grc38\_ENST00000623027|lincRNA

---

Grc38\_ENST00000628107|miRNA

---

Grc38\_ENST00000630744|miRNA

---

piRNA|DQ572769

---

piRNA|DQ590139

---

piRNA|DQ597968

---

piRNA|DQ599478

**Elements in Diff Represented in CAF-CELL vs CAF-EXO and Not Significant in CAF-CELL vs CAF-EXO**

**Elements in Not Significant in NF-CELL vs NF-EXO and Not Significant in CAF-CELL vs CAF-EXO**
  


---

Grc38\_ENST00000242109|lincRNA

---

Grc38\_ENST00000291374|lincRNA

---

Grc38\_ENST00000313064|lincRNA

---

Grc38\_ENST00000313303|processed\_transcript

---

Grc38\_ENST00000313807|lincRNA

---

Grc38\_ENST00000314957|sense\_overlapping

---

Grc38\_ENST00000316124|lincRNA

---

Grc38\_ENST00000316786|lincRNA

---

Grc38\_ENST00000318186|processed\_transcript

---

Grc38\_ENST00000322227|processed\_transcript

---

Grc38\_ENST00000324446|lincRNA

---

Grc38\_ENST00000324858|processed\_transcript

---

Grc38\_ENST00000333139|lincRNA

---

Grc38\_ENST00000334146|lincRNA

---

Grc38\_ENST00000341011|lincRNA

---

Grc38\_ENST00000344893|lincRNA

---

Grc38\_ENST00000347264|snRNA

---

Grc38\_ENST00000347538|miRNA

---

Grc38\_ENST00000352367|processed\_transcript

---

Grc38\_ENST00000355837|processed\_transcript

---

Grc38\_ENST00000356684|lincRNA

---

Grc38\_ENST00000357412|lincRNA

---

Grc38\_ENST00000358446|lincRNA

---

Grc38\_ENST00000358748|lincRNA

---

Grc38\_ENST00000359760|lincRNA

---

Grc38\_ENST00000360083|lincRNA

---

Grc38\_ENST00000362112|miRNA

---

Grc38\_ENST00000362177|miRNA

---

Grc38\_ENST00000362196|miRNA

---

Grc38\_ENST00000362199|miRNA

---

Grc38\_ENST00000362201|miRNA

---

Grc38\_ENST00000362212|miRNA

---

Grc38\_ENST00000362220|miRNA

---

Grc38\_ENST00000362237|miRNA

---

Grc38\_ENST00000362288|miRNA

---

Grc38\_ENST00000362423|snoRNA

---

Grc38\_ENST00000362535|snoRNA

---

Grc38\_ENST00000362566|snoRNA

---

Grc38\_ENST00000362627|snRNA

---

Grc38\_ENST00000362759|snRNA

---

Grc38\_ENST00000362817|snRNA

---

Grc38\_ENST00000362839|snRNA

---

Grc38\_ENST00000362915|snoRNA

---

Grc38\_ENST00000362928|snoRNA

---

Grc38\_ENST00000362935|snRNA

---

Grc38\_ENST00000362942|snRNA

---

Grc38\_ENST00000362986|snoRNA

---

Grc38\_ENST00000363009|snRNA

---

Grc38\_ENST00000363010|snRNA

---

Grc38\_ENST00000363036|snRNA

---

Grc38\_ENST00000363044|snoRNA

---

Grc38\_ENST00000363107|snoRNA

---

Grc38\_ENST00000363156|snoRNA

---

Grc38\_ENST00000363192|snRNA

---

Grc38\_ENST00000363237|snRNA

---

Grc38\_ENST00000363313|snRNA

---

Grc38\_ENST00000363319|snRNA

---

Grc38\_ENST00000363336|snoRNA

---

Grc38\_ENST00000363354|snRNA

---

Grc38\_ENST00000363367|snoRNA

---

Grc38\_ENST00000363409|snoRNA

---

Grc38\_ENST00000363448|snoRNA

---

Grc38\_ENST00000363484|snoRNA

---

Grc38\_ENST00000363506|snRNA

---

Grc38\_ENST00000363507|snoRNA

---

Grc38\_ENST00000363508|snRNA

---

Grc38\_ENST00000363750|snoRNA

---

Grc38\_ENST00000363762|snoRNA

---

Grc38\_ENST00000363791|snoRNA

---

Grc38\_ENST00000363915|snoRNA

---

Grc38\_ENST00000363919|snRNA

---

Grc38\_ENST00000363930|snRNA

---

Grc38\_ENST00000363937|snRNA

---

Grc38\_ENST00000363944|snRNA

---

Grc38\_ENST00000364015|snRNA

---

Grc38\_ENST00000364019|snRNA

---

Grc38\_ENST00000364033|snRNA

---

Grc38\_ENST00000364079|snoRNA

---

Grc38\_ENST00000364105|snRNA

---

Grc38\_ENST00000364116|snRNA

---

Grc38\_ENST00000364127|snRNA

---

Grc38\_ENST00000364249|snRNA

---

Grc38\_ENST00000364314|snRNA

---

Grc38\_ENST00000364329|snoRNA

---

Grc38\_ENST00000364359|snoRNA

---

Grc38\_ENST00000364377|snoRNA

---

Grc38\_ENST00000364383|snRNA

---

Grc38\_ENST00000364393|snoRNA

---

Grc38\_ENST00000364401|snRNA

---

Grc38\_ENST00000364478|snoRNA

---

Grc38\_ENST00000364516|snRNA

---

Grc38\_ENST00000364528|snoRNA

---

Grc38\_ENST00000364587|snoRNA

---

Grc38\_ENST00000364588|snRNA

---

Grc38\_ENST00000364688|snRNA

---

Grc38\_ENST00000364722|snoRNA

---

Grc38\_ENST00000364739|snRNA

---

Grc38\_ENST00000364749|snoRNA

---

Grc38\_ENST00000364758|snRNA

---

Grc38\_ENST00000364789|snRNA

---

Grc38\_ENST00000364819|snoRNA

---

Grc38\_ENST00000364830|snoRNA

---

Grc38\_ENST00000364874|snRNA

---

Grc38\_ENST00000364902|snoRNA

---

Grc38\_ENST00000364935|snRNA

---

Grc38\_ENST00000364951|snRNA

---

Grc38\_ENST00000364957|snoRNA

---

Grc38\_ENST00000364968|snoRNA

---

Grc38\_ENST00000365029|snoRNA

---

Grc38\_ENST00000365037|snoRNA

---

Grc38\_ENST00000365040|snRNA

---

Grc38\_ENST00000365075|snoRNA

---

Grc38\_ENST00000365379|snRNA

---

Grc38\_ENST00000365519|snoRNA

---

Grc38\_ENST00000365628|snoRNA

---

Grc38\_ENST00000365664|snRNA

---

Grc38\_ENST00000365690|miRNA

---

Grc38\_ENST00000365696|miRNA

---

Grc38\_ENST00000365739|miRNA

---

Grc38\_ENST00000366181|lincRNA

---

Grc38\_ENST00000366185|lincRNA

---

Grc38\_ENST00000366314|lincRNA

---

Grc38\_ENST00000366424|lincRNA

---

Grc38\_ENST00000366441|processed\_transcript

---

Grc38\_ENST00000369605|processed\_transcript

---

Grc38\_ENST00000371086|sense\_intronic

---

Grc38\_ENST00000371162|lincRNA

---

Grc38\_ENST00000373508|lincRNA

---

Grc38\_ENST00000374945|lincRNA

---

Grc38\_ENST00000375644|lincRNA

---

Grc38\_ENST00000379053|lincRNA

---

Grc38\_ENST00000379640|lincRNA

---

Grc38\_ENST00000381108|lincRNA

---

Grc38\_ENST00000381217|lincRNA

---

Grc38\_ENST00000382641|lincRNA

---

Grc38\_ENST00000383894|snoRNA

---

Grc38\_ENST00000383895|snoRNA

---

Grc38\_ENST00000383897|snRNA

---

Grc38\_ENST00000383907|snoRNA

---

Grc38\_ENST00000383934|snoRNA

---

Grc38\_ENST00000383961|snoRNA

---

Grc38\_ENST00000383968|snRNA

---

Grc38\_ENST00000383991|snRNA

---

Grc38\_ENST00000384020|snRNA

---

Grc38\_ENST00000384072|snoRNA

---

Grc38\_ENST00000384075|snRNA

---

Grc38\_ENST00000384107|snoRNA

---

Grc38\_ENST00000384111|snoRNA

---

Grc38\_ENST00000384129|snRNA

---

Grc38\_ENST00000384154|snRNA

---

Grc38\_ENST00000384210|snoRNA

---

Grc38\_ENST00000384274|snoRNA

---

Grc38\_ENST00000384323|snoRNA

---

Grc38\_ENST00000384325|snRNA

---

Grc38\_ENST00000384332|snRNA

---

Grc38\_ENST00000384337|snRNA

---

Grc38\_ENST00000384370|snoRNA

---

Grc38\_ENST00000384372|snoRNA

---

Grc38\_ENST00000384382|snRNA

---

Grc38\_ENST00000384430|snoRNA

---

Grc38\_ENST00000384439|snoRNA

---

Grc38\_ENST00000384445|snoRNA

---

Grc38\_ENST00000384453|snRNA

---

Grc38\_ENST00000384462|snoRNA

---

Grc38\_ENST00000384470|snoRNA

---

Grc38\_ENST00000384507|snoRNA

---

Grc38\_ENST00000384516|snoRNA

---

Grc38\_ENST00000384529|snoRNA

---

Grc38\_ENST00000384531|snRNA

---

Grc38\_ENST00000384533|snoRNA

---

Grc38\_ENST00000384549|snoRNA

---

Grc38\_ENST00000384561|snRNA

---

Grc38\_ENST00000384567|snoRNA

---

Grc38\_ENST00000384582|snRNA

---

Grc38\_ENST00000384598|snRNA

---

Grc38\_ENST00000384613|snRNA

---

Grc38\_ENST00000384614|snoRNA

---

Grc38\_ENST00000384645|snoRNA

---

Grc38\_ENST00000384675|snoRNA

---

Grc38\_ENST00000384710|snRNA

---

Grc38\_ENST00000384729|snoRNA

---

Grc38\_ENST00000384737|snoRNA

---

Grc38\_ENST00000384744|snoRNA

---

Grc38\_ENST00000384771|snoRNA

---

Grc38\_ENST00000384819|miRNA

---

Grc38\_ENST00000384827|miRNA

---

Grc38\_ENST00000384837|miRNA

---

Grc38\_ENST00000384839|miRNA

---

Grc38\_ENST00000384851|miRNA

---

Grc38\_ENST00000384870|miRNA

---

Grc38\_ENST00000384873|miRNA

---

Grc38\_ENST00000384875|miRNA

---

Grc38\_ENST00000384878|miRNA

---

Grc38\_ENST00000384882|miRNA

---

Grc38\_ENST00000384887|miRNA

---

Grc38\_ENST00000384891|miRNA

---

Grc38\_ENST00000384903|miRNA

---

Grc38\_ENST00000384916|miRNA

---

Grc38\_ENST00000384920|miRNA

---

Grc38\_ENST00000384921|miRNA

---

Grc38\_ENST00000384961|miRNA

---

Grc38\_ENST00000384972|miRNA

---

Grc38\_ENST00000384973|miRNA

---

Grc38\_ENST00000384980|miRNA

---

Grc38\_ENST00000384981|miRNA

---

Grc38\_ENST00000384997|miRNA

---

Grc38\_ENST00000384998|miRNA

---

Grc38\_ENST00000385002|miRNA

---

Grc38\_ENST00000385008|miRNA

---

Grc38\_ENST00000385018|miRNA

---

Grc38\_ENST00000385032|miRNA

---

Grc38\_ENST00000385037|miRNA

---

Grc38\_ENST00000385047|miRNA

---

Grc38\_ENST00000385048|miRNA

---

Grc38\_ENST00000385052|miRNA

---

Grc38\_ENST00000385056|miRNA

---

Grc38\_ENST00000385065|miRNA

---

Grc38\_ENST00000385070|miRNA

---

Grc38\_ENST00000385104|miRNA

---

Grc38\_ENST00000385160|miRNA

---

Grc38\_ENST00000385189|miRNA

---

Grc38\_ENST00000385194|miRNA

---

Grc38\_ENST00000385197|miRNA

---

Grc38\_ENST00000385200|miRNA

---

Grc38\_ENST00000385201|miRNA

---

Grc38\_ENST00000385204|miRNA

---

Grc38\_ENST00000385205|miRNA

---

Grc38\_ENST00000385213|miRNA

---

Grc38\_ENST00000385219|miRNA

---

Grc38\_ENST00000385224|miRNA

---

Grc38\_ENST00000385226|miRNA

---

Grc38\_ENST00000385229|miRNA

---

Grc38\_ENST00000385238|miRNA

---

Grc38\_ENST00000385253|miRNA

---

Grc38\_ENST00000385259|miRNA

---

Grc38\_ENST00000385261|miRNA

---

Grc38\_ENST00000385268|miRNA

---

Grc38\_ENST00000385269|miRNA

---

Grc38\_ENST00000385287|miRNA

---

Grc38\_ENST00000385292|miRNA

---

Grc38\_ENST00000386910|snoRNA

---

Grc38\_ENST00000388006|miRNA

---

Grc38\_ENST00000388090|snoRNA

---

Grc38\_ENST00000388106|snRNA

---

Grc38\_ENST00000388402|miRNA

---

Grc38\_ENST00000390165|miRNA

---

Grc38\_ENST00000390179|miRNA

---

Grc38\_ENST00000390184|miRNA

---

Grc38\_ENST00000390209|miRNA

---

Grc38\_ENST00000390219|miRNA

---

Grc38\_ENST00000390220|miRNA

---

Grc38\_ENST00000390756|miRNA

---

Grc38\_ENST00000390783|miRNA

---

Grc38\_ENST00000390813|miRNA

---

Grc38\_ENST00000390846|snoRNA

---

Grc38\_ENST00000390868|snRNA

---

Grc38\_ENST00000390870|snRNA

---

Grc38\_ENST00000390880|snoRNA

---

Grc38\_ENST00000390904|snoRNA

---

Grc38\_ENST00000390909|snoRNA

---

Grc38\_ENST00000390924|snRNA

---

Grc38\_ENST00000390994|snoRNA

---

Grc38\_ENST00000391042|snRNA

---

Grc38\_ENST00000391052|snRNA

---

Grc38\_ENST00000391078|snoRNA

---

Grc38\_ENST00000391250|snoRNA

---

Grc38\_ENST00000391270|snRNA

---

Grc38\_ENST00000391279|snoRNA

---

Grc38\_ENST00000391303|snRNA

---

Grc38\_ENST00000391307|snRNA

---

Grc38\_ENST00000391308|snoRNA

---

Grc38\_ENST00000391313|snoRNA

---

Grc38\_ENST00000391625|lincRNA

---

Grc38\_ENST00000392385|lincRNA

---

Grc38\_ENST00000393264|lincRNA

---

Grc38\_ENST00000395400|processed\_transcript

---

Grc38\_ENST00000396880|sense\_intronic

---

Grc38\_ENST00000397057|sense\_overlapping

---

Grc38\_ENST00000398461|lincRNA

---

Grc38\_ENST00000398474|lincRNA

---

Grc38\_ENST00000398957|sense\_intronic

---

Grc38\_ENST00000399003|lincRNA

---

Grc38\_ENST00000399242|sense\_intronic

---

Grc38\_ENST00000399387|lincRNA

---

Grc38\_ENST00000399449|processed\_transcript

---

Grc38\_ENST00000399711|lincRNA

---

Grc38\_ENST00000399866|lincRNA

---

Grc38\_ENST00000400593|lincRNA

---

Grc38\_ENST00000401018|lincRNA

---

Grc38\_ENST00000401104|miRNA

---

Grc38\_ENST00000401111|miRNA

---

Grc38\_ENST00000401112|miRNA

---

Grc38\_ENST00000401126|miRNA

---

Grc38\_ENST00000401134|miRNA

---

Grc38\_ENST00000401135|miRNA

---

Grc38\_ENST00000401141|miRNA

---

Grc38\_ENST00000401157|miRNA

---

Grc38\_ENST00000401164|miRNA

---

Grc38\_ENST00000401258|miRNA

---

Grc38\_ENST00000401269|miRNA

---

Grc38\_ENST00000401276|miRNA

---

Grc38\_ENST00000401280|miRNA

---

Grc38\_ENST00000401297|miRNA

---

Grc38\_ENST00000401360|miRNA

---

Grc38\_ENST00000401372|miRNA

---

Grc38\_ENST00000408078|miRNA

---

Grc38\_ENST00000408103|miRNA

---

Grc38\_ENST00000408110|miRNA

---

Grc38\_ENST00000408124|miRNA

---

Grc38\_ENST00000408135|miRNA

---

Grc38\_ENST00000408154|miRNA

---

Grc38\_ENST00000408196|miRNA

---

Grc38\_ENST00000408203|miRNA

---

Grc38\_ENST00000408236|miRNA

---

Grc38\_ENST00000408243|miRNA

---

Grc38\_ENST00000408259|miRNA

---

Grc38\_ENST00000408276|miRNA

---

Grc38\_ENST00000408310|miRNA

---

Grc38\_ENST00000408311|miRNA

---

Grc38\_ENST00000408337|miRNA

---

Grc38\_ENST00000408338|miRNA

---

Grc38\_ENST00000408359|miRNA

---

Grc38\_ENST00000408369|miRNA

---

Grc38\_ENST00000408406|miRNA

---

Grc38\_ENST00000408438|miRNA

---

Grc38\_ENST00000408439|miRNA

---

Grc38\_ENST00000408467|miRNA

---

Grc38\_ENST00000408468|miRNA

---

Grc38\_ENST00000408479|miRNA

---

Grc38\_ENST00000408518|miRNA

---

Grc38\_ENST00000408520|miRNA

---

Grc38\_ENST00000408521|miRNA

---

Grc38\_ENST00000408534|snoRNA

---

Grc38\_ENST00000408536|miRNA

---

Grc38\_ENST00000408537|miRNA

---

Grc38\_ENST00000408566|miRNA

---

Grc38\_ENST00000408569|snoRNA

---

Grc38\_ENST00000408575|miRNA

---

Grc38\_ENST00000408583|miRNA

---

Grc38\_ENST00000408593|miRNA

---

Grc38\_ENST00000408601|miRNA

---

Grc38\_ENST00000408613|miRNA

---

Grc38\_ENST00000408637|snRNA

---

Grc38\_ENST00000408658|miRNA

---

Grc38\_ENST00000408662|miRNA

---

Grc38\_ENST00000408683|miRNA

---

Grc38\_ENST00000408685|miRNA

---

Grc38\_ENST00000408692|miRNA

---

Grc38\_ENST00000408704|miRNA

---

Grc38\_ENST00000408716|snoRNA

---

Grc38\_ENST00000408729|miRNA

---

Grc38\_ENST00000408735|miRNA

---

Grc38\_ENST00000408737|miRNA

---

Grc38\_ENST00000408789|snoRNA

---

Grc38\_ENST00000408805|miRNA

---

Grc38\_ENST00000408806|miRNA

---

Grc38\_ENST00000408818|miRNA

---

Grc38\_ENST00000408840|miRNA

---

Grc38\_ENST00000408847|miRNA

---

Grc38\_ENST00000408865|miRNA

---

Grc38\_ENST00000408876|snoRNA

---

Grc38\_ENST00000409590|lincRNA

---

Grc38\_ENST00000410162|snRNA

---

Grc38\_ENST00000410194|snRNA

---

Grc38\_ENST00000410207|snRNA

---

Grc38\_ENST00000410253|snoRNA

---

Grc38\_ENST00000410287|miRNA

---

Grc38\_ENST00000410290|snRNA

---

Grc38\_ENST00000410294|miRNA

---

Grc38\_ENST00000410299|snRNA

---

Grc38\_ENST00000410306|snRNA

---

Grc38\_ENST00000410344|snRNA

---

Grc38\_ENST00000410394|miRNA

---

Grc38\_ENST00000410423|snRNA

---

Grc38\_ENST00000410425|snRNA

---

Grc38\_ENST00000410438|snoRNA

---

Grc38\_ENST00000410507|snRNA

---

Grc38\_ENST00000410554|snRNA

---

Grc38\_ENST00000410600|miRNA

---

Grc38\_ENST00000410604|snRNA

---

Grc38\_ENST00000410650|snRNA

---

Grc38\_ENST00000410675|snRNA

---

Grc38\_ENST00000410680|snRNA

---

Grc38\_ENST00000410694|snRNA

---

Grc38\_ENST00000410708|snRNA

---

Grc38\_ENST00000410718|snRNA

---

Grc38\_ENST00000410727|snRNA

---

Grc38\_ENST00000410754|snRNA

---

Grc38\_ENST00000410792|snRNA

---

Grc38\_ENST00000410829|snRNA

---

Grc38\_ENST00000410845|snRNA

---

Grc38\_ENST00000410858|snRNA

---

Grc38\_ENST00000410878|snRNA

---

Grc38\_ENST00000410889|snRNA

---

Grc38\_ENST00000410967|miRNA

---

Grc38\_ENST00000411054|snRNA

---

Grc38\_ENST00000411069|snRNA

---

Grc38\_ENST00000411164|snRNA

---

Grc38\_ENST00000411175|snRNA

---

Grc38\_ENST00000411193|snRNA

---

Grc38\_ENST00000411224|snRNA

---

Grc38\_ENST00000411381|snRNA

---

Grc38\_ENST00000411436|lincRNA

---

Grc38\_ENST00000411553|lincRNA

---

Grc38\_ENST00000411575|lincRNA

---

Grc38\_ENST00000411895|sense\_intronic

---

Grc38\_ENST00000411921|processed\_transcript

---

Grc38\_ENST00000412068|lincRNA

---

Grc38\_ENST00000412242|sense\_intronic

---

Grc38\_ENST00000412422|lincRNA

---

Grc38\_ENST00000412553|lincRNA

---

Grc38\_ENST00000412816|lincRNA

---

Grc38\_ENST00000413042|lincRNA

---

Grc38\_ENST00000413053|lincRNA

---

Grc38\_ENST00000413291|lincRNA

---

Grc38\_ENST00000413842|processed\_transcript

---

Grc38\_ENST00000413862|processed\_transcript

---

Grc38\_ENST00000413935|processed\_transcript

---

Grc38\_ENST00000413987|sense\_intronic

---

Grc38\_ENST00000414159|lincRNA

---

Grc38\_ENST00000414175|processed\_transcript

---

Grc38\_ENST00000414209|lincRNA

---

Grc38\_ENST00000414227|sense\_overlapping

---

Grc38\_ENST00000414287|lincRNA

---

Grc38\_ENST00000414562|lincRNA

---

Grc38\_ENST00000414662|processed\_transcript

---

Grc38\_ENST00000414786|processed\_transcript

---

Grc38\_ENST00000414816|lincRNA

---

Grc38\_ENST00000414890|sense\_intronic

---

Grc38\_ENST00000414948|sense\_intronic

---

Grc38\_ENST00000415019|lincRNA

---

Grc38\_ENST00000415150|processed\_transcript

---

Grc38\_ENST00000415338|lincRNA

---

Grc38\_ENST00000415663|sense\_intronic

---

Grc38\_ENST00000415801|lincRNA

---

Grc38\_ENST00000416191|lincRNA

---

Grc38\_ENST00000416381|lincRNA

---

Grc38\_ENST00000416385|lincRNA

---

Grc38\_ENST00000416689|lincRNA

---

Grc38\_ENST00000416982|lincRNA

---

Grc38\_ENST00000417120|sense\_intronic

---

Grc38\_ENST00000417218|lincRNA

---

Grc38\_ENST00000417483|lincRNA

---

Grc38\_ENST00000418006|lincRNA

---

Grc38\_ENST00000418344|lincRNA

---

Grc38\_ENST00000418534|lincRNA

---

Grc38\_ENST00000418539|lincRNA

---

Grc38\_ENST00000418591|lincRNA

---

Grc38\_ENST00000418679|lincRNA

---

Grc38\_ENST00000418821|lincRNA

---

Grc38\_ENST00000418839|lincRNA

---

Grc38\_ENST00000419160|lincRNA

---

Grc38\_ENST00000419272|lincRNA

---

Grc38\_ENST00000419300|processed\_transcript

---

Grc38\_ENST00000419362|lincRNA

---

Grc38\_ENST00000419458|lincRNA

---

Grc38\_ENST00000419745|lincRNA

---

Grc38\_ENST00000419766|processed\_transcript

---

Grc38\_ENST00000419784|lincRNA

---

Grc38\_ENST00000419853|lincRNA

---

Grc38\_ENST00000419895|lincRNA

---

Grc38\_ENST00000420255|lincRNA

---

Grc38\_ENST00000420364|sense\_overlapping

---

Grc38\_ENST00000420508|lincRNA

---

Grc38\_ENST00000420572|lincRNA

---

Grc38\_ENST00000420585|lincRNA

---

Grc38\_ENST00000420724|lincRNA

---

Grc38\_ENST00000420823|lincRNA

---

Grc38\_ENST00000420856|processed\_transcript

---

Grc38\_ENST00000420866|lincRNA

---

Grc38\_ENST00000420902|sense\_intronic

---

Grc38\_ENST00000420905|lincRNA

---

Grc38\_ENST00000420918|processed\_transcript

---

Grc38\_ENST00000421064|processed\_transcript

---

Grc38\_ENST00000421121|processed\_transcript

---

Grc38\_ENST00000421378|lincRNA

---

Grc38\_ENST00000421505|lincRNA

---

Grc38\_ENST00000421598|lincRNA

---

Grc38\_ENST00000421685|lincRNA

---

Grc38\_ENST00000421703|processed\_transcript

---

Grc38\_ENST00000421931|lincRNA

---

Grc38\_ENST00000421976|lincRNA

---

Grc38\_ENST00000422118|lincRNA

---

Grc38\_ENST00000422374|lincRNA

---

Grc38\_ENST00000422519|lincRNA

---

Grc38\_ENST00000422661|lincRNA

---

Grc38\_ENST00000422780|lincRNA

---

Grc38\_ENST00000423208|lincRNA

---

Grc38\_ENST00000423222|lincRNA

---

Grc38\_ENST00000423323|processed\_transcript

---

Grc38\_ENST00000423403|lincRNA

---

Grc38\_ENST00000423918|processed\_transcript

---

Grc38\_ENST00000424208|processed\_transcript

---

Grc38\_ENST00000424415|lincRNA

---

Grc38\_ENST00000424496|sense\_intronic

---

Grc38\_ENST00000424516|sense\_overlapping

---

Grc38\_ENST00000424684|lincRNA

---

Grc38\_ENST00000424850|lincRNA

---

Grc38\_ENST00000424943|lincRNA

---

Grc38\_ENST00000424982|sense\_intronic

---

Grc38\_ENST00000425170|processed\_transcript

---

Grc38\_ENST00000425211|processed\_transcript

---

Grc38\_ENST00000425296|sense\_intronic

---

Grc38\_ENST00000425388|lincRNA

---

Grc38\_ENST00000425397|processed\_transcript

---

Grc38\_ENST00000425493|lincRNA

---

Grc38\_ENST00000425648|processed\_transcript

---

Grc38\_ENST00000425800|lincRNA

---

Grc38\_ENST00000426066|processed\_transcript

---

Grc38\_ENST00000426283|lincRNA

---

Grc38\_ENST00000426529|processed\_transcript

---

Grc38\_ENST00000426585|lincRNA

---

Grc38\_ENST00000426635|lincRNA

---

Grc38\_ENST00000426812|processed\_transcript

---

Grc38\_ENST00000426991|sense\_intronic

---

Grc38\_ENST00000427391|lincRNA

---

Grc38\_ENST00000427804|lincRNA

---

Grc38\_ENST00000427824|lincRNA

---

Grc38\_ENST00000427872|lincRNA

---

Grc38\_ENST00000428191|sense\_intronic

---

Grc38\_ENST00000428222|lincRNA

---

Grc38\_ENST00000428647|lincRNA

---

Grc38\_ENST00000428761|lincRNA

---

Grc38\_ENST00000428833|processed\_transcript

---

Grc38\_ENST00000428891|sense\_intronic

---

Grc38\_ENST00000429010|lincRNA

---

Grc38\_ENST00000429067|processed\_transcript

---

Grc38\_ENST00000429137|lincRNA

---

Grc38\_ENST00000429367|lincRNA

---

Grc38\_ENST00000429953|lincRNA

---

Grc38\_ENST00000429998|processed\_transcript

---

Grc38\_ENST00000430034|3prime\_overlapping\_ncrna

---

Grc38\_ENST00000430050|lincRNA

---

Grc38\_ENST00000430922|lincRNA

---

Grc38\_ENST00000430998|lincRNA

---

Grc38\_ENST00000431060|processed\_transcript

---

Grc38\_ENST00000431712|lincRNA

---

Grc38\_ENST00000431737|lincRNA

---

Grc38\_ENST00000431813|sense\_intronic

---

Grc38\_ENST00000431979|lincRNA

---

Grc38\_ENST00000432045|lincRNA

---

Grc38\_ENST00000432047|sense\_intronic

---

Grc38\_ENST00000432230|lincRNA

---

Grc38\_ENST00000432330|lincRNA

---

Grc38\_ENST00000432429|lincRNA

---

Grc38\_ENST00000432474|lincRNA

---

Grc38\_ENST00000432536|processed\_transcript

---

Grc38\_ENST00000432668|processed\_transcript

---

Grc38\_ENST00000432783|lincRNA

---

Grc38\_ENST00000432984|lincRNA

---

Grc38\_ENST00000432988|processed\_transcript

---

Grc38\_ENST00000433082|processed\_transcript

---

Grc38\_ENST00000433116|lincRNA

---

Grc38\_ENST00000433669|lincRNA

---

Grc38\_ENST00000433724|lincRNA

---

Grc38\_ENST00000433843|processed\_transcript

---

Grc38\_ENST00000433856|lincRNA

---

Grc38\_ENST00000434244|lincRNA

---

Grc38\_ENST00000434540|lincRNA

---

Grc38\_ENST00000434859|lincRNA

---

Grc38\_ENST00000434868|lincRNA

---

Grc38\_ENST00000435311|lincRNA

---

Grc38\_ENST00000435411|lincRNA

---

Grc38\_ENST00000435597|lincRNA

---

Grc38\_ENST00000436200|processed\_transcript

---

Grc38\_ENST00000436416|sense\_overlapping

---

Grc38\_ENST00000436742|processed\_transcript

---

Grc38\_ENST00000436786|lincRNA

---

Grc38\_ENST00000436820|lincRNA

---

Grc38\_ENST00000436932|lincRNA

---

Grc38\_ENST00000437235|lincRNA

---

Grc38\_ENST00000437716|processed\_transcript

---

Grc38\_ENST00000437730|lincRNA

---

Grc38\_ENST00000437781|processed\_transcript

---

Grc38\_ENST00000437916|lincRNA

---

Grc38\_ENST00000437920|lincRNA

---

Grc38\_ENST00000438002|lincRNA

---

Grc38\_ENST00000438107|lincRNA

---

Grc38\_ENST00000438154|lincRNA

---

Grc38\_ENST00000438368|lincRNA

---

Grc38\_ENST00000438618|lincRNA

---

Grc38\_ENST00000439004|lincRNA

---

Grc38\_ENST00000439105|lincRNA

---

Grc38\_ENST00000439670|processed\_transcript

---

Grc38\_ENST00000439898|lincRNA

---

Grc38\_ENST00000439913|lincRNA

---

Grc38\_ENST00000440088|lincRNA

---

Grc38\_ENST00000440104|lincRNA

---

Grc38\_ENST00000440408|lincRNA

---

Grc38\_ENST00000440455|lincRNA

---

Grc38\_ENST00000440496|lincRNA

---

Grc38\_ENST00000440698|lincRNA

---

Grc38\_ENST00000440762|lincRNA

---

Grc38\_ENST00000440776|processed\_transcript

---

Grc38\_ENST00000441009|lincRNA

---

Grc38\_ENST00000441052|lincRNA

---

Grc38\_ENST00000441093|sense\_overlapping

---

Grc38\_ENST00000441095|lincRNA

---

Grc38\_ENST00000441167|sense\_intronic

---

Grc38\_ENST00000441272|lincRNA

---

Grc38\_ENST00000441587|sense\_intronic

---

Grc38\_ENST00000441932|lincRNA

---

Grc38\_ENST00000442116|lincRNA

---

Grc38\_ENST00000442133|processed\_transcript

---

Grc38\_ENST00000442197|lincRNA

---

Grc38\_ENST00000442318|lincRNA

---

Grc38\_ENST00000442456|lincRNA

---

Grc38\_ENST00000443205|lincRNA

---

Grc38\_ENST00000443243|lincRNA

---

Grc38\_ENST00000443554|lincRNA

---

Grc38\_ENST00000443836|lincRNA

---

Grc38\_ENST00000443926|lincRNA

---

Grc38\_ENST00000444037|lincRNA

---

Grc38\_ENST00000444042|lincRNA

---

Grc38\_ENST00000444210|lincRNA

---

Grc38\_ENST00000444431|processed\_transcript

---

Grc38\_ENST00000444482|lincRNA

---

Grc38\_ENST00000445300|lincRNA

---

Grc38\_ENST00000445332|lincRNA

---

Grc38\_ENST00000445770|lincRNA

---

Grc38\_ENST00000446159|processed\_transcript

---

Grc38\_ENST00000446358|lincRNA

---

Grc38\_ENST00000446516|lincRNA

---

Grc38\_ENST00000446912|lincRNA

---

Grc38\_ENST00000447198|lincRNA

---

Grc38\_ENST00000447413|sense\_overlapping

---

Grc38\_ENST00000447478|lincRNA

---

Grc38\_ENST00000447507|sense\_intronic

---

Grc38\_ENST00000447524|lincRNA

---

Grc38\_ENST00000447577|processed\_transcript

---

Grc38\_ENST00000447643|lincRNA

---

Grc38\_ENST00000447748|lincRNA

---

Grc38\_ENST00000447785|lincRNA

---

Grc38\_ENST00000447898|processed\_transcript

---

Grc38\_ENST00000448179|lincRNA

---

Grc38\_ENST00000448256|lincRNA

---

Grc38\_ENST00000448494|lincRNA

---

Grc38\_ENST00000448587|lincRNA

---

Grc38\_ENST00000448901|lincRNA

---

Grc38\_ENST00000448941|processed\_transcript

---

Grc38\_ENST00000449259|lincRNA

---

Grc38\_ENST00000449307|lincRNA

---

Grc38\_ENST00000449316|lincRNA

---

Grc38\_ENST00000449339|lincRNA

---

Grc38\_ENST00000449573|sense\_overlapping

---

Grc38\_ENST00000449882|lincRNA

---

Grc38\_ENST00000450709|processed\_transcript

---

Grc38\_ENST00000450804|lincRNA

---

Grc38\_ENST00000450977|lincRNA

---

Grc38\_ENST00000451289|lincRNA

---

Grc38\_ENST00000451298|processed\_transcript

---

Grc38\_ENST00000451302|processed\_transcript

---

Grc38\_ENST00000451317|lincRNA

---

Grc38\_ENST00000451355|sense\_intronic

---

Grc38\_ENST00000451400|lincRNA

---

Grc38\_ENST00000451707|lincRNA

---

Grc38\_ENST00000451762|processed\_transcript

---

Grc38\_ENST00000451905|lincRNA

---

Grc38\_ENST00000451980|lincRNA

---

Grc38\_ENST00000452120|lincRNA

---

Grc38\_ENST00000452197|processed\_transcript

---

Grc38\_ENST00000452354|lincRNA

---

Grc38\_ENST00000452431|processed\_transcript

---

Grc38\_ENST00000452622|lincRNA

---

Grc38\_ENST00000452623|processed\_transcript

---

Grc38\_ENST00000452643|lincRNA

---

Grc38\_ENST00000452690|lincRNA

---

Grc38\_ENST00000452787|lincRNA

---

Grc38\_ENST00000452834|lincRNA

---

Grc38\_ENST00000452840|lincRNA

---

Grc38\_ENST00000452982|lincRNA

---

Grc38\_ENST00000453082|processed\_transcript

---

Grc38\_ENST00000453159|processed\_transcript

---

Grc38\_ENST00000453209|processed\_transcript

---

Grc38\_ENST00000453420|lincRNA

---

Grc38\_ENST00000453554|lincRNA

---

Grc38\_ENST00000453754|processed\_transcript

---

Grc38\_ENST00000453837|lincRNA

---

Grc38\_ENST00000454411|lincRNA

---

Grc38\_ENST00000454596|lincRNA

---

Grc38\_ENST00000454832|sense\_intronic

---

Grc38\_ENST00000454922|processed\_transcript

---

Grc38\_ENST00000454932|processed\_transcript

---

Grc38\_ENST00000454935|lincRNA

---

Grc38\_ENST00000455336|sense\_intronic

---

Grc38\_ENST00000455531|lincRNA

---

Grc38\_ENST00000455557|sense\_overlapping

---

Grc38\_ENST00000455945|processed\_transcript

---

Grc38\_ENST00000456100|processed\_transcript

---

Grc38\_ENST00000456248|lincRNA

---

Grc38\_ENST00000456265|lincRNA

---

Grc38\_ENST00000456477|lincRNA

---

Grc38\_ENST00000456576|processed\_transcript

---

Grc38\_ENST00000456601|processed\_transcript

---

Grc38\_ENST00000456627|lincRNA

---

Grc38\_ENST00000456746|sense\_overlapping

---

Grc38\_ENST00000456782|sense\_intronic

---

Grc38\_ENST00000456895|lincRNA

---

Grc38\_ENST00000456917|lincRNA

---

Grc38\_ENST00000457009|lincRNA

---

Grc38\_ENST00000457025|processed\_transcript

---

Grc38\_ENST00000457033|processed\_transcript

---

Grc38\_ENST00000457321|lincRNA

---

Grc38\_ENST00000457348|lincRNA

---

Grc38\_ENST00000458007|3prime\_overlapping\_ncrna

---

Grc38\_ENST00000458392|lincRNA

---

Grc38\_ENST00000458762|snoRNA

---

Grc38\_ENST00000458790|snoRNA

---

Grc38\_ENST00000458811|snRNA

---

Grc38\_ENST00000458841|snRNA

---

Grc38\_ENST00000459128|snoRNA

---

Grc38\_ENST00000459157|snoRNA

---

Grc38\_ENST00000459229|snoRNA

---

Grc38\_ENST00000459249|snRNA

---

Grc38\_ENST00000459334|snRNA

---

Grc38\_ENST00000459421|miRNA

---

Grc38\_ENST00000459500|snRNA

---

Grc38\_ENST00000459538|snoRNA

---

Grc38\_ENST00000459577|snoRNA

---

Grc38\_ENST00000460754|sense\_intronic

---

Grc38\_ENST00000460977|sense\_overlapping

---

Grc38\_ENST00000462662|processed\_transcript

---

Grc38\_ENST00000462959|processed\_transcript

---

Grc38\_ENST00000463183|lincRNA

---

Grc38\_ENST00000463508|miRNA

---

Grc38\_ENST00000465215|lincRNA

---

Grc38\_ENST00000465933|lincRNA

---

Grc38\_ENST00000466430|lincRNA

---

Grc38\_ENST00000468219|sense\_intronic

---

Grc38\_ENST00000468851|miRNA

---

Grc38\_ENST00000471090|lincRNA

---

Grc38\_ENST00000472120|lincRNA

---

Grc38\_ENST00000472323|lincRNA

---

Grc38\_ENST00000472367|processed\_transcript

---

Grc38\_ENST00000472655|lincRNA

---

Grc38\_ENST00000473595|lincRNA

---

Grc38\_ENST00000474045|lincRNA

---

Grc38\_ENST00000477392|lincRNA

---

Grc38\_ENST00000478103|processed\_transcript

---

Grc38\_ENST00000478845|sense\_intronic

---

Grc38\_ENST00000481027|processed\_transcript

---

Grc38\_ENST00000481893|processed\_transcript

---

Grc38\_ENST00000482382|lincRNA

---

Grc38\_ENST00000482787|lincRNA

---

Grc38\_ENST00000482985|lincRNA

---

Grc38\_ENST00000483140|processed\_transcript

---

Grc38\_ENST00000483544|lincRNA

---

Grc38\_ENST00000483840|lincRNA

---

Grc38\_ENST00000484076|lincRNA

---

Grc38\_ENST00000484550|lincRNA

---

Grc38\_ENST00000484698|lincRNA

---

Grc38\_ENST00000484892|lincRNA

---

Grc38\_ENST00000486571|lincRNA

---

Grc38\_ENST00000488123|miRNA

---

Grc38\_ENST00000489336|processed\_transcript

---

Grc38\_ENST00000489670|lincRNA

---

Grc38\_ENST00000490357|lincRNA

---

Grc38\_ENST00000491608|lincRNA

---

Grc38\_ENST00000492465|processed\_transcript

---

Grc38\_ENST00000492981|lincRNA

---

Grc38\_ENST00000494270|processed\_transcript

---

Grc38\_ENST00000494582|lincRNA

---

Grc38\_ENST00000496403|lincRNA

---

Grc38\_ENST00000497248|processed\_transcript

---

Grc38\_ENST00000497988|sense\_intronic

---

Grc38\_ENST00000498032|lincRNA

---

Grc38\_ENST00000498714|sense\_intronic

---

Grc38\_ENST00000498967|lincRNA

---

Grc38\_ENST00000499006|lincRNA

---

Grc38\_ENST00000499025|lincRNA

---

Grc38\_ENST00000499137|processed\_transcript

---

Grc38\_ENST00000499291|lincRNA

---

Grc38\_ENST00000499418|lincRNA

---

Grc38\_ENST00000499452|lincRNA

---

Grc38\_ENST00000499499|lincRNA

---

Grc38\_ENST00000499502|lincRNA

---

Grc38\_ENST00000499521|processed\_transcript

---

Grc38\_ENST00000499732|lincRNA

---

Grc38\_ENST00000499797|lincRNA

---

Grc38\_ENST00000499809|lincRNA

---

Grc38\_ENST00000500009|lincRNA

---

Grc38\_ENST00000500016|lincRNA

---

Grc38\_ENST00000500112|lincRNA

---

Grc38\_ENST00000500197|lincRNA

---

Grc38\_ENST00000500215|lincRNA

---

Grc38\_ENST00000500698|lincRNA

---

Grc38\_ENST00000500850|lincRNA

---

Grc38\_ENST00000500853|processed\_transcript

---

Grc38\_ENST00000500941|lincRNA

---

Grc38\_ENST00000500949|processed\_transcript

---

Grc38\_ENST00000501259|lincRNA

---

Grc38\_ENST00000501499|lincRNA

---

Grc38\_ENST00000501520|lincRNA

---

Grc38\_ENST00000501541|lincRNA

---

Grc38\_ENST00000501708|lincRNA

---

Grc38\_ENST00000501817|lincRNA

---

Grc38\_ENST00000501937|lincRNA

---

Grc38\_ENST00000501965|lincRNA

---

Grc38\_ENST00000502071|lincRNA

---

Grc38\_ENST00000502390|lincRNA

---

Grc38\_ENST00000502417|lincRNA

---

Grc38\_ENST00000502421|lincRNA

---

Grc38\_ENST00000502479|lincRNA

---

Grc38\_ENST00000502934|lincRNA

---

Grc38\_ENST00000503695|lincRNA

---

Grc38\_ENST00000503985|lincRNA

---

Grc38\_ENST00000504184|lincRNA

---

Grc38\_ENST00000504409|lincRNA

---

Grc38\_ENST00000504876|lincRNA

---

Grc38\_ENST00000505109|lincRNA

---

Grc38\_ENST00000505289|lincRNA

---

Grc38\_ENST00000505556|lincRNA

---

Grc38\_ENST00000505642|processed\_transcript

---

Grc38\_ENST00000505668|sense\_intronic

---

Grc38\_ENST00000505807|processed\_transcript

---

Grc38\_ENST00000505870|lincRNA

---

Grc38\_ENST00000506090|lincRNA

---

Grc38\_ENST00000506222|lincRNA

---

Grc38\_ENST00000506299|lincRNA

---

Grc38\_ENST00000506305|lincRNA

---

Grc38\_ENST00000506314|processed\_transcript

---

Grc38\_ENST00000506655|lincRNA

---

Grc38\_ENST00000506723|lincRNA

---

Grc38\_ENST00000506950|sense\_intronic

---

Grc38\_ENST00000507031|lincRNA

---

Grc38\_ENST00000507296|lincRNA

---

Grc38\_ENST00000507311|lincRNA

---

Grc38\_ENST00000507313|lincRNA

---

Grc38\_ENST00000507365|lincRNA

---

Grc38\_ENST00000507938|sense\_overlapping

---

Grc38\_ENST00000508106|lincRNA

---

Grc38\_ENST00000508202|lincRNA

---

Grc38\_ENST00000508309|lincRNA

---

Grc38\_ENST00000508313|lincRNA

---

Grc38\_ENST00000508713|sense\_intronic

---

Grc38\_ENST00000508827|lincRNA

---

Grc38\_ENST00000508969|processed\_transcript

---

Grc38\_ENST00000509015|lincRNA

---

Grc38\_ENST00000509506|lincRNA

---

Grc38\_ENST00000509654|lincRNA

---

Grc38\_ENST00000509921|lincRNA

---

Grc38\_ENST00000509964|lincRNA

---

Grc38\_ENST00000509983|lincRNA

---

Grc38\_ENST00000510371|lincRNA

---

Grc38\_ENST00000510444|lincRNA

---

Grc38\_ENST00000510767|processed\_transcript

---

Grc38\_ENST00000510879|lincRNA

---

Grc38\_ENST00000510907|lincRNA

---

Grc38\_ENST00000511014|lincRNA

---

Grc38\_ENST00000511219|lincRNA

---

Grc38\_ENST00000511602|lincRNA

---

Grc38\_ENST00000511677|lincRNA

---

Grc38\_ENST00000511699|processed\_transcript

---

Grc38\_ENST00000511893|sense\_overlapping

---

Grc38\_ENST00000512241|lincRNA

---

Grc38\_ENST00000512519|lincRNA

---

Grc38\_ENST00000512559|lincRNA

---

Grc38\_ENST00000512652|lincRNA

---

Grc38\_ENST00000512693|lincRNA

---

Grc38\_ENST00000512873|lincRNA

---

Grc38\_ENST00000513207|lincRNA

---

Grc38\_ENST00000513393|lincRNA

---

Grc38\_ENST00000513626|lincRNA

---

Grc38\_ENST00000513875|lincRNA

---

Grc38\_ENST00000513955|lincRNA

---

Grc38\_ENST00000514298|processed\_transcript

---

Grc38\_ENST00000514532|lincRNA

---

Grc38\_ENST00000514568|lincRNA

---

Grc38\_ENST00000514726|lincRNA

---

Grc38\_ENST00000514752|processed\_transcript

---

Grc38\_ENST00000514823|lincRNA

---

Grc38\_ENST00000515343|lincRNA

---

Grc38\_ENST00000515416|lincRNA

---

Grc38\_ENST00000515614|lincRNA

---

Grc38\_ENST00000515670|lincRNA

---

Grc38\_ENST00000515916|miRNA

---

Grc38\_ENST00000515939|snRNA

---

Grc38\_ENST00000515945|snRNA

---

Grc38\_ENST00000515969|snoRNA

---

Grc38\_ENST00000516006|snoRNA

---

Grc38\_ENST00000516050|snRNA

---

Grc38\_ENST00000516053|miRNA

---

Grc38\_ENST00000516077|snRNA

---

Grc38\_ENST00000516078|snRNA

---

Grc38\_ENST00000516087|snoRNA

---

Grc38\_ENST00000516131|snoRNA

---

Grc38\_ENST00000516162|snRNA

---

Grc38\_ENST00000516165|snoRNA

---

Grc38\_ENST00000516180|miRNA

---

Grc38\_ENST00000516191|snoRNA

---

Grc38\_ENST00000516217|snRNA

---

Grc38\_ENST00000516218|snRNA

---

Grc38\_ENST00000516231|snoRNA

---

Grc38\_ENST00000516236|snoRNA

---

Grc38\_ENST00000516254|snRNA

---

Grc38\_ENST00000516272|snRNA

---

Grc38\_ENST00000516316|snRNA

---

Grc38\_ENST00000516339|snRNA

---

Grc38\_ENST00000516375|snRNA

---

Grc38\_ENST00000516404|snoRNA

---

Grc38\_ENST00000516446|snRNA

---

Grc38\_ENST00000516449|snoRNA

---

Grc38\_ENST00000516468|snoRNA

---

Grc38\_ENST00000516481|snoRNA

---

Grc38\_ENST00000516488|snRNA

---

Grc38\_ENST00000516517|snoRNA

---

Grc38\_ENST00000516518|snRNA

---

Grc38\_ENST00000516523|snRNA

---

Grc38\_ENST00000516531|miRNA

---

Grc38\_ENST00000516540|snoRNA

---

Grc38\_ENST00000516543|snoRNA

---

Grc38\_ENST00000516574|snRNA

---

Grc38\_ENST00000516585|snRNA

---

Grc38\_ENST00000516612|snRNA

---

Grc38\_ENST00000516652|snoRNA

---

Grc38\_ENST00000516708|snoRNA

---

Grc38\_ENST00000516710|snRNA

---

Grc38\_ENST00000516712|snRNA

---

Grc38\_ENST00000516736|snRNA

---

Grc38\_ENST00000516747|snRNA

---

Grc38\_ENST00000516752|snRNA

---

Grc38\_ENST00000516795|snRNA

---

Grc38\_ENST00000516810|snRNA

---

Grc38\_ENST00000516830|snRNA

---

Grc38\_ENST00000516842|snRNA

---

Grc38\_ENST00000516869|ribozyme

---

Grc38\_ENST00000516873|snoRNA

---

Grc38\_ENST00000516967|snRNA

---

Grc38\_ENST00000516995|snRNA

---

Grc38\_ENST00000517002|miRNA

---

Grc38\_ENST00000517026|scaRNA

---

Grc38\_ENST00000517051|snRNA

---

Grc38\_ENST00000517121|snRNA

---

Grc38\_ENST00000517162|snRNA

---

Grc38\_ENST00000517181|snRNA

---

Grc38\_ENST00000517186|snRNA

---

Grc38\_ENST00000517199|miRNA

---

Grc38\_ENST00000517281|snoRNA

---

Grc38\_ENST00000517285|snoRNA

---

Grc38\_ENST00000517288|snRNA

---

Grc38\_ENST00000517290|snRNA

---

Grc38\_ENST00000517681|lincRNA

---

Grc38\_ENST00000517739|lincRNA

---

Grc38\_ENST00000517848|lincRNA

---

Grc38\_ENST00000518014|lincRNA

---

Grc38\_ENST00000518103|lincRNA

---

Grc38\_ENST00000518190|lincRNA

---

Grc38\_ENST00000519366|lincRNA

---

Grc38\_ENST00000519550|lincRNA

---

Grc38\_ENST00000519692|processed\_transcript

---

Grc38\_ENST00000519840|lincRNA

---

Grc38\_ENST00000519990|lincRNA

---

Grc38\_ENST00000520259|lincRNA

---

Grc38\_ENST00000520515|processed\_transcript

---

Grc38\_ENST00000520619|processed\_transcript

---

Grc38\_ENST00000520815|sense\_intronic

---

Grc38\_ENST00000520902|lincRNA

---

Grc38\_ENST00000520944|processed\_transcript

---

Grc38\_ENST00000521051|lincRNA

---

Grc38\_ENST00000521074|lincRNA

---

Grc38\_ENST00000521128|lincRNA

---

Grc38\_ENST00000521218|lincRNA

---

Grc38\_ENST00000521369|lincRNA

---

Grc38\_ENST00000521908|lincRNA

---

Grc38\_ENST00000522158|lincRNA

---

Grc38\_ENST00000522547|sense\_intronic

---

Grc38\_ENST00000522581|snRNA

---

Grc38\_ENST00000522604|lincRNA

---

Grc38\_ENST00000522771|lincRNA

---

Grc38\_ENST00000523103|lincRNA

---

Grc38\_ENST00000523671|lincRNA

---

Grc38\_ENST00000523792|lincRNA

---

Grc38\_ENST00000523871|lincRNA

---

Grc38\_ENST00000524287|lincRNA

---

Grc38\_ENST00000524348|sense\_intronic

---

Grc38\_ENST00000524376|sense\_overlapping

---

Grc38\_ENST00000525043|lincRNA

---

Grc38\_ENST00000525556|lincRNA

---

Grc38\_ENST00000525757|lincRNA

---

Grc38\_ENST00000526036|lincRNA

---

Grc38\_ENST00000526091|lincRNA

---

Grc38\_ENST00000526131|lincRNA

---

Grc38\_ENST00000526470|lincRNA

---

Grc38\_ENST00000527970|lincRNA

---

Grc38\_ENST00000528133|sense\_overlapping

---

Grc38\_ENST00000528233|sense\_intronic

---

Grc38\_ENST00000528804|processed\_transcript

---

Grc38\_ENST00000528986|sense\_overlapping

---

Grc38\_ENST00000529089|processed\_transcript

---

Grc38\_ENST00000529743|lincRNA

---

Grc38\_ENST00000529823|sense\_overlapping

---

Grc38\_ENST00000529841|lincRNA

---

Grc38\_ENST00000529902|lincRNA

---

Grc38\_ENST00000530000|lincRNA

---

Grc38\_ENST00000530079|processed\_transcript

---

Grc38\_ENST00000530595|sense\_overlapping

---

Grc38\_ENST00000531381|sense\_overlapping

---

Grc38\_ENST00000531559|lincRNA

---

Grc38\_ENST00000531609|lincRNA

---

Grc38\_ENST00000531894|processed\_transcript

---

Grc38\_ENST00000532109|lincRNA

---

Grc38\_ENST00000532768|lincRNA

---

Grc38\_ENST00000533004|lincRNA

---

Grc38\_ENST00000533615|lincRNA

---

Grc38\_ENST00000533736|lincRNA

---

Grc38\_ENST00000533812|lincRNA

---

Grc38\_ENST00000533859|sense\_intronic

---

Grc38\_ENST00000534593|lincRNA

---

Grc38\_ENST00000534909|lincRNA

---

Grc38\_ENST00000535487|lincRNA

---

Grc38\_ENST00000535689|processed\_transcript

---

Grc38\_ENST00000536898|lincRNA

---

Grc38\_ENST00000537616|sense\_intronic

---

Grc38\_ENST00000537827|lincRNA

---

Grc38\_ENST00000537961|lincRNA

---

Grc38\_ENST00000538077|lincRNA

---

Grc38\_ENST00000539163|processed\_transcript

---

Grc38\_ENST00000539813|lincRNA

---

Grc38\_ENST00000540866|processed\_transcript

---

Grc38\_ENST00000542089|lincRNA

---

Grc38\_ENST00000542475|lincRNA

---

Grc38\_ENST00000543308|lincRNA

---

Grc38\_ENST00000543334|lincRNA

---

Grc38\_ENST00000545177|lincRNA

---

Grc38\_ENST00000545709|sense\_intronic

---

Grc38\_ENST00000546682|processed\_transcript

---

Grc38\_ENST00000546696|lincRNA

---

Grc38\_ENST00000546835|lincRNA

---

Grc38\_ENST00000547033|lincRNA

---

Grc38\_ENST00000547179|lincRNA

---

Grc38\_ENST00000547804|lincRNA

---

Grc38\_ENST00000547876|lincRNA

---

Grc38\_ENST00000548144|processed\_transcript

---

Grc38\_ENST00000548801|sense\_intronic

---

Grc38\_ENST00000549262|lincRNA

---

Grc38\_ENST00000549303|sense\_intronic

---

Grc38\_ENST00000549734|lincRNA

---

Grc38\_ENST00000549796|lincRNA

---

Grc38\_ENST00000550135|processed\_transcript

---

Grc38\_ENST00000550231|processed\_transcript

---

Grc38\_ENST00000550279|lincRNA

---

Grc38\_ENST00000550290|lincRNA

---

Grc38\_ENST00000552015|lincRNA

---

Grc38\_ENST00000552061|sense\_intronic

---

Grc38\_ENST00000552334|lincRNA

---

Grc38\_ENST00000553176|sense\_overlapping

---

Grc38\_ENST00000553181|lincRNA

---

Grc38\_ENST00000553312|sense\_overlapping

---

Grc38\_ENST00000553344|lincRNA

---

Grc38\_ENST00000553378|lincRNA

---

Grc38\_ENST00000553648|lincRNA

---

Grc38\_ENST00000553704|lincRNA

---

Grc38\_ENST00000554129|lincRNA

---

Grc38\_ENST00000554133|processed\_transcript

---

Grc38\_ENST00000554519|lincRNA

---

Grc38\_ENST00000554753|lincRNA

---

Grc38\_ENST00000555227|processed\_transcript

---

Grc38\_ENST00000555282|lincRNA

---

Grc38\_ENST00000555396|lincRNA

---

Grc38\_ENST00000555520|processed\_transcript

---

Grc38\_ENST00000555539|lincRNA

---

Grc38\_ENST00000555871|lincRNA

---

Grc38\_ENST00000555937|processed\_transcript

---

Grc38\_ENST00000555985|lincRNA

---

Grc38\_ENST00000556053|lincRNA

---

Grc38\_ENST00000556072|lincRNA

---

Grc38\_ENST00000556355|processed\_transcript

---

Grc38\_ENST00000556405|lincRNA

---

Grc38\_ENST00000556586|lincRNA

---

Grc38\_ENST00000556720|lincRNA

---

Grc38\_ENST00000556728|lincRNA

---

Grc38\_ENST00000556738|lincRNA

---

Grc38\_ENST00000556895|processed\_transcript

---

Grc38\_ENST00000556899|lincRNA

---

Grc38\_ENST00000557067|lincRNA

---

Grc38\_ENST00000557359|lincRNA

---

Grc38\_ENST00000557481|lincRNA

---

Grc38\_ENST00000557526|lincRNA

---

Grc38\_ENST00000557528|lincRNA

---

Grc38\_ENST00000557682|processed\_transcript

---

Grc38\_ENST00000557800|sense\_intronic

---

Grc38\_ENST00000557910|lincRNA

---

Grc38\_ENST00000558085|lincRNA

---

Grc38\_ENST00000558097|lincRNA

---

Grc38\_ENST00000558277|lincRNA

---

Grc38\_ENST00000558675|lincRNA

---

Grc38\_ENST00000558963|lincRNA

---

Grc38\_ENST00000559008|lincRNA

---

Grc38\_ENST00000559458|3prime\_overlapping\_ncrna

---

Grc38\_ENST00000559825|lincRNA

---

Grc38\_ENST00000559985|lincRNA

---

Grc38\_ENST00000560049|lincRNA

---

Grc38\_ENST00000560153|lincRNA

---

Grc38\_ENST00000560378|sense\_intronic

---

Grc38\_ENST00000560400|sense\_overlapping

---

Grc38\_ENST00000560705|lincRNA

---

Grc38\_ENST00000560732|lincRNA

---

Grc38\_ENST00000560750|lincRNA

---

Grc38\_ENST00000561054|lincRNA

---

Grc38\_ENST00000561232|lincRNA

---

Grc38\_ENST00000561358|lincRNA

---

Grc38\_ENST00000561473|lincRNA

---

Grc38\_ENST00000561507|sense\_overlapping

---

Grc38\_ENST00000561521|lincRNA

---

Grc38\_ENST00000561542|sense\_overlapping

---

Grc38\_ENST00000561587|lincRNA

---

Grc38\_ENST00000561624|sense\_intronic

---

Grc38\_ENST00000561652|sense\_overlapping

---

Grc38\_ENST00000561746|lincRNA

---

Grc38\_ENST00000561788|processed\_transcript

---

Grc38\_ENST00000561847|sense\_intronic

---

Grc38\_ENST00000561926|lincRNA

---

Grc38\_ENST00000561961|sense\_overlapping

---

Grc38\_ENST00000561978|lincRNA

---

Grc38\_ENST00000562082|sense\_overlapping

---

Grc38\_ENST00000562167|lincRNA

---

Grc38\_ENST00000562182|lincRNA

---

Grc38\_ENST00000562242|sense\_overlapping

---

Grc38\_ENST00000562262|sense\_intronic

---

Grc38\_ENST00000562328|sense\_overlapping

---

Grc38\_ENST00000562466|lincRNA

---

Grc38\_ENST00000562471|lincRNA

---

Grc38\_ENST00000562493|lincRNA

---

Grc38\_ENST00000562582|lincRNA

---

Grc38\_ENST00000562624|lincRNA

---

Grc38\_ENST00000562644|sense\_intronic

---

Grc38\_ENST00000562795|lincRNA

---

Grc38\_ENST00000562796|lincRNA

---

Grc38\_ENST00000562894|sense\_intronic

---

Grc38\_ENST00000562904|lincRNA

---

Grc38\_ENST00000562921|lincRNA

---

Grc38\_ENST00000563079|lincRNA

---

Grc38\_ENST00000563103|sense\_intronic

---

Grc38\_ENST00000563129|3prime\_overlapping\_ncrna

---

Grc38\_ENST00000563225|lincRNA

---

Grc38\_ENST00000563357|lincRNA

---

Grc38\_ENST00000563417|lincRNA

---

Grc38\_ENST00000563424|lincRNA

---

Grc38\_ENST00000563495|sense\_intronic

---

Grc38\_ENST00000563631|lincRNA

---

Grc38\_ENST00000563635|processed\_transcript

---

Grc38\_ENST00000563701|lincRNA

---

Grc38\_ENST00000563727|lincRNA

---

Grc38\_ENST00000564014|lincRNA

---

Grc38\_ENST00000564121|lincRNA

---

Grc38\_ENST00000564237|sense\_overlapping

---

Grc38\_ENST00000564305|lincRNA

---

Grc38\_ENST00000564352|sense\_overlapping

---

Grc38\_ENST00000564363|lincRNA

---

Grc38\_ENST00000564401|lincRNA

---

Grc38\_ENST00000564402|lincRNA

---

Grc38\_ENST00000564460|sense\_intronic

---

Grc38\_ENST00000564536|processed\_transcript

---

Grc38\_ENST00000564585|lincRNA

---

Grc38\_ENST00000564593|lincRNA

---

Grc38\_ENST00000564619|lincRNA

---

Grc38\_ENST00000564623|sense\_overlapping

---

Grc38\_ENST00000564681|lincRNA

---

Grc38\_ENST00000564771|sense\_overlapping

---

Grc38\_ENST00000564834|lincRNA

---

Grc38\_ENST00000564956|sense\_overlapping

---

Grc38\_ENST00000565008|lincRNA

---

Grc38\_ENST00000565058|sense\_intronic

---

Grc38\_ENST00000565082|lincRNA

---

Grc38\_ENST00000565113|processed\_transcript

---

Grc38\_ENST00000565189|lincRNA

---

Grc38\_ENST00000565336|lincRNA

---

Grc38\_ENST00000565493|lincRNA

---

Grc38\_ENST00000565521|processed\_transcript

---

Grc38\_ENST00000565549|lincRNA

---

Grc38\_ENST00000565617|lincRNA

---

Grc38\_ENST00000566193|lincRNA

---

Grc38\_ENST00000566225|lincRNA

---

Grc38\_ENST00000566291|sense\_overlapping

---

Grc38\_ENST00000566293|sense\_overlapping

---

Grc38\_ENST00000566385|lincRNA

---

Grc38\_ENST00000566440|lincRNA

---

Grc38\_ENST00000566551|lincRNA

---

Grc38\_ENST00000566639|sense\_intronic

---

Grc38\_ENST00000566733|lincRNA

---

Grc38\_ENST00000566747|sense\_overlapping

---

Grc38\_ENST00000566929|lincRNA

---

Grc38\_ENST00000566954|sense\_overlapping

---

Grc38\_ENST00000566996|3prime\_overlapping\_ncrna

---

Grc38\_ENST00000567058|lincRNA

---

Grc38\_ENST00000567102|sense\_overlapping

---

Grc38\_ENST00000567359|sense\_intronic

---

Grc38\_ENST00000567465|lincRNA

---

Grc38\_ENST00000567540|lincRNA

---

Grc38\_ENST00000567714|sense\_overlapping

---

Grc38\_ENST00000567718|lincRNA

---

Grc38\_ENST00000567795|sense\_intronic

---

Grc38\_ENST00000567801|lincRNA

---

Grc38\_ENST00000567834|3prime\_overlapping\_ncrna

---

Grc38\_ENST00000567904|lincRNA

---

Grc38\_ENST00000567953|sense\_intronic

---

Grc38\_ENST00000568031|lincRNA

---

Grc38\_ENST00000568063|lincRNA

---

Grc38\_ENST00000568144|sense\_intronic

---

Grc38\_ENST00000568248|lincRNA

---

Grc38\_ENST00000568262|sense\_intronic

---

Grc38\_ENST00000568279|lincRNA

---

Grc38\_ENST00000568314|sense\_intronic

---

Grc38\_ENST00000568389|lincRNA

---

Grc38\_ENST00000568395|processed\_transcript

---

Grc38\_ENST00000568418|lincRNA

---

Grc38\_ENST00000568421|sense\_overlapping

---

Grc38\_ENST00000568479|sense\_overlapping

---

Grc38\_ENST00000568587|lincRNA

---

Grc38\_ENST00000568695|lincRNA

---

Grc38\_ENST00000568911|lincRNA

---

Grc38\_ENST00000568966|sense\_overlapping

---

Grc38\_ENST00000569008|lincRNA

---

Grc38\_ENST00000569034|sense\_overlapping

---

Grc38\_ENST00000569048|lincRNA

---

Grc38\_ENST00000569087|lincRNA

---

Grc38\_ENST00000569104|lincRNA

---

Grc38\_ENST00000569134|lincRNA

---

Grc38\_ENST00000569215|lincRNA

---

Grc38\_ENST00000569275|lincRNA

---

Grc38\_ENST00000569381|lincRNA

---

Grc38\_ENST00000569425|lincRNA

---

Grc38\_ENST00000569460|lincRNA

---

Grc38\_ENST00000569476|lincRNA

---

Grc38\_ENST00000569621|lincRNA

---

Grc38\_ENST00000569736|lincRNA

---

Grc38\_ENST00000569833|lincRNA

---

Grc38\_ENST00000569835|lincRNA

---

Grc38\_ENST00000569908|lincRNA

---

Grc38\_ENST00000569913|lincRNA

---

Grc38\_ENST00000569932|lincRNA

---

Grc38\_ENST00000569966|lincRNA

---

Grc38\_ENST00000569969|processed\_transcript

---

Grc38\_ENST00000569998|lincRNA

---

Grc38\_ENST00000570073|sense\_intronic

---

Grc38\_ENST00000570082|lincRNA

---

Grc38\_ENST00000570130|lincRNA

---

Grc38\_ENST00000570140|sense\_overlapping

---

Grc38\_ENST00000570269|lincRNA

---

Grc38\_ENST00000570416|lincRNA

---

Grc38\_ENST00000570809|lincRNA

---

Grc38\_ENST00000571152|lincRNA

---

Grc38\_ENST00000571302|sense\_overlapping

---

Grc38\_ENST00000571595|lincRNA

---

Grc38\_ENST00000571619|lincRNA

---

Grc38\_ENST00000572165|processed\_transcript

---

Grc38\_ENST00000572520|miRNA

---

Grc38\_ENST00000572849|snRNA

---

Grc38\_ENST00000572877|sense\_intronic

---

Grc38\_ENST00000573042|lincRNA

---

Grc38\_ENST00000573063|processed\_transcript

---

Grc38\_ENST00000573075|lincRNA

---

Grc38\_ENST00000573127|lincRNA

---

Grc38\_ENST00000573167|lincRNA

---

Grc38\_ENST00000574016|lincRNA

---

Grc38\_ENST00000574306|lincRNA

---

Grc38\_ENST00000574526|lincRNA

---

Grc38\_ENST00000574724|processed\_transcript

---

Grc38\_ENST00000574861|snRNA

---

Grc38\_ENST00000575305|processed\_transcript

---

Grc38\_ENST00000575331|processed\_transcript

---

Grc38\_ENST00000575446|lincRNA

---

Grc38\_ENST00000575743|processed\_transcript

---

Grc38\_ENST00000576365|lincRNA

---

Grc38\_ENST00000576489|lincRNA

---

Grc38\_ENST00000576738|lincRNA

---

Grc38\_ENST00000576749|lincRNA

---

Grc38\_ENST00000576963|lincRNA

---

Grc38\_ENST00000577066|lincRNA

---

Grc38\_ENST00000577087|lincRNA

---

Grc38\_ENST00000577254|miRNA

---

Grc38\_ENST00000577267|lincRNA

---

Grc38\_ENST00000577309|lincRNA

---

Grc38\_ENST00000577313|miRNA

---

Grc38\_ENST00000577346|lincRNA

---

Grc38\_ENST00000577413|miRNA

---

Grc38\_ENST00000577439|lincRNA

---

Grc38\_ENST00000577639|miRNA

---

Grc38\_ENST00000577864|miRNA

---

Grc38\_ENST00000577877|miRNA

---

Grc38\_ENST00000577895|miRNA

---

Grc38\_ENST00000577912|miRNA

---

Grc38\_ENST00000577937|miRNA

---

Grc38\_ENST00000577956|lincRNA

---

Grc38\_ENST00000578012|miRNA

---

Grc38\_ENST00000578032|miRNA

---

Grc38\_ENST00000578083|miRNA

---

Grc38\_ENST00000578179|snoRNA

---

Grc38\_ENST00000578214|sense\_intronic

---

Grc38\_ENST00000578216|sense\_intronic

---

Grc38\_ENST00000578271|miRNA

---

Grc38\_ENST00000578365|miRNA

---

Grc38\_ENST00000578394|miRNA

---

Grc38\_ENST00000578418|miRNA

---

Grc38\_ENST00000578427|lincRNA

---

Grc38\_ENST00000578492|processed\_transcript

---

Grc38\_ENST00000578539|sense\_intronic

---

Grc38\_ENST00000578628|miRNA

---

Grc38\_ENST00000578669|miRNA

---

Grc38\_ENST00000578728|miRNA

---

Grc38\_ENST00000578762|miRNA

---

Grc38\_ENST00000578899|processed\_transcript

---

Grc38\_ENST00000579049|sense\_intronic

---

Grc38\_ENST00000579057|lincRNA

---

Grc38\_ENST00000579126|processed\_transcript

---

Grc38\_ENST00000579148|miRNA

---

Grc38\_ENST00000579174|processed\_transcript

---

Grc38\_ENST00000579188|sense\_intronic

---

Grc38\_ENST00000579240|miRNA

---

Grc38\_ENST00000579259|miRNA

---

Grc38\_ENST00000579300|miRNA

---

Grc38\_ENST00000579427|miRNA

---

Grc38\_ENST00000579458|lincRNA

---

Grc38\_ENST00000579518|miRNA

---

Grc38\_ENST00000579524|miRNA

---

Grc38\_ENST00000579548|miRNA

---

Grc38\_ENST00000579607|miRNA

---

Grc38\_ENST00000579736|miRNA

---

Grc38\_ENST00000579781|lincRNA

---

Grc38\_ENST00000579833|lincRNA

---

Grc38\_ENST00000579867|miRNA

---

Grc38\_ENST00000579931|miRNA

---

Grc38\_ENST00000580134|lincRNA

---

Grc38\_ENST00000580154|miRNA

---

Grc38\_ENST00000580180|processed\_transcript

---

Grc38\_ENST00000580185|miRNA

---

Grc38\_ENST00000580197|lincRNA

---

Grc38\_ENST00000580268|miRNA

---

Grc38\_ENST00000580282|lincRNA

---

Grc38\_ENST00000580305|miRNA

---

Grc38\_ENST00000580516|miRNA

---

Grc38\_ENST00000580563|miRNA

---

Grc38\_ENST00000580600|miRNA

---

Grc38\_ENST00000580609|miRNA

---

Grc38\_ENST00000580651|miRNA

---

Grc38\_ENST00000580653|miRNA

---

Grc38\_ENST00000580659|lincRNA

---

Grc38\_ENST00000580703|miRNA

---

Grc38\_ENST00000580730|miRNA

---

Grc38\_ENST00000580767|miRNA

---

Grc38\_ENST00000580848|miRNA

---

Grc38\_ENST00000580891|sense\_intronic

---

Grc38\_ENST00000580935|miRNA

---

Grc38\_ENST00000580975|sense\_intronic

---

Grc38\_ENST00000580979|sense\_intronic

---

Grc38\_ENST00000581011|processed\_transcript

---

Grc38\_ENST00000581095|miRNA

---

Grc38\_ENST00000581102|miRNA

---

Grc38\_ENST00000581119|miRNA

---

Grc38\_ENST00000581149|miRNA

---

Grc38\_ENST00000581340|miRNA

---

Grc38\_ENST00000581489|lincRNA

---

Grc38\_ENST00000581535|miRNA

---

Grc38\_ENST00000581549|lincRNA

---

Grc38\_ENST00000581650|miRNA

---

Grc38\_ENST00000581704|miRNA

---

Grc38\_ENST00000581712|sense\_intronic

---

Grc38\_ENST00000581719|processed\_transcript

---

Grc38\_ENST00000581730|miRNA

---

Grc38\_ENST00000581789|miRNA

---

Grc38\_ENST00000581794|processed\_transcript

---

Grc38\_ENST00000581837|miRNA

---

Grc38\_ENST00000581862|processed\_transcript

---

Grc38\_ENST00000581881|miRNA

---

Grc38\_ENST00000581907|miRNA

---

Grc38\_ENST00000581913|processed\_transcript

---

Grc38\_ENST00000581915|processed\_transcript

---

Grc38\_ENST00000581999|miRNA

---

Grc38\_ENST00000582000|miRNA

---

Grc38\_ENST00000582069|miRNA

---

Grc38\_ENST00000582080|sense\_intronic

---

Grc38\_ENST00000582102|miRNA

---

Grc38\_ENST00000582159|miRNA

---

Grc38\_ENST00000582251|lincRNA

---

Grc38\_ENST00000582284|miRNA

---

Grc38\_ENST00000582320|lincRNA

---

Grc38\_ENST00000582362|miRNA

---

Grc38\_ENST00000582396|miRNA

---

Grc38\_ENST00000582403|miRNA

---

Grc38\_ENST00000582442|miRNA

---

Grc38\_ENST00000582452|lincRNA

---

Grc38\_ENST00000582495|miRNA

---

Grc38\_ENST00000582550|snoRNA

---

Grc38\_ENST00000582628|miRNA

---

Grc38\_ENST00000582639|miRNA

---

Grc38\_ENST00000582720|miRNA

---

Grc38\_ENST00000582739|miRNA

---

Grc38\_ENST00000582777|miRNA

---

Grc38\_ENST00000582803|miRNA

---

Grc38\_ENST00000582816|miRNA

---

Grc38\_ENST00000582846|miRNA

---

Grc38\_ENST00000582866|lincRNA

---

Grc38\_ENST00000582911|processed\_transcript

---

Grc38\_ENST00000582983|lincRNA

---

Grc38\_ENST00000583157|miRNA

---

Grc38\_ENST00000583195|lincRNA

---

Grc38\_ENST00000583253|sense\_intronic

---

Grc38\_ENST00000583272|miRNA

---

Grc38\_ENST00000583311|miRNA

---

Grc38\_ENST00000583386|miRNA

---

Grc38\_ENST00000583416|lincRNA

---

Grc38\_ENST00000583429|miRNA

---

Grc38\_ENST00000583431|miRNA

---

Grc38\_ENST00000583516|sense\_intronic

---

Grc38\_ENST00000583573|miRNA

---

Grc38\_ENST00000583596|miRNA

---

Grc38\_ENST00000583779|miRNA

---

Grc38\_ENST00000583845|miRNA

---

Grc38\_ENST00000583893|miRNA

---

Grc38\_ENST00000583964|miRNA

---

Grc38\_ENST00000584085|miRNA

---

Grc38\_ENST00000584092|miRNA

---

Grc38\_ENST00000584133|miRNA

---

Grc38\_ENST00000584136|miRNA

---

Grc38\_ENST00000584172|miRNA

---

Grc38\_ENST00000584173|miRNA

---

Grc38\_ENST00000584200|miRNA

---

Grc38\_ENST00000584249|miRNA

---

Grc38\_ENST00000584254|miRNA

---

Grc38\_ENST00000584360|miRNA

---

Grc38\_ENST00000584382|sense\_intronic

---

Grc38\_ENST00000584545|miRNA

---

Grc38\_ENST00000584571|miRNA

---

Grc38\_ENST00000584572|miRNA

---

Grc38\_ENST00000584629|miRNA

---

Grc38\_ENST00000584664|miRNA

---

Grc38\_ENST00000584683|lincRNA

---

Grc38\_ENST00000584721|lincRNA

---

Grc38\_ENST00000584805|lincRNA

---

Grc38\_ENST00000584839|miRNA

---

Grc38\_ENST00000584882|miRNA

---

Grc38\_ENST00000584926|processed\_transcript

---

Grc38\_ENST00000584930|miRNA

---

Grc38\_ENST00000585065|lincRNA

---

Grc38\_ENST00000585112|miRNA

---

Grc38\_ENST00000585238|miRNA

---

Grc38\_ENST00000585445|lincRNA

---

Grc38\_ENST00000585484|lincRNA

---

Grc38\_ENST00000585627|lincRNA

---

Grc38\_ENST00000585776|lincRNA

---

Grc38\_ENST00000585877|sense\_intronic

---

Grc38\_ENST00000586064|processed\_transcript

---

Grc38\_ENST00000586209|lincRNA

---

Grc38\_ENST00000586645|lincRNA

---

Grc38\_ENST00000586726|processed\_transcript

---

Grc38\_ENST00000586798|processed\_transcript

---

Grc38\_ENST00000586885|lincRNA

---

Grc38\_ENST00000586905|lincRNA

---

Grc38\_ENST00000586922|lincRNA

---

Grc38\_ENST00000586943|processed\_transcript

---

Grc38\_ENST00000586954|lincRNA

---

Grc38\_ENST00000586983|sense\_intronic

---

Grc38\_ENST00000587125|lincRNA

---

Grc38\_ENST00000587128|lincRNA

---

Grc38\_ENST00000587616|lincRNA

---

Grc38\_ENST00000587933|sense\_intronic

---

Grc38\_ENST00000587960|lincRNA

---

Grc38\_ENST00000587961|lincRNA

---

Grc38\_ENST00000588275|lincRNA

---

Grc38\_ENST00000588341|lincRNA

---

Grc38\_ENST00000588424|lincRNA

---

Grc38\_ENST00000588438|lincRNA

---

Grc38\_ENST00000588495|lincRNA

---

Grc38\_ENST00000588548|lincRNA

---

Grc38\_ENST00000589187|processed\_transcript

---

Grc38\_ENST00000589217|processed\_transcript

---

Grc38\_ENST00000589259|lincRNA

---

Grc38\_ENST00000589716|lincRNA

---

Grc38\_ENST00000589740|lincRNA

---

Grc38\_ENST00000589754|lincRNA

---

Grc38\_ENST00000590086|lincRNA

---

Grc38\_ENST00000590241|sense\_intronic

---

Grc38\_ENST00000590274|lincRNA

---

Grc38\_ENST00000590353|processed\_transcript

---

Grc38\_ENST00000590491|sense\_intronic

---

Grc38\_ENST00000590505|processed\_transcript

---

Grc38\_ENST00000590622|lincRNA

---

Grc38\_ENST00000590628|lincRNA

---

Grc38\_ENST00000590740|lincRNA

---

Grc38\_ENST00000590750|lincRNA

---

Grc38\_ENST00000590797|lincRNA

---

Grc38\_ENST00000591222|lincRNA

---

Grc38\_ENST00000591343|sense\_intronic

---

Grc38\_ENST00000591372|lincRNA

---

Grc38\_ENST00000591702|lincRNA

---

Grc38\_ENST00000591892|processed\_transcript

---

Grc38\_ENST00000592107|lincRNA

---

Grc38\_ENST00000592135|lincRNA

---

Grc38\_ENST00000592187|processed\_transcript

---

Grc38\_ENST00000592371|lincRNA

---

Grc38\_ENST00000592381|lincRNA

---

Grc38\_ENST00000592572|lincRNA

---

Grc38\_ENST00000592638|lincRNA

---

Grc38\_ENST00000592670|lincRNA

---

Grc38\_ENST00000592718|lincRNA

---

Grc38\_ENST00000592744|lincRNA

---

Grc38\_ENST00000592918|lincRNA

---

Grc38\_ENST00000592939|processed\_transcript

---

Grc38\_ENST00000593117|lincRNA

---

Grc38\_ENST00000593234|lincRNA

---

Grc38\_ENST00000593393|lincRNA

---

Grc38\_ENST00000593427|lincRNA

---

Grc38\_ENST00000593604|lincRNA

---

Grc38\_ENST00000593779|sense\_intronic

---

Grc38\_ENST00000594086|lincRNA

---

Grc38\_ENST00000594119|processed\_transcript

---

Grc38\_ENST00000594329|lincRNA

---

Grc38\_ENST00000594413|lincRNA

---

Grc38\_ENST00000594418|lincRNA

---

Grc38\_ENST00000594816|lincRNA

---

Grc38\_ENST00000594934|lincRNA

---

Grc38\_ENST00000595059|lincRNA

---

Grc38\_ENST00000595094|lincRNA

---

Grc38\_ENST00000595310|lincRNA

---

Grc38\_ENST00000595508|processed\_transcript

---

Grc38\_ENST00000595886|lincRNA

---

Grc38\_ENST00000595909|lincRNA

---

Grc38\_ENST00000596000|lincRNA

---

Grc38\_ENST00000596203|lincRNA

---

Grc38\_ENST00000596283|lincRNA

---

Grc38\_ENST00000596769|lincRNA

---

Grc38\_ENST00000597173|lincRNA

---

Grc38\_ENST00000597260|sense\_intronic

---

Grc38\_ENST00000597267|processed\_transcript

---

Grc38\_ENST00000597488|processed\_transcript

---

Grc38\_ENST00000597502|processed\_transcript

---

Grc38\_ENST00000597651|sense\_overlapping

---

Grc38\_ENST00000598112|sense\_intronic

---

Grc38\_ENST00000598322|sense\_intronic

---

Grc38\_ENST00000598368|processed\_transcript

---

Grc38\_ENST00000598378|processed\_transcript

---

Grc38\_ENST00000598846|processed\_transcript

---

Grc38\_ENST00000598988|sense\_intronic

---

Grc38\_ENST00000598996|lincRNA

---

Grc38\_ENST00000599109|processed\_transcript

---

Grc38\_ENST00000599320|lincRNA

---

Grc38\_ENST00000599411|lincRNA

---

Grc38\_ENST00000599486|lincRNA

---

Grc38\_ENST00000599640|lincRNA

---

Grc38\_ENST00000599749|lincRNA

---

Grc38\_ENST00000600225|processed\_transcript

---

Grc38\_ENST00000600643|lincRNA

---

Grc38\_ENST00000601079|processed\_transcript

---

Grc38\_ENST00000601420|sense\_intronic

---

Grc38\_ENST00000601776|lincRNA

---

Grc38\_ENST00000602145|lincRNA

---

Grc38\_ENST00000602277|lincRNA

---

Grc38\_ENST00000602315|lincRNA

---

Grc38\_ENST00000602324|lincRNA

---

Grc38\_ENST00000602396|lincRNA

---

Grc38\_ENST00000602458|lincRNA

---

Grc38\_ENST00000602461|lincRNA

---

Grc38\_ENST00000602481|sense\_intronic

---

Grc38\_ENST00000602488|processed\_transcript

---

Grc38\_ENST00000602507|lincRNA

---

Grc38\_ENST00000602510|lincRNA

---

Grc38\_ENST00000602543|lincRNA

---

Grc38\_ENST00000602575|sense\_intronic

---

Grc38\_ENST00000602585|lincRNA

---

Grc38\_ENST00000602594|lincRNA

---

Grc38\_ENST00000602605|sense\_intronic

---

Grc38\_ENST00000602652|lincRNA

---

Grc38\_ENST00000602654|lincRNA

---

Grc38\_ENST00000602663|sense\_intronic

---

Grc38\_ENST00000602761|lincRNA

---

Grc38\_ENST00000602772|lincRNA

---

Grc38\_ENST00000602812|lincRNA

---

Grc38\_ENST00000602845|lincRNA

---

Grc38\_ENST00000602847|lincRNA

---

Grc38\_ENST00000602886|sense\_intronic

---

Grc38\_ENST00000602890|lincRNA

---

Grc38\_ENST00000602957|lincRNA

---

Grc38\_ENST00000602964|lincRNA

---

Grc38\_ENST00000602988|sense\_intronic

---

Grc38\_ENST00000603037|sense\_intronic

---

Grc38\_ENST00000603308|sense\_intronic

---

Grc38\_ENST00000603385|lincRNA

---

Grc38\_ENST00000603487|lincRNA

---

Grc38\_ENST00000603633|lincRNA

---

Grc38\_ENST00000604070|sense\_intronic

---

Grc38\_ENST00000604157|lincRNA

---

Grc38\_ENST00000604397|lincRNA

---

Grc38\_ENST00000604411|lincRNA

---

Grc38\_ENST00000604430|lincRNA

---

Grc38\_ENST00000604451|sense\_intronic

---

Grc38\_ENST00000604491|lincRNA

---

Grc38\_ENST00000604514|lincRNA

---

Grc38\_ENST00000604724|lincRNA

---

Grc38\_ENST00000604760|lincRNA

---

Grc38\_ENST00000604855|lincRNA

---

Grc38\_ENST00000604982|lincRNA

---

Grc38\_ENST00000605200|lincRNA

---

Grc38\_ENST00000605249|lincRNA

---

Grc38\_ENST00000605350|lincRNA

---

Grc38\_ENST00000605386|lincRNA

---

Grc38\_ENST00000605741|lincRNA

---

Grc38\_ENST00000605794|processed\_transcript

---

Grc38\_ENST00000605834|lincRNA

---

Grc38\_ENST00000605862|processed\_transcript

---

Grc38\_ENST00000605920|lincRNA

---

Grc38\_ENST00000605929|lincRNA

---

Grc38\_ENST00000605984|lincRNA

---

Grc38\_ENST00000606004|processed\_transcript

---

Grc38\_ENST00000606121|miRNA

---

Grc38\_ENST00000606125|lincRNA

---

Grc38\_ENST00000606197|lincRNA

---

Grc38\_ENST00000606272|lincRNA

---

Grc38\_ENST00000606285|lincRNA

---

Grc38\_ENST00000606336|lincRNA

---

Grc38\_ENST00000606420|snoRNA

---

Grc38\_ENST00000606448|lincRNA

---

Grc38\_ENST00000606512|sense\_intronic

---

Grc38\_ENST00000606534|snRNA

---

Grc38\_ENST00000606574|snRNA

---

Grc38\_ENST00000606640|sense\_intronic

---

Grc38\_ENST00000606686|lincRNA

---

Grc38\_ENST00000606723|lincRNA

---

Grc38\_ENST00000606727|lincRNA

---

Grc38\_ENST00000606743|lincRNA

---

Grc38\_ENST00000606807|miRNA

---

Grc38\_ENST00000606898|lincRNA

---

Grc38\_ENST00000606908|lincRNA

---

Grc38\_ENST00000606927|sense\_intronic

---

Grc38\_ENST00000606966|lincRNA

---

Grc38\_ENST00000607032|lincRNA

---

Grc38\_ENST00000607056|lincRNA

---

Grc38\_ENST00000607061|lincRNA

---

Grc38\_ENST00000607100|lincRNA

---

Grc38\_ENST00000607261|snoRNA

---

Grc38\_ENST00000607307|lincRNA

---

Grc38\_ENST00000607315|lincRNA

---

Grc38\_ENST00000607334|miRNA

---

Grc38\_ENST00000607397|lincRNA

---

Grc38\_ENST00000607417|snoRNA

---

Grc38\_ENST00000607496|sense\_intronic

---

Grc38\_ENST00000607575|miRNA

---

Grc38\_ENST00000607654|lincRNA

---

Grc38\_ENST00000607727|lincRNA

---

Grc38\_ENST00000607728|snRNA

---

Grc38\_ENST00000607746|lincRNA

---

Grc38\_ENST00000607781|lincRNA

---

Grc38\_ENST00000607794|lincRNA

---

Grc38\_ENST00000607836|lincRNA

---

Grc38\_ENST00000607850|processed\_transcript

---

Grc38\_ENST00000607869|miRNA

---

Grc38\_ENST00000608005|lincRNA

---

Grc38\_ENST00000608023|lincRNA

---

Grc38\_ENST00000608028|lincRNA

---

Grc38\_ENST00000608123|lincRNA

---

Grc38\_ENST00000608169|lincRNA

---

Grc38\_ENST00000608206|lincRNA

---

Grc38\_ENST00000608272|processed\_transcript

---

Grc38\_ENST00000608317|lincRNA

---

Grc38\_ENST00000608335|lincRNA

---

Grc38\_ENST00000608358|lincRNA

---

Grc38\_ENST00000608373|lincRNA

---

Grc38\_ENST00000608459|snoRNA

---

Grc38\_ENST00000608477|lincRNA

---

Grc38\_ENST00000608487|processed\_transcript

---

Grc38\_ENST00000608511|processed\_transcript

---

Grc38\_ENST00000608521|processed\_transcript

---

Grc38\_ENST00000608591|lincRNA

---

Grc38\_ENST00000608651|lincRNA

---

Grc38\_ENST00000608694|lincRNA

---

Grc38\_ENST00000608760|lincRNA

---

Grc38\_ENST00000608834|lincRNA

---

Grc38\_ENST00000608925|lincRNA

---

Grc38\_ENST00000608990|lincRNA

---

Grc38\_ENST00000609089|lincRNA

---

Grc38\_ENST00000609127|lincRNA

---

Grc38\_ENST00000609162|lincRNA

---

Grc38\_ENST00000609169|processed\_transcript

---

Grc38\_ENST00000609170|processed\_transcript

---

Grc38\_ENST00000609248|processed\_transcript

---

Grc38\_ENST00000609270|lincRNA

---

Grc38\_ENST00000609352|processed\_transcript

---

Grc38\_ENST00000609398|lincRNA

---

Grc38\_ENST00000609407|lincRNA

---

Grc38\_ENST00000609475|sense\_intronic

---

Grc38\_ENST00000609522|lincRNA

---

Grc38\_ENST00000609581|lincRNA

---

Grc38\_ENST00000609590|processed\_transcript

---

Grc38\_ENST00000609596|lincRNA

---

Grc38\_ENST00000609620|snoRNA

---

Grc38\_ENST00000609687|processed\_transcript

---

Grc38\_ENST00000609708|lincRNA

---

Grc38\_ENST00000609742|lincRNA

---

Grc38\_ENST00000609755|lincRNA

---

Grc38\_ENST00000609801|lincRNA

---

Grc38\_ENST00000609803|lincRNA

---

Grc38\_ENST00000609914|processed\_transcript

---

Grc38\_ENST00000609990|lincRNA

---

Grc38\_ENST00000610034|lincRNA

---

Grc38\_ENST00000610036|lincRNA

---

Grc38\_ENST00000610119|lincRNA

---

Grc38\_ENST00000610128|lincRNA

---

Grc38\_ENST00000610155|processed\_transcript

---

Grc38\_ENST00000610382|miRNA

---

Grc38\_ENST00000610425|miRNA

---

Grc38\_ENST00000610512|miRNA

---

Grc38\_ENST00000610544|miRNA

---

Grc38\_ENST00000610578|lincRNA

---

Grc38\_ENST00000610686|miRNA

---

Grc38\_ENST00000610697|miRNA

---

Grc38\_ENST00000610778|lincRNA

---

Grc38\_ENST00000610848|miRNA

---

Grc38\_ENST00000611043|miRNA

---

Grc38\_ENST00000611182|lincRNA

---

Grc38\_ENST00000611189|sense\_intronic

---

Grc38\_ENST00000611310|miRNA

---

Grc38\_ENST00000611566|sense\_intronic

---

Grc38\_ENST00000611613|miRNA

---

Grc38\_ENST00000611627|lincRNA

---

Grc38\_ENST00000611652|miRNA

---

Grc38\_ENST00000611654|lincRNA

---

Grc38\_ENST00000611708|lincRNA

---

Grc38\_ENST00000611742|sense\_intronic

---

Grc38\_ENST00000611809|sense\_intronic

---

Grc38\_ENST00000611877|lincRNA

---

Grc38\_ENST00000611955|miRNA

---

Grc38\_ENST00000611970|miRNA

---

Grc38\_ENST00000612252|lincRNA

---

Grc38\_ENST00000612268|miRNA

---

Grc38\_ENST00000612438|lincRNA

---

Grc38\_ENST00000612545|lincRNA

---

Grc38\_ENST00000612592|lincRNA

---

Grc38\_ENST00000612598|lincRNA

---

Grc38\_ENST00000612697|miRNA

---

Grc38\_ENST00000612767|miRNA

---

Grc38\_ENST00000612781|lincRNA

---

Grc38\_ENST00000612792|lincRNA

---

Grc38\_ENST00000612900|processed\_transcript

---

Grc38\_ENST00000612936|miRNA

---

Grc38\_ENST00000612970|lincRNA

---

Grc38\_ENST00000612997|lincRNA

---

Grc38\_ENST00000613093|lincRNA

---

Grc38\_ENST00000613141|lincRNA

---

Grc38\_ENST00000613161|lincRNA

---

Grc38\_ENST00000613189|miRNA

---

Grc38\_ENST00000613291|lincRNA

---

Grc38\_ENST00000613361|lincRNA

---

Grc38\_ENST00000613364|miRNA

---

Grc38\_ENST00000613561|miRNA

---

Grc38\_ENST00000613747|miRNA

---

Grc38\_ENST00000613749|lincRNA

---

Grc38\_ENST00000613809|lincRNA

---

Grc38\_ENST00000614046|lincRNA

---

Grc38\_ENST00000614175|miRNA

---

Grc38\_ENST00000614177|lincRNA

---

Grc38\_ENST00000614316|lincRNA

---

Grc38\_ENST00000614334|lincRNA

---

Grc38\_ENST00000614362|processed\_transcript

---

Grc38\_ENST00000614371|miRNA

---

Grc38\_ENST00000614400|lincRNA

---

Grc38\_ENST00000614510|processed\_transcript

---

Grc38\_ENST00000614515|miRNA

---

Grc38\_ENST00000614517|lincRNA

---

Grc38\_ENST00000614576|lincRNA

---

Grc38\_ENST00000614758|miRNA

---

Grc38\_ENST00000614761|miRNA

---

Grc38\_ENST00000614764|miRNA

---

Grc38\_ENST00000614781|sense\_intronic

---

Grc38\_ENST00000614814|miRNA

---

Grc38\_ENST00000614958|miRNA

---

Grc38\_ENST00000615026|miRNA

---

Grc38\_ENST00000615168|lincRNA

---

Grc38\_ENST00000615236|miRNA

---

Grc38\_ENST00000615251|lincRNA

---

Grc38\_ENST00000615314|processed\_transcript

---

Grc38\_ENST00000615338|miRNA

---

Grc38\_ENST00000615348|miRNA

---

Grc38\_ENST00000615351|miRNA

---

Grc38\_ENST00000615357|lincRNA

---

Grc38\_ENST00000615428|miRNA

---

Grc38\_ENST00000615433|processed\_transcript

---

Grc38\_ENST00000615499|miRNA

---

Grc38\_ENST00000615649|miRNA

---

Grc38\_ENST00000615913|miRNA

---

Grc38\_ENST00000615943|snRNA

---

Grc38\_ENST00000615947|lincRNA

---

Grc38\_ENST00000616044|miRNA

---

Grc38\_ENST00000616087|miRNA

---

Grc38\_ENST00000616090|miRNA

---

Grc38\_ENST00000616116|lincRNA

---

Grc38\_ENST00000616213|lincRNA

---

Grc38\_ENST00000616338|lincRNA

---

Grc38\_ENST00000616574|miRNA

---

Grc38\_ENST00000616576|lincRNA

---

Grc38\_ENST00000616774|processed\_transcript

---

Grc38\_ENST00000616937|miRNA

---

Grc38\_ENST00000616994|lincRNA

---

Grc38\_ENST00000617013|lincRNA

---

Grc38\_ENST00000617048|miRNA

---

Grc38\_ENST00000617117|lincRNA

---

Grc38\_ENST00000617174|miRNA

---

Grc38\_ENST00000617236|miRNA

---

Grc38\_ENST00000617323|lincRNA

---

Grc38\_ENST00000617352|processed\_transcript

---

Grc38\_ENST00000617415|sense\_intronic

---

Grc38\_ENST00000617439|sense\_intronic

---

Grc38\_ENST00000617489|lincRNA

---

Grc38\_ENST00000617518|processed\_transcript

---

Grc38\_ENST00000617525|miRNA

---

Grc38\_ENST00000617568|lincRNA

---

Grc38\_ENST00000617611|miRNA

---

Grc38\_ENST00000617627|lincRNA

---

Grc38\_ENST00000617646|miRNA

---

Grc38\_ENST00000617901|lincRNA

---

Grc38\_ENST00000618199|lincRNA

---

Grc38\_ENST00000618203|miRNA

---

Grc38\_ENST00000618256|lincRNA

---

Grc38\_ENST00000618285|lincRNA

---

Grc38\_ENST00000618303|lincRNA

---

Grc38\_ENST00000618311|processed\_transcript

---

Grc38\_ENST00000618408|miRNA

---

Grc38\_ENST00000618494|lincRNA

---

Grc38\_ENST00000618580|miRNA

---

Grc38\_ENST00000618647|miRNA

---

Grc38\_ENST00000618703|miRNA

---

Grc38\_ENST00000618745|miRNA

---

Grc38\_ENST00000618799|sense\_intronic

---

Grc38\_ENST00000618809|lincRNA

---

Grc38\_ENST00000618823|miRNA

---

Grc38\_ENST00000618835|sense\_intronic

---

Grc38\_ENST00000618966|lincRNA

---

Grc38\_ENST00000619029|miRNA

---

Grc38\_ENST00000619044|lincRNA

---

Grc38\_ENST00000619092|sense\_intronic

---

Grc38\_ENST00000619197|snRNA

---

Grc38\_ENST00000619226|sense\_intronic

---

Grc38\_ENST00000619442|lincRNA

---

Grc38\_ENST00000619486|sense\_intronic

---

Grc38\_ENST00000619545|miRNA

---

Grc38\_ENST00000619551|miRNA

---

Grc38\_ENST00000619634|miRNA

---

Grc38\_ENST00000619652|processed\_transcript

---

Grc38\_ENST00000619862|processed\_transcript

---

Grc38\_ENST00000619960|lincRNA

---

Grc38\_ENST00000620163|lincRNA

---

Grc38\_ENST00000620171|lincRNA

---

Grc38\_ENST00000620206|miRNA

---

Grc38\_ENST00000620246|lincRNA

---

Grc38\_ENST00000620266|lincRNA

---

Grc38\_ENST00000620272|lincRNA

---

Grc38\_ENST00000620274|sense\_intronic

---

Grc38\_ENST00000620377|lincRNA

---

Grc38\_ENST00000620472|sense\_intronic

---

Grc38\_ENST00000620495|lincRNA

---

Grc38\_ENST00000620503|sense\_intronic

---

Grc38\_ENST00000620519|lincRNA

---

Grc38\_ENST00000620527|miRNA

---

Grc38\_ENST00000620626|snRNA

---

Grc38\_ENST00000620677|miRNA

---

Grc38\_ENST00000620769|lincRNA

---

Grc38\_ENST00000620823|miRNA

---

Grc38\_ENST00000620883|miRNA

---

Grc38\_ENST00000620902|lincRNA

---

Grc38\_ENST00000621050|lincRNA

---

Grc38\_ENST00000621052|lincRNA

---

Grc38\_ENST00000621224|miRNA

---

Grc38\_ENST00000621373|miRNA

---

Grc38\_ENST00000621409|snRNA

---

Grc38\_ENST00000621423|sense\_intronic

---

Grc38\_ENST00000621451|miRNA

---

Grc38\_ENST00000621498|miRNA

---

Grc38\_ENST00000621541|miRNA

---

Grc38\_ENST00000621548|processed\_transcript

---

Grc38\_ENST00000621575|sense\_intronic

---

Grc38\_ENST00000621656|miRNA

---

Grc38\_ENST00000621919|lincRNA

---

Grc38\_ENST00000621929|lincRNA

---

Grc38\_ENST00000621942|lincRNA

---

Grc38\_ENST00000622091|sense\_intronic

---

Grc38\_ENST00000622355|processed\_transcript

---

Grc38\_ENST00000622359|lincRNA

---

Grc38\_ENST00000622426|miRNA

---

Grc38\_ENST00000622466|lincRNA

---

Grc38\_ENST00000622482|miRNA

---

Grc38\_ENST00000622485|miRNA

---

Grc38\_ENST00000622631|snoRNA

---

Grc38\_ENST00000622687|miRNA

---

Grc38\_ENST00000622725|miRNA

---

Grc38\_ENST00000622750|lincRNA

---

Grc38\_ENST00000622793|miRNA

---

Grc38\_ENST00000622794|lincRNA

---

Grc38\_ENST00000622846|miRNA

---

Grc38\_ENST00000622888|lincRNA

---

Grc38\_ENST00000622893|miRNA

---

Grc38\_ENST00000622966|sense\_intronic

---

Grc38\_ENST00000623040|processed\_transcript

---

Grc38\_ENST00000623047|lincRNA

---

Grc38\_ENST00000623075|sense\_overlapping

---

Grc38\_ENST00000623163|processed\_transcript

---

Grc38\_ENST00000623196|processed\_transcript

---

Grc38\_ENST00000623199|lincRNA

---

Grc38\_ENST00000623374|lincRNA

---

Grc38\_ENST00000623379|lincRNA

---

Grc38\_ENST00000623420|lincRNA

---

Grc38\_ENST00000623422|lincRNA

---

Grc38\_ENST00000623538|lincRNA

---

Grc38\_ENST00000623598|processed\_transcript

---

Grc38\_ENST00000623616|processed\_transcript

---

Grc38\_ENST00000623638|processed\_transcript

---

Grc38\_ENST00000623646|lincRNA

---

Grc38\_ENST00000623664|lincRNA

---

Grc38\_ENST00000623686|lincRNA

---

Grc38\_ENST00000623726|sense\_overlapping

---

Grc38\_ENST00000623740|sense\_overlapping

---

Grc38\_ENST00000623749|lincRNA

---

Grc38\_ENST00000623760|lincRNA

---

Grc38\_ENST00000623776|sense\_intronic

---

Grc38\_ENST00000623789|sense\_overlapping

---

Grc38\_ENST00000623860|lincRNA

---

Grc38\_ENST00000623892|lincRNA

---

Grc38\_ENST00000623901|processed\_transcript

---

Grc38\_ENST00000623947|lincRNA

---

Grc38\_ENST00000623956|lincRNA

---

Grc38\_ENST00000624060|processed\_transcript

---

Grc38\_ENST00000624080|processed\_transcript

---

Grc38\_ENST00000624219|lincRNA

---

Grc38\_ENST00000624224|lincRNA

---

Grc38\_ENST00000624264|lincRNA

---

Grc38\_ENST00000624295|processed\_transcript

---

Grc38\_ENST00000624442|lincRNA

---

Grc38\_ENST00000624444|lincRNA

---

Grc38\_ENST00000624617|lincRNA

---

Grc38\_ENST00000624632|lincRNA

---

Grc38\_ENST00000624818|sense\_intronic

---

Grc38\_ENST00000624835|lincRNA

---

Grc38\_ENST00000624867|sense\_intronic

---

Grc38\_ENST00000624938|miRNA

---

Grc38\_ENST00000624980|sense\_intronic

---

Grc38\_ENST00000625038|lincRNA

---

Grc38\_ENST00000625129|lincRNA

---

Grc38\_ENST00000625269|snoRNA

---

Grc38\_ENST00000625287|processed\_transcript

---

Grc38\_ENST00000625310|lincRNA

---

Grc38\_ENST00000625445|lincRNA

---

Grc38\_ENST00000625466|miRNA

---

Grc38\_ENST00000625542|miRNA

---

Grc38\_ENST00000625572|miRNA

---

Grc38\_ENST00000625598|lincRNA

---

Grc38\_ENST00000625652|miRNA

---

Grc38\_ENST00000625713|lincRNA

---

Grc38\_ENST00000625805|miRNA

---

Grc38\_ENST00000625861|miRNA

---

Grc38\_ENST00000625900|miRNA

---

Grc38\_ENST00000625967|miRNA

---

Grc38\_ENST00000625982|lincRNA

---

Grc38\_ENST00000626070|miRNA

---

Grc38\_ENST00000626086|miRNA

---

Grc38\_ENST00000626159|miRNA

---

Grc38\_ENST00000626187|miRNA

---

Grc38\_ENST00000626230|lincRNA

---

Grc38\_ENST00000626231|miRNA

---

Grc38\_ENST00000626286|lincRNA

---

Grc38\_ENST00000626324|miRNA

---

Grc38\_ENST00000626470|miRNA

---

Grc38\_ENST00000626616|miRNA

---

Grc38\_ENST00000626667|lincRNA

---

Grc38\_ENST00000626694|miRNA

---

Grc38\_ENST00000626708|miRNA

---

Grc38\_ENST00000626729|processed\_transcript

---

Grc38\_ENST00000626817|lincRNA

---

Grc38\_ENST00000626835|miRNA

---

Grc38\_ENST00000626913|miRNA

---

Grc38\_ENST00000626927|miRNA

---

Grc38\_ENST00000626977|sense\_intronic

---

Grc38\_ENST00000626980|miRNA

---

Grc38\_ENST00000627064|miRNA

---

Grc38\_ENST00000627119|miRNA

---

Grc38\_ENST00000627127|lincRNA

---

Grc38\_ENST00000627175|miRNA

---

Grc38\_ENST00000627253|snoRNA

---

Grc38\_ENST00000627369|miRNA

---

Grc38\_ENST00000627518|miRNA

---

Grc38\_ENST00000627566|lincRNA

---

Grc38\_ENST00000627629|miRNA

---

Grc38\_ENST00000627649|miRNA

---

Grc38\_ENST00000627702|miRNA

---

Grc38\_ENST00000627793|snRNA

---

Grc38\_ENST00000627823|processed\_transcript

---

Grc38\_ENST00000627879|miRNA

---

Grc38\_ENST00000627888|miRNA

---

Grc38\_ENST00000627933|snoRNA

---

Grc38\_ENST00000627943|miRNA

---

Grc38\_ENST00000627978|miRNA

---

Grc38\_ENST00000628013|processed\_transcript

---

Grc38\_ENST00000628105|miRNA

---

Grc38\_ENST00000628373|miRNA

---

Grc38\_ENST00000628381|miRNA

---

Grc38\_ENST00000628428|lincRNA

---

Grc38\_ENST00000628469|miRNA

---

Grc38\_ENST00000628570|miRNA

---

Grc38\_ENST00000628622|lincRNA

---

Grc38\_ENST00000628647|miRNA

---

Grc38\_ENST00000628653|miRNA

---

Grc38\_ENST00000628757|processed\_transcript

---

Grc38\_ENST00000628777|miRNA

---

Grc38\_ENST00000628893|miRNA

---

Grc38\_ENST00000629015|miRNA

---

Grc38\_ENST00000629061|miRNA

---

Grc38\_ENST00000629145|lincRNA

---

Grc38\_ENST00000629190|miRNA

---

Grc38\_ENST00000629248|miRNA

---

Grc38\_ENST00000629293|lincRNA

---

Grc38\_ENST00000629348|miRNA

---

Grc38\_ENST00000629393|sense\_intronic

---

Grc38\_ENST00000629398|miRNA

---

Grc38\_ENST00000629406|lincRNA

---

Grc38\_ENST00000629414|miRNA

---

Grc38\_ENST00000629461|sense\_intronic

---

Grc38\_ENST00000629564|sense\_overlapping

---

Grc38\_ENST00000629595|lincRNA

---

Grc38\_ENST00000629636|processed\_transcript

---

Grc38\_ENST00000629637|miRNA

---

Grc38\_ENST00000629651|lincRNA

---

Grc38\_ENST00000629732|lincRNA

---

Grc38\_ENST00000629772|miRNA

---

Grc38\_ENST00000629851|miRNA

---

Grc38\_ENST00000629887|miRNA

---

Grc38\_ENST00000629899|lincRNA

---

Grc38\_ENST00000630005|miRNA

---

Grc38\_ENST00000630034|sense\_intronic

---

Grc38\_ENST00000630054|sense\_intronic

---

Grc38\_ENST00000630075|miRNA

---

Grc38\_ENST00000630148|miRNA

---

Grc38\_ENST00000630162|lincRNA

---

Grc38\_ENST00000630193|miRNA

---

Grc38\_ENST00000630242|lincRNA

---

Grc38\_ENST00000630333|lincRNA

---

Grc38\_ENST00000630354|lincRNA

---

Grc38\_ENST00000630379|miRNA

---

Grc38\_ENST00000630402|miRNA

---

Grc38\_ENST00000630434|miRNA

---

Grc38\_ENST00000630509|miRNA

---

Grc38\_ENST00000630554|miRNA

---

Grc38\_ENST00000630558|lincRNA

---

Grc38\_ENST00000630737|snoRNA

---

Grc38\_ENST00000630765|miRNA

---

Grc38\_ENST00000630877|snoRNA

---

Grc38\_ENST00000630918|lincRNA

---

Grc38\_ENST00000630934|miRNA

---

Grc38\_ENST00000630946|lincRNA

---

Grc38\_ENST00000630990|miRNA

---

Grc38\_ENST00000631128|sense\_intronic

---

Grc38\_ENST00000631155|miRNA

---

Grc38\_ENST00000631169|lincRNA

---

Grc38\_ENST00000631175|processed\_transcript

---

Grc38\_ENST00000631190|lincRNA

---

Grc38\_ENST00000631211|lincRNA

---

Grc38\_ENST00000631235|lincRNA

---

Grc38\_ENST00000631261|miRNA

---

Grc38\_ENST00000631276|processed\_transcript

---

Grc38\_ENST00000631287|miRNA

---

Grc38\_ENST00000631329|miRNA

---

Grc38\_ENST00000631369|miRNA

---

lncrnadb\_cdr1-as\_homosapiens\_1|Homo

---

lncrnadb\_disc2\_homosapiens\_1|Homo

---

lncrnadb\_emx2os\_homosapiens\_1|Homo

---

lncrnadb\_lust\_homosapiens\_1|Homo

---

lncrnadb\_msur1\_musmusculus\_1|Mus

---

lncrnadb\_ncran\_hg\_1|Homo

---

lncrnadb\_pgsf1\_hg\_1|Homo

---

lncrnadb\_THRIL\_hg\_1|Homo

---

lncrnadb\_UM9-5\_hg\_1|Homo

---

mirBASE\_oha-miR-100-5p

---

piRNA|DQ570011

---

piRNA|DQ570485

---

piRNA|DQ570720

---

piRNA|DQ570812

---

piRNA|DQ570815

---

piRNA|DQ570989

---

piRNA|DQ570994

---

piRNA|DQ570996

---

piRNA|DQ571107

---

piRNA|DQ571526

---

piRNA|DQ571699

---

piRNA|DQ571873

---

piRNA|DQ572382

---

piRNA|DQ572385

---

piRNA|DQ572465

---

piRNA|DQ572467

---

piRNA|DQ572561

---

piRNA|DQ572566

---

piRNA|DQ573304

---

piRNA|DQ573352

---

piRNA|DQ574090

---

piRNA|DQ574254

---

piRNA|DQ574264

---

piRNA|DQ574391

---

piRNA|DQ574448

---

piRNA|DQ574511

---

piRNA|DQ574619

---

piRNA|DQ575036

---

piRNA|DQ575380

---

piRNA|DQ575721

---

piRNA|DQ576200

---

piRNA|DQ576377

---

piRNA|DQ576917

---

piRNA|DQ576952

---

piRNA|DQ577008

---

piRNA|DQ577216

---

piRNA|DQ577346

---

piRNA|DQ577620

---

piRNA|DQ578126

---

piRNA|DQ578516

---

piRNA|DQ578534

---

piRNA|DQ578787

---

piRNA|DQ578846

---

piRNA|DQ578918

---

piRNA|DQ579049

---

piRNA|DQ579110

---

piRNA|DQ579298

---

piRNA|DQ579415

---

piRNA|DQ579430

---

piRNA|DQ579855

---

piRNA|DQ580006

---

piRNA|DQ580140

---

piRNA|DQ580256

---

piRNA|DQ580576

---

piRNA|DQ580927

---

piRNA|DQ581351

---

piRNA|DQ581881

---

piRNA|DQ581894

---

piRNA|DQ582451

---

piRNA|DQ582657

---

piRNA|DQ582815

---

piRNA|DQ582827

---

piRNA|DQ582923

---

piRNA|DQ582940

---

piRNA|DQ583032

---

piRNA|DQ583045

---

piRNA|DQ583080

---

piRNA|DQ583324

---

piRNA|DQ583699

---

piRNA|DQ583836

---

piRNA|DQ584132

---

piRNA|DQ584144

---

piRNA|DQ584569

---

piRNA|DQ584635

---

piRNA|DQ584770

---

piRNA|DQ585261

---

piRNA|DQ585292

---

piRNA|DQ585299

---

piRNA|DQ585304

---

piRNA|DQ585628

---

piRNA|DQ585854

---

piRNA|DQ586098

---

piRNA|DQ586108

---

piRNA|DQ586183

---

piRNA|DQ586566

---

piRNA|DQ586628

---

piRNA|DQ586741

---

piRNA|DQ586935

---

piRNA|DQ587229

---

piRNA|DQ587402

---

piRNA|DQ587425

---

piRNA|DQ588525

---

piRNA|DQ589377

---

piRNA|DQ589692

---

piRNA|DQ589802

---

piRNA|DQ590011

---

piRNA|DQ590387

---

piRNA|DQ590404

---

piRNA|DQ590492

---

piRNA|DQ590642

---

piRNA|DQ591115

---

piRNA|DQ591773

---

piRNA|DQ591959

---

piRNA|DQ592016

---

piRNA|DQ592048

---

piRNA|DQ592146

---

piRNA|DQ592148

---

piRNA|DQ592293

---

piRNA|DQ592393

---

piRNA|DQ592852

---

piRNA|DQ592859

---

piRNA|DQ593039

---

piRNA|DQ593158

---

piRNA|DQ593431

---

piRNA|DQ593888

---

piRNA|DQ594117

---

piRNA|DQ594176

---

piRNA|DQ594273

---

piRNA|DQ594522

---

piRNA|DQ594655

---

piRNA|DQ594983

---

piRNA|DQ594989

---

piRNA|DQ595111

---

piRNA|DQ595197

---

piRNA|DQ595229

---

piRNA|DQ595297

---

piRNA|DQ595353

---

piRNA|DQ595425

---

piRNA|DQ595432

---

piRNA|DQ595445

---

piRNA|DQ595788

---

piRNA|DQ595848

---

piRNA|DQ596044

---

piRNA|DQ596183

---

piRNA|DQ596408

---

piRNA|DQ596988

---

piRNA|DQ596992

---

piRNA|DQ597086

---

piRNA|DQ597228

---

piRNA|DQ597484

---

piRNA|DQ597945

---

piRNA|DQ597960

---

piRNA|DQ597970

---

piRNA|DQ597997

---

piRNA|DQ598016

---

piRNA|DQ598041

---

piRNA|DQ598072

---

piRNA|DQ598263

---

piRNA|DQ598300

---

piRNA|DQ598409

---

piRNA|DQ598410

---

piRNA|DQ598951

---

piRNA|DQ599034

---

piRNA|DQ599058

---

piRNA|DQ599147

---

piRNA|DQ599501

---

piRNA|DQ599791

---

piRNA|DQ599818

---

piRNA|DQ599865

---

piRNA|DQ600373

---

piRNA|DQ600380

---

piRNA|DQ600689

---

piRNA|DQ600708

---

piRNA|DQ600754

---

piRNA|DQ601078

---

piRNA|DQ601103

---

piRNA|DQ601350

---

piRNA|DQ601906

---

siRNA\_0\_4501882

---

siRNA\_1045\_39812410

---

siRNA\_177\_4885510

---

siRNA\_277\_7669491

---

siRNA\_308\_10834981

---

siRNA\_496\_14277699

---

siRNA\_499\_14591908

---

siRNA\_515\_14591914

---

siRNA\_516\_14591915

---

siRNA\_520\_14591916

---

siRNA\_521\_14670385

---

siRNA\_531\_14790142

---

siRNA\_535\_14916502

---

siRNA\_537\_15011935

---

siRNA\_539\_15011937

---

siRNA\_563\_15718688

---

siRNA\_575\_16306563

---

siRNA\_58\_4506760

---

siRNA\_580\_16905512

---

siRNA\_599\_17158043

---

siRNA\_637\_19743822

---

siRNA\_802\_24475893

---

siRNA\_820\_27436945

---

siRNA\_965\_34335149

**Elements in Diff Represented in CAF-CELL vs CAF-EXO, Not Significant in NF-CELL vs NF-EXO and Not Significant in CAF-CELL vs CAF-EXO**

**Elements in Diff Represented in CAF-CELL vs CAF-EXO, Not Significant in NF-CELL vs NF-EXO, Not Significant in CAF-CELL vs CAF-EXO and Diff Represented in NF-CELL vs NF-EXO**

**Elements in Diff Represented in CAF-CELL vs CAF-EXO and Diff Represented in NF-CELL vs NF-EXO**
  


---

Grc38\_ENST00000229465|lincRNA

---

Grc38\_ENST00000313495|lincRNA

---

Grc38\_ENST00000349529|miRNA

---

Grc38\_ENST00000356047|lincRNA

---

Grc38\_ENST00000362102|miRNA

---

Grc38\_ENST00000362111|miRNA

---

Grc38\_ENST00000362114|miRNA

---

Grc38\_ENST00000362117|miRNA

---

Grc38\_ENST00000362135|miRNA

---

Grc38\_ENST00000362145|miRNA

---

Grc38\_ENST00000362150|miRNA

---

Grc38\_ENST00000362153|miRNA

---

Grc38\_ENST00000362155|miRNA

---

Grc38\_ENST00000362159|miRNA

---

Grc38\_ENST00000362165|miRNA

---

Grc38\_ENST00000362181|miRNA

---

Grc38\_ENST00000362183|miRNA

---

Grc38\_ENST00000362202|miRNA

---

Grc38\_ENST00000362205|miRNA

---

Grc38\_ENST00000362222|miRNA

---

Grc38\_ENST00000362224|miRNA

---

Grc38\_ENST00000362239|miRNA

---

Grc38\_ENST00000362251|miRNA

---

Grc38\_ENST00000362252|miRNA

---

Grc38\_ENST00000362260|miRNA

---

Grc38\_ENST00000362262|miRNA

---

Grc38\_ENST00000362279|miRNA

---

Grc38\_ENST00000362280|miRNA

---

Grc38\_ENST00000362281|miRNA

---

Grc38\_ENST00000362302|miRNA

---

Grc38\_ENST00000362307|miRNA

---

Grc38\_ENST00000362309|miRNA

---

Grc38\_ENST00000362310|miRNA

---

Grc38\_ENST00000362412|snoRNA

---

Grc38\_ENST00000362443|snRNA

---

Grc38\_ENST00000362477|snRNA

---

Grc38\_ENST00000362507|snRNA

---

Grc38\_ENST00000362607|snoRNA

---

Grc38\_ENST00000362698|snRNA

---

Grc38\_ENST00000362704|snoRNA

---

Grc38\_ENST00000362705|snoRNA

---

Grc38\_ENST00000362723|snoRNA

---

Grc38\_ENST00000362761|snoRNA

---

Grc38\_ENST00000362803|snoRNA

---

Grc38\_ENST00000362805|snoRNA

---

Grc38\_ENST00000362883|snoRNA

---

Grc38\_ENST00000363064|snoRNA

---

Grc38\_ENST00000363091|snoRNA

---

Grc38\_ENST00000363202|snoRNA

---

Grc38\_ENST00000363214|snoRNA

---

Grc38\_ENST00000363217|snoRNA

---

Grc38\_ENST00000363286|snRNA

---

Grc38\_ENST00000363299|snRNA

---

Grc38\_ENST00000363315|snoRNA

---

Grc38\_ENST00000363345|snoRNA

---

Grc38\_ENST00000363389|snoRNA

---

Grc38\_ENST00000363450|snoRNA

---

Grc38\_ENST00000363485|snoRNA

---

Grc38\_ENST00000363536|snoRNA

---

Grc38\_ENST00000363543|snoRNA

---

Grc38\_ENST00000363593|snoRNA

---

Grc38\_ENST00000363610|snoRNA

---

Grc38\_ENST00000363626|snoRNA

---

Grc38\_ENST00000363660|snoRNA

---

Grc38\_ENST00000363664|snoRNA

---

Grc38\_ENST00000363742|snoRNA

---

Grc38\_ENST00000363753|snoRNA

---

Grc38\_ENST00000363836|snoRNA

---

Grc38\_ENST00000363925|snRNA

---

Grc38\_ENST00000363981|snoRNA

---

Grc38\_ENST00000364009|snoRNA

---

Grc38\_ENST00000364027|snoRNA

---

Grc38\_ENST00000364043|snoRNA

---

Grc38\_ENST00000364089|snoRNA

---

Grc38\_ENST00000364113|snoRNA

---

Grc38\_ENST00000364139|snoRNA

---

Grc38\_ENST00000364259|snoRNA

---

Grc38\_ENST00000364294|snRNA

---

Grc38\_ENST00000364310|snRNA

---

Grc38\_ENST00000364370|snoRNA

---

Grc38\_ENST00000364432|snoRNA

---

Grc38\_ENST00000364533|snoRNA

---

Grc38\_ENST00000364569|snRNA

---

Grc38\_ENST00000364578|snoRNA

---

Grc38\_ENST00000364617|snoRNA

---

Grc38\_ENST00000364773|snoRNA

---

Grc38\_ENST00000364802|snoRNA

---

Grc38\_ENST00000364805|snoRNA

---

Grc38\_ENST00000364849|snoRNA

---

Grc38\_ENST00000364931|snRNA

---

Grc38\_ENST00000364938|snoRNA

---

Grc38\_ENST00000364953|snoRNA

---

Grc38\_ENST00000364969|snoRNA

---

Grc38\_ENST00000364977|snoRNA

---

Grc38\_ENST00000365028|snoRNA

---

Grc38\_ENST00000365080|snoRNA

---

Grc38\_ENST00000365128|snoRNA

---

Grc38\_ENST00000365153|snoRNA

---

Grc38\_ENST00000365161|snoRNA

---

Grc38\_ENST00000365172|snoRNA

---

Grc38\_ENST00000365178|snoRNA

---

Grc38\_ENST00000365223|snoRNA

---

Grc38\_ENST00000365382|snoRNA

---

Grc38\_ENST00000365400|snoRNA

---

Grc38\_ENST00000365423|snoRNA

---

Grc38\_ENST00000365444|snoRNA

---

Grc38\_ENST00000365530|snoRNA

---

Grc38\_ENST00000365574|snRNA

---

Grc38\_ENST00000365607|snoRNA

---

Grc38\_ENST00000365633|snoRNA

---

Grc38\_ENST00000365659|snoRNA

---

Grc38\_ENST00000365668|snRNA

---

Grc38\_ENST00000365699|miRNA

---

Grc38\_ENST00000383860|snRNA

---

Grc38\_ENST00000383870|snoRNA

---

Grc38\_ENST00000383875|snoRNA

---

Grc38\_ENST00000383885|snoRNA

---

Grc38\_ENST00000383903|snoRNA

---

Grc38\_ENST00000383953|snoRNA

---

Grc38\_ENST00000384027|snoRNA

---

Grc38\_ENST00000384033|snoRNA

---

Grc38\_ENST00000384048|snoRNA

---

Grc38\_ENST00000384084|snoRNA

---

Grc38\_ENST00000384096|snoRNA

---

Grc38\_ENST00000384136|snRNA

---

Grc38\_ENST00000384147|snoRNA

---

Grc38\_ENST00000384158|snoRNA

---

Grc38\_ENST00000384176|snoRNA

---

Grc38\_ENST00000384214|snoRNA

---

Grc38\_ENST00000384215|snoRNA

---

Grc38\_ENST00000384220|snoRNA

---

Grc38\_ENST00000384229|snoRNA

---

Grc38\_ENST00000384252|snoRNA

---

Grc38\_ENST00000384262|snoRNA

---

Grc38\_ENST00000384287|snoRNA

---

Grc38\_ENST00000384304|snoRNA

---

Grc38\_ENST00000384320|snoRNA

---

Grc38\_ENST00000384334|snoRNA

---

Grc38\_ENST00000384339|snoRNA

---

Grc38\_ENST00000384342|snoRNA

---

Grc38\_ENST00000384356|snoRNA

---

Grc38\_ENST00000384360|snoRNA

---

Grc38\_ENST00000384365|snoRNA

---

Grc38\_ENST00000384388|snRNA

---

Grc38\_ENST00000384390|snoRNA

---

Grc38\_ENST00000384401|snoRNA

---

Grc38\_ENST00000384416|snoRNA

---

Grc38\_ENST00000384437|snoRNA

---

Grc38\_ENST00000384452|snoRNA

---

Grc38\_ENST00000384512|snoRNA

---

Grc38\_ENST00000384574|snoRNA

---

Grc38\_ENST00000384581|snoRNA

---

Grc38\_ENST00000384606|snRNA

---

Grc38\_ENST00000384662|snoRNA

---

Grc38\_ENST00000384693|snoRNA

---

Grc38\_ENST00000384706|snoRNA

---

Grc38\_ENST00000384714|snoRNA

---

Grc38\_ENST00000384723|snRNA

---

Grc38\_ENST00000384756|snoRNA

---

Grc38\_ENST00000384765|snoRNA

---

Grc38\_ENST00000384769|snoRNA

---

Grc38\_ENST00000384792|snoRNA

---

Grc38\_ENST00000384816|miRNA

---

Grc38\_ENST00000384831|miRNA

---

Grc38\_ENST00000384832|miRNA

---

Grc38\_ENST00000384849|miRNA

---

Grc38\_ENST00000384850|miRNA

---

Grc38\_ENST00000384852|miRNA

---

Grc38\_ENST00000384865|miRNA

---

Grc38\_ENST00000384876|miRNA

---

Grc38\_ENST00000384885|miRNA

---

Grc38\_ENST00000384892|miRNA

---

Grc38\_ENST00000384907|miRNA

---

Grc38\_ENST00000384967|miRNA

---

Grc38\_ENST00000384976|miRNA

---

Grc38\_ENST00000384988|miRNA

---

Grc38\_ENST00000384993|miRNA

---

Grc38\_ENST00000384999|miRNA

---

Grc38\_ENST00000385006|miRNA

---

Grc38\_ENST00000385010|miRNA

---

Grc38\_ENST00000385012|miRNA

---

Grc38\_ENST00000385016|miRNA

---

Grc38\_ENST00000385019|miRNA

---

Grc38\_ENST00000385020|miRNA

---

Grc38\_ENST00000385021|miRNA

---

Grc38\_ENST00000385022|miRNA

---

Grc38\_ENST00000385024|miRNA

---

Grc38\_ENST00000385028|miRNA

---

Grc38\_ENST00000385029|miRNA

---

Grc38\_ENST00000385045|miRNA

---

Grc38\_ENST00000385051|miRNA

---

Grc38\_ENST00000385054|miRNA

---

Grc38\_ENST00000385073|miRNA

---

Grc38\_ENST00000385092|miRNA

---

Grc38\_ENST00000385128|miRNA

---

Grc38\_ENST00000385129|miRNA

---

Grc38\_ENST00000385130|miRNA

---

Grc38\_ENST00000385135|miRNA

---

Grc38\_ENST00000385207|miRNA

---

Grc38\_ENST00000385212|miRNA

---

Grc38\_ENST00000385214|miRNA

---

Grc38\_ENST00000385227|miRNA

---

Grc38\_ENST00000385233|miRNA

---

Grc38\_ENST00000385235|miRNA

---

Grc38\_ENST00000385236|miRNA

---

Grc38\_ENST00000385243|miRNA

---

Grc38\_ENST00000385245|miRNA

---

Grc38\_ENST00000385254|miRNA

---

Grc38\_ENST00000385266|miRNA

---

Grc38\_ENST00000385270|miRNA

---

Grc38\_ENST00000385271|miRNA

---

Grc38\_ENST00000385273|miRNA

---

Grc38\_ENST00000385274|miRNA

---

Grc38\_ENST00000385277|miRNA

---

Grc38\_ENST00000385280|miRNA

---

Grc38\_ENST00000385282|miRNA

---

Grc38\_ENST00000385288|miRNA

---

Grc38\_ENST00000385289|miRNA

---

Grc38\_ENST00000385300|miRNA

---

Grc38\_ENST00000385301|miRNA

---

Grc38\_ENST00000386037|snoRNA

---

Grc38\_ENST00000386062|snoRNA

---

Grc38\_ENST00000386157|snoRNA

---

Grc38\_ENST00000386307|snoRNA

---

Grc38\_ENST00000386683|snoRNA

---

Grc38\_ENST00000386745|snoRNA

---

Grc38\_ENST00000386747|snoRNA

---

Grc38\_ENST00000386847|snoRNA

---

Grc38\_ENST00000386967|snoRNA

---

Grc38\_ENST00000386972|miRNA

---

Grc38\_ENST00000390183|miRNA

---

Grc38\_ENST00000390204|miRNA

---

Grc38\_ENST00000390225|miRNA

---

Grc38\_ENST00000390227|miRNA

---

Grc38\_ENST00000390708|miRNA

---

Grc38\_ENST00000390738|miRNA

---

Grc38\_ENST00000390842|snoRNA

---

Grc38\_ENST00000390856|snoRNA

---

Grc38\_ENST00000390861|snoRNA

---

Grc38\_ENST00000390930|snoRNA

---

Grc38\_ENST00000390981|snoRNA

---

Grc38\_ENST00000391002|snoRNA

---

Grc38\_ENST00000391007|snoRNA

---

Grc38\_ENST00000391076|snoRNA

---

Grc38\_ENST00000391079|snoRNA

---

Grc38\_ENST00000391100|snoRNA

---

Grc38\_ENST00000391141|snoRNA

---

Grc38\_ENST00000391145|snoRNA

---

Grc38\_ENST00000391150|snoRNA

---

Grc38\_ENST00000391162|snoRNA

---

Grc38\_ENST00000391208|snRNA

---

Grc38\_ENST00000391232|snoRNA

---

Grc38\_ENST00000391286|snoRNA

---

Grc38\_ENST00000400436|processed\_transcript

---

Grc38\_ENST00000401190|miRNA

---

Grc38\_ENST00000408061|snoRNA

---

Grc38\_ENST00000408136|miRNA

---

Grc38\_ENST00000408189|snoRNA

---

Grc38\_ENST00000408314|snoRNA

---

Grc38\_ENST00000408373|snoRNA

---

Grc38\_ENST00000408376|snoRNA

---

Grc38\_ENST00000408564|snoRNA

---

Grc38\_ENST00000408573|snoRNA

---

Grc38\_ENST00000408587|snoRNA

---

Grc38\_ENST00000408612|snoRNA

---

Grc38\_ENST00000408749|snRNA

---

Grc38\_ENST00000408813|snoRNA

---

Grc38\_ENST00000408827|miRNA

---

Grc38\_ENST00000410144|snRNA

---

Grc38\_ENST00000410361|snRNA

---

Grc38\_ENST00000410396|snRNA

---

Grc38\_ENST00000410413|snoRNA

---

Grc38\_ENST00000410433|snoRNA

---

Grc38\_ENST00000410457|snRNA

---

Grc38\_ENST00000410482|snRNA

---

Grc38\_ENST00000410545|snRNA

---

Grc38\_ENST00000410712|snRNA

---

Grc38\_ENST00000410818|snRNA

---

Grc38\_ENST00000410991|snRNA

---

Grc38\_ENST00000411053|snRNA

---

Grc38\_ENST00000411292|snoRNA

---

Grc38\_ENST00000411315|snRNA

---

Grc38\_ENST00000411404|snRNA

---

Grc38\_ENST00000412059|processed\_transcript

---

Grc38\_ENST00000413522|snoRNA

---

Grc38\_ENST00000416952|processed\_transcript

---

Grc38\_ENST00000421068|processed\_transcript

---

Grc38\_ENST00000421202|processed\_transcript

---

Grc38\_ENST00000422183|processed\_transcript

---

Grc38\_ENST00000422207|processed\_transcript

---

Grc38\_ENST00000427501|processed\_transcript

---

Grc38\_ENST00000428514|snoRNA

---

Grc38\_ENST00000430247|lincRNA

---

Grc38\_ENST00000430728|lincRNA

---

Grc38\_ENST00000431043|processed\_transcript

---

Grc38\_ENST00000431268|processed\_transcript

---

Grc38\_ENST00000433310|lincRNA

---

Grc38\_ENST00000434796|processed\_transcript

---

Grc38\_ENST00000436656|processed\_transcript

---

Grc38\_ENST00000437681|sense\_intronic

---

Grc38\_ENST00000439232|snoRNA

---

Grc38\_ENST00000442067|processed\_transcript

---

Grc38\_ENST00000443799|processed\_transcript

---

Grc38\_ENST00000444470|processed\_transcript

---

Grc38\_ENST00000445646|lincRNA

---

Grc38\_ENST00000448188|snoRNA

---

Grc38\_ENST00000449589|processed\_transcript

---

Grc38\_ENST00000451607|processed\_transcript

---

Grc38\_ENST00000453784|lincRNA

---

Grc38\_ENST00000454068|processed\_transcript

---

Grc38\_ENST00000454813|processed\_transcript

---

Grc38\_ENST00000456293|processed\_transcript

---

Grc38\_ENST00000456812|processed\_transcript

---

Grc38\_ENST00000458770|snoRNA

---

Grc38\_ENST00000458797|scaRNA

---

Grc38\_ENST00000458838|snoRNA

---

Grc38\_ENST00000458892|snoRNA

---

Grc38\_ENST00000458893|snoRNA

---

Grc38\_ENST00000458974|snoRNA

---

Grc38\_ENST00000459083|snoRNA

---

Grc38\_ENST00000459124|snoRNA

---

Grc38\_ENST00000459155|snoRNA

---

Grc38\_ENST00000459159|snoRNA

---

Grc38\_ENST00000459163|snoRNA

---

Grc38\_ENST00000459174|snoRNA

---

Grc38\_ENST00000459187|snoRNA

---

Grc38\_ENST00000459299|snoRNA

---

Grc38\_ENST00000459342|snoRNA

---

Grc38\_ENST00000459386|snoRNA

---

Grc38\_ENST00000459433|snoRNA

---

Grc38\_ENST00000459473|snoRNA

---

Grc38\_ENST00000459475|snoRNA

---

Grc38\_ENST00000459579|snoRNA

---

Grc38\_ENST00000459584|snoRNA

---

Grc38\_ENST00000459623|snoRNA

---

Grc38\_ENST00000460249|processed\_transcript

---

Grc38\_ENST00000480811|processed\_transcript

---

Grc38\_ENST00000491009|processed\_transcript

---

Grc38\_ENST00000492250|processed\_transcript

---

Grc38\_ENST00000497774|processed\_transcript

---

Grc38\_ENST00000501122|lincRNA

---

Grc38\_ENST00000503991|scaRNA

---

Grc38\_ENST00000505089|lincRNA

---

Grc38\_ENST00000505219|snoRNA

---

Grc38\_ENST00000508832|lincRNA

---

Grc38\_ENST00000510505|lincRNA

---

Grc38\_ENST00000515909|snRNA

---

Grc38\_ENST00000516060|scaRNA

---

Grc38\_ENST00000516089|scaRNA

---

Grc38\_ENST00000516146|snRNA

---

Grc38\_ENST00000516209|snRNA

---

Grc38\_ENST00000516327|snoRNA

---

Grc38\_ENST00000516336|snRNA

---

Grc38\_ENST00000516438|snRNA

---

Grc38\_ENST00000516528|snoRNA

---

Grc38\_ENST00000516564|snRNA

---

Grc38\_ENST00000516672|scaRNA

---

Grc38\_ENST00000516768|scaRNA

---

Grc38\_ENST00000516881|scaRNA

---

Grc38\_ENST00000517038|snRNA

---

Grc38\_ENST00000517041|snRNA

---

Grc38\_ENST00000517097|scaRNA

---

Grc38\_ENST00000517138|scaRNA

---

Grc38\_ENST00000517242|snoRNA

---

Grc38\_ENST00000517277|snRNA

---

Grc38\_ENST00000517961|lincRNA

---

Grc38\_ENST00000521127|processed\_transcript

---

Grc38\_ENST00000534336|lincRNA

---

Grc38\_ENST00000535076|processed\_transcript

---

Grc38\_ENST00000537024|processed\_transcript

---

Grc38\_ENST00000537925|processed\_transcript

---

Grc38\_ENST00000537965|processed\_transcript

---

Grc38\_ENST00000538654|processed\_transcript

---

Grc38\_ENST00000540725|processed\_transcript

---

Grc38\_ENST00000540865|processed\_transcript

---

Grc38\_ENST00000540904|processed\_transcript

---

Grc38\_ENST00000541416|processed\_transcript

---

Grc38\_ENST00000541782|sense\_intronic

---

Grc38\_ENST00000544550|processed\_transcript

---

Grc38\_ENST00000544868|lincRNA

---

Grc38\_ENST00000544983|processed\_transcript

---

Grc38\_ENST00000545308|processed\_transcript

---

Grc38\_ENST00000545688|processed\_transcript

---

Grc38\_ENST00000545920|processed\_transcript

---

Grc38\_ENST00000549804|processed\_transcript

---

Grc38\_ENST00000551361|processed\_transcript

---

Grc38\_ENST00000553465|lincRNA

---

Grc38\_ENST00000554693|lincRNA

---

Grc38\_ENST00000555004|lincRNA

---

Grc38\_ENST00000561320|lincRNA

---

Grc38\_ENST00000561622|3prime\_overlapping\_ncrna

---

Grc38\_ENST00000562952|sense\_overlapping

---

Grc38\_ENST00000567488|lincRNA

---

Grc38\_ENST00000567527|lincRNA

---

Grc38\_ENST00000571722|snoRNA

---

Grc38\_ENST00000573259|processed\_transcript

---

Grc38\_ENST00000573457|processed\_transcript

---

Grc38\_ENST00000574616|3prime\_overlapping\_ncrna

---

Grc38\_ENST00000574846|processed\_transcript

---

Grc38\_ENST00000575226|processed\_transcript

---

Grc38\_ENST00000575766|processed\_transcript

---

Grc38\_ENST00000576096|processed\_transcript

---

Grc38\_ENST00000576410|processed\_transcript

---

Grc38\_ENST00000576702|processed\_transcript

---

Grc38\_ENST00000577122|processed\_transcript

---

Grc38\_ENST00000577700|lincRNA

---

Grc38\_ENST00000577887|snoRNA

---

Grc38\_ENST00000577988|snoRNA

---

Grc38\_ENST00000578757|processed\_transcript

---

Grc38\_ENST00000579017|miRNA

---

Grc38\_ENST00000579879|snoRNA

---

Grc38\_ENST00000580533|snoRNA

---

Grc38\_ENST00000580972|snRNA

---

Grc38\_ENST00000581525|snoRNA

---

Grc38\_ENST00000582661|miRNA

---

Grc38\_ENST00000582890|miRNA

---

Grc38\_ENST00000582965|lincRNA

---

Grc38\_ENST00000583032|snoRNA

---

Grc38\_ENST00000583619|snoRNA

---

Grc38\_ENST00000583861|snoRNA

---

Grc38\_ENST00000584275|snoRNA

---

Grc38\_ENST00000584923|snoRNA

---

Grc38\_ENST00000584949|snoRNA

---

Grc38\_ENST00000586185|lincRNA

---

Grc38\_ENST00000586231|lincRNA

---

Grc38\_ENST00000589496|3prime\_overlapping\_ncrna

---

Grc38\_ENST00000591384|lincRNA

---

Grc38\_ENST00000591554|miRNA

---

Grc38\_ENST00000602361|lincRNA

---

Grc38\_ENST00000602478|lincRNA

---

Grc38\_ENST00000602573|lincRNA

---

Grc38\_ENST00000602755|lincRNA

---

Grc38\_ENST00000604135|sense\_intronic

---

Grc38\_ENST00000605502|lincRNA

---

Grc38\_ENST00000605533|sense\_intronic

---

Grc38\_ENST00000606190|snRNA

---

Grc38\_ENST00000606349|miRNA

---

Grc38\_ENST00000606412|snoRNA

---

Grc38\_ENST00000606577|snoRNA

---

Grc38\_ENST00000606623|snRNA

---

Grc38\_ENST00000606769|snoRNA

---

Grc38\_ENST00000607313|snoRNA

---

Grc38\_ENST00000607520|processed\_transcript

---

Grc38\_ENST00000607707|snoRNA

---

Grc38\_ENST00000608412|lincRNA

---

Grc38\_ENST00000609276|lincRNA

---

Grc38\_ENST00000610851|lincRNA

---

Grc38\_ENST00000611296|lincRNA

---

Grc38\_ENST00000612496|lincRNA

---

Grc38\_ENST00000613527|miRNA

---

Grc38\_ENST00000613917|lincRNA

---

Grc38\_ENST00000613956|snRNA

---

Grc38\_ENST00000614083|snRNA

---

Grc38\_ENST00000615356|snRNA

---

Grc38\_ENST00000616315|lincRNA

---

Grc38\_ENST00000616527|lincRNA

---

Grc38\_ENST00000616691|lincRNA

---

Grc38\_ENST00000617320|miRNA

---

Grc38\_ENST00000618132|lincRNA

---

Grc38\_ENST00000618227|lincRNA

---

Grc38\_ENST00000618589|lincRNA

---

Grc38\_ENST00000618925|lincRNA

---

Grc38\_ENST00000618978|snRNA

---

Grc38\_ENST00000619178|snoRNA

---

Grc38\_ENST00000619225|snRNA

---

Grc38\_ENST00000619449|lincRNA

---

Grc38\_ENST00000620232|snoRNA

---

Grc38\_ENST00000620268|snRNA

---

Grc38\_ENST00000620446|snoRNA

---

Grc38\_ENST00000620465|lincRNA

---

Grc38\_ENST00000621466|miRNA

---

Grc38\_ENST00000621667|miRNA

---

Grc38\_ENST00000621753|snRNA

---

Grc38\_ENST00000622286|snRNA

---

Grc38\_ENST00000622328|lincRNA

---

Grc38\_ENST00000623391|lincRNA

---

Grc38\_ENST00000625314|snoRNA

---

Grc38\_ENST00000625845|snoRNA

---

Grc38\_ENST00000625876|snoRNA

---

Grc38\_ENST00000625943|snoRNA

---

Grc38\_ENST00000626826|macro\_lncRNA

---

Grc38\_ENST00000626830|miRNA

---

Grc38\_ENST00000626886|snoRNA

---

Grc38\_ENST00000626963|snoRNA

---

Grc38\_ENST00000627324|scaRNA

---

Grc38\_ENST00000627983|snoRNA

---

Grc38\_ENST00000628177|snoRNA

---

Grc38\_ENST00000628458|snoRNA

---

Grc38\_ENST00000628672|miRNA

---

Grc38\_ENST00000628908|miRNA

---

Grc38\_ENST00000629038|snoRNA

---

Grc38\_ENST00000629045|scaRNA

---

Grc38\_ENST00000629167|miRNA

---

Grc38\_ENST00000629259|miRNA

---

Grc38\_ENST00000629295|lincRNA

---

Grc38\_ENST00000629478|snRNA

---

Grc38\_ENST00000629536|snoRNA

---

Grc38\_ENST00000629629|snoRNA

---

Grc38\_ENST00000629784|miRNA

---

Grc38\_ENST00000630092|snoRNA

---

Grc38\_ENST00000630110|miRNA

---

Grc38\_ENST00000630429|snoRNA

---

Grc38\_ENST00000630949|snoRNA

---

Grc38\_ENST00000631292|snoRNA

---

lncrnadb\_7sk\_homosapiens\_1|Homo

---

lncrnadb\_kcnq1ot1\_homosapiens\_1|Homo

---

lncrnadb\_neat1\_homosapiens\_2|Homo

---

mirBASE\_efT-miR-26c

---

mirBASE\_tch-miR-27a-3p

---

piRNA|DQ569993

---

piRNA|DQ571511

---

piRNA|DQ582536

---

piRNA|DQ587514

---

piRNA|DQ598646

---

snoRNADB\_E3

---

snoRNADB\_HBII-135

---

snoRNADB\_HBII-202

---

snoRNADB\_U104

---

snoRNADB\_U17b

---

Grc38\_ENST00000360737|lincRNA

---

Grc38\_ENST00000362291|miRNA

---

Grc38\_ENST00000363306|snRNA

---

Grc38\_ENST00000363334|snRNA

---

Grc38\_ENST00000363426|snRNA

---

Grc38\_ENST00000364300|snRNA

---

Grc38\_ENST00000364421|snRNA

---

Grc38\_ENST00000365477|snRNA

---

Grc38\_ENST00000383858|snRNA

---

Grc38\_ENST00000383975|snRNA

---

Grc38\_ENST00000384093|snRNA

---

Grc38\_ENST00000384425|snRNA

---

Grc38\_ENST00000384446|snRNA

---

Grc38\_ENST00000384499|snRNA

---

Grc38\_ENST00000384550|snoRNA

---

Grc38\_ENST00000384592|snRNA

---

Grc38\_ENST00000384619|snRNA

---

Grc38\_ENST00000385044|miRNA

---

Grc38\_ENST00000385302|miRNA

---

Grc38\_ENST00000387069|snRNA

---

Grc38\_ENST00000408240|miRNA

---

Grc38\_ENST00000410794|snRNA

---

Grc38\_ENST00000415386|lincRNA

---

Grc38\_ENST00000423943|lincRNA

---

Grc38\_ENST00000427111|lincRNA

---

Grc38\_ENST00000443364|lincRNA

---

Grc38\_ENST00000445817|lincRNA

---

Grc38\_ENST00000456105|lincRNA

---

Grc38\_ENST00000459170|miRNA

---

Grc38\_ENST00000459274|snRNA

---

Grc38\_ENST00000501143|lincRNA

---

Grc38\_ENST00000511821|lincRNA

---

Grc38\_ENST00000527474|lincRNA

---

Grc38\_ENST00000559030|lincRNA

---

Grc38\_ENST00000563151|lincRNA

---

Grc38\_ENST00000565467|lincRNA

---

Grc38\_ENST00000568394|lincRNA

---

Grc38\_ENST00000569832|processed\_transcript

---

Grc38\_ENST00000573479|lincRNA

---

Grc38\_ENST00000577388|miRNA

---

Grc38\_ENST00000578924|miRNA

---

Grc38\_ENST00000579933|lincRNA

---

Grc38\_ENST00000580233|miRNA

---

Grc38\_ENST00000580344|miRNA

---

Grc38\_ENST00000581316|miRNA

---

Grc38\_ENST00000581364|miRNA

---

Grc38\_ENST00000582082|miRNA

---

Grc38\_ENST00000582090|miRNA

---

Grc38\_ENST00000582216|miRNA

---

Grc38\_ENST00000583357|miRNA

---

Grc38\_ENST00000584072|miRNA

---

Grc38\_ENST00000584178|miRNA

---

Grc38\_ENST00000584443|miRNA

---

Grc38\_ENST00000593917|processed\_transcript

---

Grc38\_ENST00000602301|vaultRNA

---

Grc38\_ENST00000604849|lincRNA

---

Grc38\_ENST00000605806|snRNA

---

Grc38\_ENST00000609880|lincRNA

---

Grc38\_ENST00000610122|lincRNA

---

Grc38\_ENST00000610524|miRNA

---

Grc38\_ENST00000611066|miRNA

---

Grc38\_ENST00000611300|sRNA

---

Grc38\_ENST00000612171|miRNA

---

Grc38\_ENST00000612766|miRNA

---

Grc38\_ENST00000613558|miRNA

---

Grc38\_ENST00000617238|miRNA

---

Grc38\_ENST00000617702|lincRNA

---

Grc38\_ENST00000617883|miRNA

---

Grc38\_ENST00000619973|miRNA

---

Grc38\_ENST00000621427|miRNA

---

Grc38\_ENST00000621981|miRNA

---

Grc38\_ENST00000623395|lincRNA

---

Grc38\_ENST00000624919|sense\_overlapping

---

Grc38\_ENST00000625614|miRNA

---

Grc38\_ENST00000625656|miRNA

---

Grc38\_ENST00000626018|miRNA

---

Grc38\_ENST00000626279|miRNA

---

Grc38\_ENST00000626443|miRNA

---

Grc38\_ENST00000626841|miRNA

---

Grc38\_ENST00000626896|miRNA

---

Grc38\_ENST00000627736|miRNA

---

Grc38\_ENST00000627889|miRNA

---

Grc38\_ENST00000628052|miRNA

---

Grc38\_ENST00000628701|miRNA

---

Grc38\_ENST00000630014|miRNA

---

Grc38\_ENST00000630551|miRNA

---

Grc38\_ENST00000630620|miRNA

---

Grc38\_ENST00000630786|miRNA

---

Grc38\_ENST00000630788|miRNA

---

lncrnadb\_7sl\_homosapiens\_1|Homo

---

lncrnadb\_mt-lipcar\_hg\_1|Homo

---

lncrnadb\_Y-RNAs\_hg\_1|Homo

---

lncrnadb\_yam-1\_mm10\_1|Mus

---

mirBASE\_bta-miR-2889

---

mirBASE\_hsa-miR-4485-3p

---

piRNA|DQ570940

---

piRNA|DQ570956

---

piRNA|DQ570992

---

piRNA|DQ571031

---

piRNA|DQ571524

---

piRNA|DQ571813

---

piRNA|DQ575656

---

piRNA|DQ575658

---

piRNA|DQ575660

---

piRNA|DQ575882

---

piRNA|DQ575884

---

piRNA|DQ576605

---

piRNA|DQ576872

---

piRNA|DQ576918

---

piRNA|DQ577772

---

piRNA|DQ582264

---

piRNA|DQ582838

---

piRNA|DQ584698

---

piRNA|DQ588594

---

piRNA|DQ590013

---

piRNA|DQ590548

---

piRNA|DQ590835

---

piRNA|DQ592931

---

piRNA|DQ592932

---

piRNA|DQ592953

---

piRNA|DQ593325

---

piRNA|DQ593356

---

piRNA|DQ593358

---

piRNA|DQ593407

---

piRNA|DQ593423

---

piRNA|DQ594453

---

piRNA|DQ594465

---

piRNA|DQ595536

---

piRNA|DQ596538

---

piRNA|DQ596805

---

piRNA|DQ597110

---

piRNA|DQ597215

---

piRNA|DQ597217

---

piRNA|DQ597218

---

piRNA|DQ597341

---

piRNA|DQ597347

---

piRNA|DQ597397

---

piRNA|DQ597403

---

piRNA|DQ597482

---

piRNA|DQ597916

---

piRNA|DQ597971

---

piRNA|DQ597975

---

piRNA|DQ598008

---

piRNA|DQ598167

---

piRNA|DQ598180

---

piRNA|DQ598183

---

piRNA|DQ598252

---

piRNA|DQ598312

---

piRNA|DQ598445

---

piRNA|DQ598639

---

piRNA|DQ598675

---

piRNA|DQ598677

---

piRNA|DQ600952

---

Y\_RNA\_AADD01087475.1/2469-2552

---

Y\_RNA\_AADN03001926.1/468172-468061

---

Y\_RNA\_AAFC03099237.1/8865-8959

---

Y\_RNA\_AAFC03099238.1/121707-121818

---

Y\_RNA\_AAFR03014070.1/16387-16488

---

Y\_RNA\_AAHX01030026.1/7139-7028

---

Y\_RNA\_AAPE02039776.1/102994-103096

---

Y\_RNA\_AAPN01172659.1/53662-53775

---

Y\_RNA\_AAPY01489510.1/220-119

---

Y\_RNA\_AAQR03087348.1/2666-2565

---

Y\_RNA\_AAQR03087350.1/1071-976

---

Y\_RNA\_AAWR02036109.1/33571-33459

---

Y\_RNA\_AAYZ01133134.1/5808-5901

---

Y\_RNA\_ABDC01601884.1/580-486

---

Y\_RNA\_AC073140.5/117955-118049

---

Y\_RNA\_AC190269.3/73147-73248

---

Y\_RNA\_AC191785.1/102750-102844

---

Y\_RNA\_AC193857.1/159000-159109

---

Y\_RNA\_AC205306.6/147779-147685

---

Y\_RNA\_CT104657.1/45296-45390

---

Y\_RNA\_CT104669.1/59726-59616

**Elements in Not Significant in NF-CELL vs NF-EXO and Diff Represented in NF-CELL vs NF-EXO**

**Elements in Not Significant in CAF-CELL vs CAF-EXO and Diff Represented in NF-CELL vs NF-EXO**
  


---

Grc38\_ENST00000315707|lincRNA

---

Grc38\_ENST00000362142|miRNA

---

Grc38\_ENST00000362160|miRNA

---

Grc38\_ENST00000362188|miRNA

---

Grc38\_ENST00000362512|snRNA

---

Grc38\_ENST00000362700|snRNA

---

Grc38\_ENST00000363046|ribozyme

---

Grc38\_ENST00000363062|snRNA

---

Grc38\_ENST00000363497|snoRNA

---

Grc38\_ENST00000363515|snoRNA

---

Grc38\_ENST00000363548|snoRNA

---

Grc38\_ENST00000363572|snoRNA

---

Grc38\_ENST00000363738|snoRNA

---

Grc38\_ENST00000364071|snRNA

---

Grc38\_ENST00000364514|snoRNA

---

Grc38\_ENST00000364687|snoRNA

---

Grc38\_ENST00000364877|snRNA

---

Grc38\_ENST00000365012|snoRNA

---

Grc38\_ENST00000365199|snRNA

---

Grc38\_ENST00000365372|snRNA

---

Grc38\_ENST00000365430|snRNA

---

Grc38\_ENST00000365493|snoRNA

---

Grc38\_ENST00000366365|processed\_transcript

---

Grc38\_ENST00000383886|snRNA

---

Grc38\_ENST00000383893|snoRNA

---

Grc38\_ENST00000383904|snRNA

---

Grc38\_ENST00000384174|snoRNA

---

Grc38\_ENST00000384276|snRNA

---

Grc38\_ENST00000384423|snoRNA

---

Grc38\_ENST00000384436|snoRNA

---

Grc38\_ENST00000384505|snRNA

---

Grc38\_ENST00000384583|snoRNA

---

Grc38\_ENST00000384584|snoRNA

---

Grc38\_ENST00000384674|snoRNA

---

Grc38\_ENST00000384711|snoRNA

---

Grc38\_ENST00000384762|snoRNA

---

Grc38\_ENST00000384835|miRNA

---

Grc38\_ENST00000384836|miRNA

---

Grc38\_ENST00000384868|miRNA

---

Grc38\_ENST00000384886|miRNA

---

Grc38\_ENST00000384979|miRNA

---

Grc38\_ENST00000384996|miRNA

---

Grc38\_ENST00000385059|miRNA

---

Grc38\_ENST00000385140|miRNA

---

Grc38\_ENST00000385221|miRNA

---

Grc38\_ENST00000385242|miRNA

---

Grc38\_ENST00000385278|miRNA

---

Grc38\_ENST00000390194|miRNA

---

Grc38\_ENST00000390751|miRNA

---

Grc38\_ENST00000390781|miRNA

---

Grc38\_ENST00000390806|miRNA

---

Grc38\_ENST00000390833|snoRNA

---

Grc38\_ENST00000391040|snoRNA

---

Grc38\_ENST00000391239|snRNA

---

Grc38\_ENST00000391249|snoRNA

---

Grc38\_ENST00000391305|snoRNA

---

Grc38\_ENST00000401115|miRNA

---

Grc38\_ENST00000401201|miRNA

---

Grc38\_ENST00000401235|miRNA

---

Grc38\_ENST00000401312|miRNA

---

Grc38\_ENST00000401325|miRNA

---

Grc38\_ENST00000401335|miRNA

---

Grc38\_ENST00000401347|miRNA

---

Grc38\_ENST00000408139|snoRNA

---

Grc38\_ENST00000408247|miRNA

---

Grc38\_ENST00000408493|snoRNA

---

Grc38\_ENST00000408625|miRNA

---

Grc38\_ENST00000408746|snoRNA

---

Grc38\_ENST00000408790|miRNA

---

Grc38\_ENST00000410135|snRNA

---

Grc38\_ENST00000410506|snRNA

---

Grc38\_ENST00000410557|snoRNA

---

Grc38\_ENST00000410695|snRNA

---

Grc38\_ENST00000411013|miRNA

---

Grc38\_ENST00000411041|snRNA

---

Grc38\_ENST00000411132|snRNA

---

Grc38\_ENST00000411232|miRNA

---

Grc38\_ENST00000411266|snRNA

---

Grc38\_ENST00000411343|miRNA

---

Grc38\_ENST00000418420|lincRNA

---

Grc38\_ENST00000418574|lincRNA

---

Grc38\_ENST00000420330|lincRNA

---

Grc38\_ENST00000421737|lincRNA

---

Grc38\_ENST00000423515|lincRNA

---

Grc38\_ENST00000426282|lincRNA

---

Grc38\_ENST00000427161|lincRNA

---

Grc38\_ENST00000427831|processed\_transcript

---

Grc38\_ENST00000429124|lincRNA

---

Grc38\_ENST00000429464|lincRNA

---

Grc38\_ENST00000429829|lincRNA

---

Grc38\_ENST00000430429|lincRNA

---

Grc38\_ENST00000430766|lincRNA

---

Grc38\_ENST00000432438|lincRNA

---

Grc38\_ENST00000433510|lincRNA

---

Grc38\_ENST00000433673|lincRNA

---

Grc38\_ENST00000436123|lincRNA

---

Grc38\_ENST00000442712|lincRNA

---

Grc38\_ENST00000446754|lincRNA

---

Grc38\_ENST00000447950|lincRNA

---

Grc38\_ENST00000448958|lincRNA

---

Grc38\_ENST00000452199|lincRNA

---

Grc38\_ENST00000452320|processed\_transcript

---

Grc38\_ENST00000455929|sense\_intronic

---

Grc38\_ENST00000456273|sense\_intronic

---

Grc38\_ENST00000458806|snoRNA

---

Grc38\_ENST00000458896|miRNA

---

Grc38\_ENST00000458922|snoRNA

---

Grc38\_ENST00000459004|snoRNA

---

Grc38\_ENST00000459191|snRNA

---

Grc38\_ENST00000459255|snoRNA

---

Grc38\_ENST00000459491|snRNA

---

Grc38\_ENST00000459585|miRNA

---

Grc38\_ENST00000476964|sense\_overlapping

---

Grc38\_ENST00000480237|processed\_transcript

---

Grc38\_ENST00000484836|processed\_transcript

---

Grc38\_ENST00000484859|processed\_transcript

---

Grc38\_ENST00000488425|lincRNA

---

Grc38\_ENST00000489090|lincRNA

---

Grc38\_ENST00000492337|sense\_overlapping

---

Grc38\_ENST00000498731|sense\_overlapping

---

Grc38\_ENST00000501211|sense\_intronic

---

Grc38\_ENST00000501702|lincRNA

---

Grc38\_ENST00000501855|lincRNA

---

Grc38\_ENST00000502162|lincRNA

---

Grc38\_ENST00000502187|lincRNA

---

Grc38\_ENST00000502221|lincRNA

---

Grc38\_ENST00000503051|lincRNA

---

Grc38\_ENST00000503723|sense\_intronic

---

Grc38\_ENST00000503882|lincRNA

---

Grc38\_ENST00000504082|lincRNA

---

Grc38\_ENST00000507770|sense\_intronic

---

Grc38\_ENST00000508406|processed\_transcript

---

Grc38\_ENST00000513358|lincRNA

---

Grc38\_ENST00000514519|lincRNA

---

Grc38\_ENST00000515329|processed\_transcript

---

Grc38\_ENST00000515924|scaRNA

---

Grc38\_ENST00000515981|scaRNA

---

Grc38\_ENST00000516061|snRNA

---

Grc38\_ENST00000516066|snRNA

---

Grc38\_ENST00000516068|snRNA

---

Grc38\_ENST00000516086|snRNA

---

Grc38\_ENST00000516145|snRNA

---

Grc38\_ENST00000516160|miRNA

---

Grc38\_ENST00000516221|snRNA

---

Grc38\_ENST00000516330|scaRNA

---

Grc38\_ENST00000516387|miRNA

---

Grc38\_ENST00000516403|snRNA

---

Grc38\_ENST00000516405|snRNA

---

Grc38\_ENST00000516461|miRNA

---

Grc38\_ENST00000516479|snRNA

---

Grc38\_ENST00000516581|snRNA

---

Grc38\_ENST00000516775|miRNA

---

Grc38\_ENST00000516827|snRNA

---

Grc38\_ENST00000516903|scaRNA

---

Grc38\_ENST00000516922|miRNA

---

Grc38\_ENST00000517189|snRNA

---

Grc38\_ENST00000517238|snoRNA

---

Grc38\_ENST00000517283|snoRNA

---

Grc38\_ENST00000521725|lincRNA

---

Grc38\_ENST00000522778|lincRNA

---

Grc38\_ENST00000527332|lincRNA

---

Grc38\_ENST00000527550|sense\_overlapping

---

Grc38\_ENST00000540802|processed\_transcript

---

Grc38\_ENST00000542763|lincRNA

---

Grc38\_ENST00000544420|lincRNA

---

Grc38\_ENST00000547717|processed\_transcript

---

Grc38\_ENST00000548266|lincRNA

---

Grc38\_ENST00000553679|lincRNA

---

Grc38\_ENST00000554333|lincRNA

---

Grc38\_ENST00000554926|lincRNA

---

Grc38\_ENST00000555379|processed\_transcript

---

Grc38\_ENST00000556913|lincRNA

---

Grc38\_ENST00000557108|processed\_transcript

---

Grc38\_ENST00000562063|lincRNA

---

Grc38\_ENST00000562284|sense\_overlapping

---

Grc38\_ENST00000562409|sense\_intronic

---

Grc38\_ENST00000562691|sense\_overlapping

---

Grc38\_ENST00000562917|lincRNA

---

Grc38\_ENST00000563931|lincRNA

---

Grc38\_ENST00000565181|lincRNA

---

Grc38\_ENST00000567769|lincRNA

---

Grc38\_ENST00000568885|sense\_intronic

---

Grc38\_ENST00000570843|lincRNA

---

Grc38\_ENST00000571975|sense\_overlapping

---

Grc38\_ENST00000577323|miRNA

---

Grc38\_ENST00000577381|miRNA

---

Grc38\_ENST00000577494|miRNA

---

Grc38\_ENST00000577684|lincRNA

---

Grc38\_ENST00000577845|miRNA

---

Grc38\_ENST00000577955|miRNA

---

Grc38\_ENST00000578152|lincRNA

---

Grc38\_ENST00000578183|snoRNA

---

Grc38\_ENST00000578212|miRNA

---

Grc38\_ENST00000578452|miRNA

---

Grc38\_ENST00000578585|lincRNA

---

Grc38\_ENST00000579046|miRNA

---

Grc38\_ENST00000579227|miRNA

---

Grc38\_ENST00000579264|miRNA

---

Grc38\_ENST00000579622|miRNA

---

Grc38\_ENST00000579798|miRNA

---

Grc38\_ENST00000579890|miRNA

---

Grc38\_ENST00000580664|miRNA

---

Grc38\_ENST00000580761|miRNA

---

Grc38\_ENST00000580862|miRNA

---

Grc38\_ENST00000581170|lincRNA

---

Grc38\_ENST00000581217|miRNA

---

Grc38\_ENST00000581890|miRNA

---

Grc38\_ENST00000582203|miRNA

---

Grc38\_ENST00000582434|miRNA

---

Grc38\_ENST00000582922|miRNA

---

Grc38\_ENST00000583098|miRNA

---

Grc38\_ENST00000583110|miRNA

---

Grc38\_ENST00000583426|sense\_intronic

---

Grc38\_ENST00000583472|miRNA

---

Grc38\_ENST00000583730|miRNA

---

Grc38\_ENST00000584045|miRNA

---

Grc38\_ENST00000584213|snoRNA

---

Grc38\_ENST00000584302|snoRNA

---

Grc38\_ENST00000584345|miRNA

---

Grc38\_ENST00000584820|miRNA

---

Grc38\_ENST00000584899|miRNA

---

Grc38\_ENST00000585011|snoRNA

---

Grc38\_ENST00000585165|miRNA

---

Grc38\_ENST00000585691|lincRNA

---

Grc38\_ENST00000585863|lincRNA

---

Grc38\_ENST00000586026|miRNA

---

Grc38\_ENST00000587762|lincRNA

---

Grc38\_ENST00000590912|lincRNA

---

Grc38\_ENST00000591501|lincRNA

---

Grc38\_ENST00000597619|lincRNA

---

Grc38\_ENST00000599944|lincRNA

---

Grc38\_ENST00000606207|miRNA

---

Grc38\_ENST00000606343|lincRNA

---

Grc38\_ENST00000606393|lincRNA

---

Grc38\_ENST00000606837|miRNA

---

Grc38\_ENST00000607441|miRNA

---

Grc38\_ENST00000607458|lincRNA

---

Grc38\_ENST00000609737|processed\_transcript

---

Grc38\_ENST00000609770|lincRNA

---

Grc38\_ENST00000609879|processed\_transcript

---

Grc38\_ENST00000609937|lincRNA

---

Grc38\_ENST00000609972|lincRNA

---

Grc38\_ENST00000610307|miRNA

---

Grc38\_ENST00000610481|lincRNA

---

Grc38\_ENST00000610762|miRNA

---

Grc38\_ENST00000611316|lincRNA

---

Grc38\_ENST00000612070|miRNA

---

Grc38\_ENST00000612156|lincRNA

---

Grc38\_ENST00000612568|lincRNA

---

Grc38\_ENST00000612707|lincRNA

---

Grc38\_ENST00000612986|sense\_intronic

---

Grc38\_ENST00000614031|miRNA

---

Grc38\_ENST00000616984|sRNA

---

Grc38\_ENST00000617852|miRNA

---

Grc38\_ENST00000618716|miRNA

---

Grc38\_ENST00000618845|lincRNA

---

Grc38\_ENST00000619354|lincRNA

---

Grc38\_ENST00000619431|processed\_transcript

---

Grc38\_ENST00000619524|snRNA

---

Grc38\_ENST00000620577|miRNA

---

Grc38\_ENST00000620778|lincRNA

---

Grc38\_ENST00000621916|miRNA

---

Grc38\_ENST00000622201|sense\_intronic

---

Grc38\_ENST00000622229|lincRNA

---

Grc38\_ENST00000622285|snRNA

---

Grc38\_ENST00000622602|lincRNA

---

Grc38\_ENST00000623111|sense\_overlapping

---

Grc38\_ENST00000623130|sense\_overlapping

---

Grc38\_ENST00000623440|sense\_intronic

---

Grc38\_ENST00000623490|lincRNA

---

Grc38\_ENST00000623644|lincRNA

---

Grc38\_ENST00000623647|processed\_transcript

---

Grc38\_ENST00000624086|processed\_transcript

---

Grc38\_ENST00000624235|lincRNA

---

Grc38\_ENST00000624243|lincRNA

---

Grc38\_ENST00000624278|sense\_intronic

---

Grc38\_ENST00000624350|lincRNA

---

Grc38\_ENST00000624601|lincRNA

---

Grc38\_ENST00000625157|lincRNA

---

Grc38\_ENST00000625347|miRNA

---

Grc38\_ENST00000625513|lincRNA

---

Grc38\_ENST00000625525|miRNA

---

Grc38\_ENST00000625643|scaRNA

---

Grc38\_ENST00000626287|lincRNA

---

Grc38\_ENST00000626759|miRNA

---

Grc38\_ENST00000627128|miRNA

---

Grc38\_ENST00000627173|lincRNA

---

Grc38\_ENST00000628590|snoRNA

---

Grc38\_ENST00000628595|processed\_transcript

---

Grc38\_ENST00000628661|miRNA

---

Grc38\_ENST00000628724|miRNA

---

Grc38\_ENST00000629107|miRNA

---

Grc38\_ENST00000629173|miRNA

---

Grc38\_ENST00000629282|miRNA

---

Grc38\_ENST00000629284|miRNA

---

Grc38\_ENST00000629902|sense\_intronic

---

Grc38\_ENST00000629969|lincRNA

---

Grc38\_ENST00000630292|miRNA

---

Grc38\_ENST00000630372|miRNA

---

Grc38\_ENST00000630515|miRNA

---

Grc38\_ENST00000630688|snRNA

---

Grc38\_ENST00000630692|miRNA

---

Grc38\_ENST00000630984|miRNA

---

Grc38\_ENST00000631258|lincRNA

---

lncrnadb\_megamind\_musmusculus\_5|Mus

---

mirBASE\_mmT-miR-5100

---

piRNA|DQ570344

---

piRNA|DQ570968

---

piRNA|DQ571367

---

piRNA|DQ571550

---

piRNA|DQ571823

---

piRNA|DQ572175

---

piRNA|DQ573178

---

piRNA|DQ573323

---

piRNA|DQ576604

---

piRNA|DQ576880

---

piRNA|DQ578315

---

piRNA|DQ578783

---

piRNA|DQ579858

---

piRNA|DQ580509

---

piRNA|DQ580639

---

piRNA|DQ580854

---

piRNA|DQ582243

---

piRNA|DQ584149

---

piRNA|DQ584318

---

piRNA|DQ585088

---

piRNA|DQ585435

---

piRNA|DQ587619

---

piRNA|DQ588628

---

piRNA|DQ590448

---

piRNA|DQ592265

---

piRNA|DQ596455

---

piRNA|DQ598175

---

piRNA|DQ598806

---

piRNA|DQ598921

---

piRNA|DQ598942

---

piRNA|DQ600890

---

piRNA|DQ601938

---

Y\_RNA\_AABR05113607.1/617-718

---

Y\_RNA\_AANT01114500.1/1882-1983

---

Y\_RNA\_ABDC01212300.1/2415-2498

---

Y\_RNA\_AC199101.3/202469-202368

**Elements in Not Significant in NF-CELL vs NF-EXO, Not Significant in CAF-CELL vs CAF-EXO and Diff Represented in NF-CELL vs NF-EXO**

**Elements in Diff Represented in CAF-CELL vs CAF-EXO, Not Significant in NF-CELL vs NF-EXO and Diff Represented in NF-CELL vs NF-EXO**

**Elements in Diff Represented in CAF-CELL vs CAF-EXO, Not Significant in CAF-CELL vs CAF-EXO and Diff Represented in NF-CELL vs NF-EXO**

Diff Represented in   
CAF-CELL vs CAF-EXO

Not Significant in   
NF-CELL vs NF-EXO

Not Significant in   
CAF-CELL vs CAF-EXO

Diff Represented in   
NF-CELL vs NF-EXO
