# Supplementary material for: Differential distribution and enrichment of non-coding RNAs in exosomes from normal and Cancer-associated fibroblasts in colorectal cancer
Source: Mol Cancer. 2018 Aug 3;17:114. doi: 10.1186/s12943-018-0863-4 (PMC6091058; doi:10.1186/s12943-018-0863-4)
Supplement: Supplementary file 8 — Mini web site presenting a dynamic venn diagram showing the relationships between the results cellular or exosomal over represented in the two analyses performed between NF- and CAF- exosomes. Clicking on any intersected number, the web site opens a dialog summarizing the ncRNAs species that correspond to the intersection. (ZIP 354 kb) [file 12943_2018_863_MOESM8_ESM.zip › Additional File 8/venn.html]

## Venn diagram

This script creates venn diagrams of up to 5 samples using up to 5 csv files as arguments. This is, to create a 2-samples venn diagram you
must to upload 2 csvs, to a create a 4-samples venn diagram upload 4 csvs and so on. Each csv must contain a list with the items to compare
among samples.

### Requirements

Here you have an example of zip input accepted by the script.

### Parameters

Input column separator
; (semicolon)
"\t" (tab)
, (comma)
" " (space)

### Run the analysis

Upload the input csv and click on RUN button the script will return you a zip file containing the results.
  
  

  
  

  
  

  
  

  
  

  
  

  
  

Download


40

274

95

68

2

519

172

**Elements in Over expressed in CAF-EXO**
  


---

Grc38\_ENST00000339037|lincRNA

---

Grc38\_ENST00000363314|snRNA

---

Grc38\_ENST00000363327|snRNA

---

Grc38\_ENST00000383902|snRNA

---

Grc38\_ENST00000384181|snRNA

---

Grc38\_ENST00000384328|snRNA

---

Grc38\_ENST00000384472|snRNA

---

Grc38\_ENST00000384476|snRNA

---

Grc38\_ENST00000384610|snRNA

---

Grc38\_ENST00000384770|snRNA

---

Grc38\_ENST00000411247|snRNA

---

Grc38\_ENST00000441255|lincRNA

---

Grc38\_ENST00000457339|lincRNA

---

Grc38\_ENST00000508096|sense\_overlapping

---

Grc38\_ENST00000516337|miRNA

---

Grc38\_ENST00000516502|snRNA

---

Grc38\_ENST00000516659|snRNA

---

Grc38\_ENST00000517228|miRNA

---

Grc38\_ENST00000517280|snRNA

---

Grc38\_ENST00000528381|lincRNA

---

Grc38\_ENST00000529733|lincRNA

---

Grc38\_ENST00000550723|lincRNA

---

Grc38\_ENST00000562127|lincRNA

---

Grc38\_ENST00000579051|miRNA

---

Grc38\_ENST00000584185|miRNA

---

Grc38\_ENST00000602813|lincRNA

---

Grc38\_ENST00000606801|snRNA

---

Grc38\_ENST00000612638|miRNA

---

Grc38\_ENST00000613023|snRNA

---

Grc38\_ENST00000614084|miRNA

---

Grc38\_ENST00000614774|snRNA

---

Grc38\_ENST00000617733|miRNA

---

Grc38\_ENST00000619109|snRNA

---

Grc38\_ENST00000623027|lincRNA

---

Grc38\_ENST00000628107|miRNA

---

Grc38\_ENST00000630744|miRNA

---

piRNA|DQ572769

---

piRNA|DQ590139

---

piRNA|DQ597968

---

piRNA|DQ599478

**Elements in Over expressed in NF-CELL**
  


---

Grc38\_ENST00000362188|miRNA

---

Grc38\_ENST00000362700|snRNA

---

Grc38\_ENST00000363046|ribozyme

---

Grc38\_ENST00000363062|snRNA

---

Grc38\_ENST00000363497|snoRNA

---

Grc38\_ENST00000363515|snoRNA

---

Grc38\_ENST00000363548|snoRNA

---

Grc38\_ENST00000363572|snoRNA

---

Grc38\_ENST00000363738|snoRNA

---

Grc38\_ENST00000364071|snRNA

---

Grc38\_ENST00000364514|snoRNA

---

Grc38\_ENST00000364687|snoRNA

---

Grc38\_ENST00000364877|snRNA

---

Grc38\_ENST00000365012|snoRNA

---

Grc38\_ENST00000365199|snRNA

---

Grc38\_ENST00000365372|snRNA

---

Grc38\_ENST00000365430|snRNA

---

Grc38\_ENST00000365493|snoRNA

---

Grc38\_ENST00000366365|processed\_transcript

---

Grc38\_ENST00000383886|snRNA

---

Grc38\_ENST00000383893|snoRNA

---

Grc38\_ENST00000383904|snRNA

---

Grc38\_ENST00000384174|snoRNA

---

Grc38\_ENST00000384276|snRNA

---

Grc38\_ENST00000384423|snoRNA

---

Grc38\_ENST00000384436|snoRNA

---

Grc38\_ENST00000384505|snRNA

---

Grc38\_ENST00000384583|snoRNA

---

Grc38\_ENST00000384584|snoRNA

---

Grc38\_ENST00000384674|snoRNA

---

Grc38\_ENST00000384711|snoRNA

---

Grc38\_ENST00000384762|snoRNA

---

Grc38\_ENST00000384868|miRNA

---

Grc38\_ENST00000384979|miRNA

---

Grc38\_ENST00000384996|miRNA

---

Grc38\_ENST00000385221|miRNA

---

Grc38\_ENST00000385242|miRNA

---

Grc38\_ENST00000385278|miRNA

---

Grc38\_ENST00000390194|miRNA

---

Grc38\_ENST00000390781|miRNA

---

Grc38\_ENST00000390806|miRNA

---

Grc38\_ENST00000390833|snoRNA

---

Grc38\_ENST00000391040|snoRNA

---

Grc38\_ENST00000391239|snRNA

---

Grc38\_ENST00000391249|snoRNA

---

Grc38\_ENST00000391305|snoRNA

---

Grc38\_ENST00000401115|miRNA

---

Grc38\_ENST00000401201|miRNA

---

Grc38\_ENST00000401235|miRNA

---

Grc38\_ENST00000401312|miRNA

---

Grc38\_ENST00000401325|miRNA

---

Grc38\_ENST00000401335|miRNA

---

Grc38\_ENST00000401347|miRNA

---

Grc38\_ENST00000408139|snoRNA

---

Grc38\_ENST00000408247|miRNA

---

Grc38\_ENST00000408493|snoRNA

---

Grc38\_ENST00000408625|miRNA

---

Grc38\_ENST00000408746|snoRNA

---

Grc38\_ENST00000408790|miRNA

---

Grc38\_ENST00000410135|snRNA

---

Grc38\_ENST00000410506|snRNA

---

Grc38\_ENST00000410557|snoRNA

---

Grc38\_ENST00000411013|miRNA

---

Grc38\_ENST00000411041|snRNA

---

Grc38\_ENST00000411132|snRNA

---

Grc38\_ENST00000411232|miRNA

---

Grc38\_ENST00000411266|snRNA

---

Grc38\_ENST00000411343|miRNA

---

Grc38\_ENST00000418420|lincRNA

---

Grc38\_ENST00000418574|lincRNA

---

Grc38\_ENST00000420330|lincRNA

---

Grc38\_ENST00000421737|lincRNA

---

Grc38\_ENST00000423515|lincRNA

---

Grc38\_ENST00000426282|lincRNA

---

Grc38\_ENST00000427161|lincRNA

---

Grc38\_ENST00000427831|processed\_transcript

---

Grc38\_ENST00000429124|lincRNA

---

Grc38\_ENST00000429464|lincRNA

---

Grc38\_ENST00000429829|lincRNA

---

Grc38\_ENST00000430429|lincRNA

---

Grc38\_ENST00000430766|lincRNA

---

Grc38\_ENST00000432438|lincRNA

---

Grc38\_ENST00000433510|lincRNA

---

Grc38\_ENST00000433673|lincRNA

---

Grc38\_ENST00000442712|lincRNA

---

Grc38\_ENST00000446754|lincRNA

---

Grc38\_ENST00000447950|lincRNA

---

Grc38\_ENST00000452320|processed\_transcript

---

Grc38\_ENST00000455929|sense\_intronic

---

Grc38\_ENST00000456273|sense\_intronic

---

Grc38\_ENST00000458806|snoRNA

---

Grc38\_ENST00000458922|snoRNA

---

Grc38\_ENST00000459004|snoRNA

---

Grc38\_ENST00000459191|snRNA

---

Grc38\_ENST00000459255|snoRNA

---

Grc38\_ENST00000459491|snRNA

---

Grc38\_ENST00000459585|miRNA

---

Grc38\_ENST00000476964|sense\_overlapping

---

Grc38\_ENST00000480237|processed\_transcript

---

Grc38\_ENST00000484836|processed\_transcript

---

Grc38\_ENST00000484859|processed\_transcript

---

Grc38\_ENST00000489090|lincRNA

---

Grc38\_ENST00000492337|sense\_overlapping

---

Grc38\_ENST00000498731|sense\_overlapping

---

Grc38\_ENST00000501211|sense\_intronic

---

Grc38\_ENST00000502187|lincRNA

---

Grc38\_ENST00000502221|lincRNA

---

Grc38\_ENST00000503723|sense\_intronic

---

Grc38\_ENST00000503882|lincRNA

---

Grc38\_ENST00000507770|sense\_intronic

---

Grc38\_ENST00000515329|processed\_transcript

---

Grc38\_ENST00000515924|scaRNA

---

Grc38\_ENST00000515981|scaRNA

---

Grc38\_ENST00000516061|snRNA

---

Grc38\_ENST00000516066|snRNA

---

Grc38\_ENST00000516068|snRNA

---

Grc38\_ENST00000516086|snRNA

---

Grc38\_ENST00000516145|snRNA

---

Grc38\_ENST00000516160|miRNA

---

Grc38\_ENST00000516221|snRNA

---

Grc38\_ENST00000516330|scaRNA

---

Grc38\_ENST00000516387|miRNA

---

Grc38\_ENST00000516403|snRNA

---

Grc38\_ENST00000516405|snRNA

---

Grc38\_ENST00000516479|snRNA

---

Grc38\_ENST00000516581|snRNA

---

Grc38\_ENST00000516827|snRNA

---

Grc38\_ENST00000516903|scaRNA

---

Grc38\_ENST00000516922|miRNA

---

Grc38\_ENST00000517189|snRNA

---

Grc38\_ENST00000517238|snoRNA

---

Grc38\_ENST00000517283|snoRNA

---

Grc38\_ENST00000522778|lincRNA

---

Grc38\_ENST00000527332|lincRNA

---

Grc38\_ENST00000527550|sense\_overlapping

---

Grc38\_ENST00000540802|processed\_transcript

---

Grc38\_ENST00000542763|lincRNA

---

Grc38\_ENST00000553679|lincRNA

---

Grc38\_ENST00000554333|lincRNA

---

Grc38\_ENST00000554926|lincRNA

---

Grc38\_ENST00000555379|processed\_transcript

---

Grc38\_ENST00000556913|lincRNA

---

Grc38\_ENST00000557108|processed\_transcript

---

Grc38\_ENST00000562063|lincRNA

---

Grc38\_ENST00000562284|sense\_overlapping

---

Grc38\_ENST00000562409|sense\_intronic

---

Grc38\_ENST00000562691|sense\_overlapping

---

Grc38\_ENST00000562917|lincRNA

---

Grc38\_ENST00000563931|lincRNA

---

Grc38\_ENST00000567769|lincRNA

---

Grc38\_ENST00000568885|sense\_intronic

---

Grc38\_ENST00000571975|sense\_overlapping

---

Grc38\_ENST00000577381|miRNA

---

Grc38\_ENST00000577494|miRNA

---

Grc38\_ENST00000577845|miRNA

---

Grc38\_ENST00000577955|miRNA

---

Grc38\_ENST00000578152|lincRNA

---

Grc38\_ENST00000578183|snoRNA

---

Grc38\_ENST00000578212|miRNA

---

Grc38\_ENST00000578452|miRNA

---

Grc38\_ENST00000578585|lincRNA

---

Grc38\_ENST00000579046|miRNA

---

Grc38\_ENST00000579227|miRNA

---

Grc38\_ENST00000579622|miRNA

---

Grc38\_ENST00000579798|miRNA

---

Grc38\_ENST00000579890|miRNA

---

Grc38\_ENST00000580664|miRNA

---

Grc38\_ENST00000581217|miRNA

---

Grc38\_ENST00000581890|miRNA

---

Grc38\_ENST00000582203|miRNA

---

Grc38\_ENST00000582434|miRNA

---

Grc38\_ENST00000582922|miRNA

---

Grc38\_ENST00000583098|miRNA

---

Grc38\_ENST00000583110|miRNA

---

Grc38\_ENST00000583426|sense\_intronic

---

Grc38\_ENST00000583472|miRNA

---

Grc38\_ENST00000583730|miRNA

---

Grc38\_ENST00000584045|miRNA

---

Grc38\_ENST00000584213|snoRNA

---

Grc38\_ENST00000584302|snoRNA

---

Grc38\_ENST00000584820|miRNA

---

Grc38\_ENST00000584899|miRNA

---

Grc38\_ENST00000585011|snoRNA

---

Grc38\_ENST00000585165|miRNA

---

Grc38\_ENST00000585691|lincRNA

---

Grc38\_ENST00000585863|lincRNA

---

Grc38\_ENST00000586026|miRNA

---

Grc38\_ENST00000587762|lincRNA

---

Grc38\_ENST00000590912|lincRNA

---

Grc38\_ENST00000591501|lincRNA

---

Grc38\_ENST00000597619|lincRNA

---

Grc38\_ENST00000606343|lincRNA

---

Grc38\_ENST00000606393|lincRNA

---

Grc38\_ENST00000607441|miRNA

---

Grc38\_ENST00000607458|lincRNA

---

Grc38\_ENST00000609737|processed\_transcript

---

Grc38\_ENST00000609770|lincRNA

---

Grc38\_ENST00000609879|processed\_transcript

---

Grc38\_ENST00000609937|lincRNA

---

Grc38\_ENST00000609972|lincRNA

---

Grc38\_ENST00000610307|miRNA

---

Grc38\_ENST00000610481|lincRNA

---

Grc38\_ENST00000610762|miRNA

---

Grc38\_ENST00000611316|lincRNA

---

Grc38\_ENST00000612070|miRNA

---

Grc38\_ENST00000612156|lincRNA

---

Grc38\_ENST00000612568|lincRNA

---

Grc38\_ENST00000612707|lincRNA

---

Grc38\_ENST00000612986|sense\_intronic

---

Grc38\_ENST00000614031|miRNA

---

Grc38\_ENST00000617852|miRNA

---

Grc38\_ENST00000618716|miRNA

---

Grc38\_ENST00000618845|lincRNA

---

Grc38\_ENST00000619354|lincRNA

---

Grc38\_ENST00000619524|snRNA

---

Grc38\_ENST00000620577|miRNA

---

Grc38\_ENST00000620778|lincRNA

---

Grc38\_ENST00000621916|miRNA

---

Grc38\_ENST00000622229|lincRNA

---

Grc38\_ENST00000622285|snRNA

---

Grc38\_ENST00000623111|sense\_overlapping

---

Grc38\_ENST00000623130|sense\_overlapping

---

Grc38\_ENST00000623440|sense\_intronic

---

Grc38\_ENST00000623490|lincRNA

---

Grc38\_ENST00000623644|lincRNA

---

Grc38\_ENST00000623647|processed\_transcript

---

Grc38\_ENST00000624086|processed\_transcript

---

Grc38\_ENST00000624235|lincRNA

---

Grc38\_ENST00000624243|lincRNA

---

Grc38\_ENST00000624278|sense\_intronic

---

Grc38\_ENST00000624350|lincRNA

---

Grc38\_ENST00000624601|lincRNA

---

Grc38\_ENST00000625157|lincRNA

---

Grc38\_ENST00000625513|lincRNA

---

Grc38\_ENST00000625525|miRNA

---

Grc38\_ENST00000625643|scaRNA

---

Grc38\_ENST00000626287|lincRNA

---

Grc38\_ENST00000626759|miRNA

---

Grc38\_ENST00000627128|miRNA

---

Grc38\_ENST00000627173|lincRNA

---

Grc38\_ENST00000628590|snoRNA

---

Grc38\_ENST00000628595|processed\_transcript

---

Grc38\_ENST00000628661|miRNA

---

Grc38\_ENST00000629107|miRNA

---

Grc38\_ENST00000629173|miRNA

---

Grc38\_ENST00000629282|miRNA

---

Grc38\_ENST00000629284|miRNA

---

Grc38\_ENST00000630292|miRNA

---

Grc38\_ENST00000630372|miRNA

---

Grc38\_ENST00000630515|miRNA

---

Grc38\_ENST00000630688|snRNA

---

Grc38\_ENST00000630692|miRNA

---

Grc38\_ENST00000631258|lincRNA

---

lncrnadb\_megamind\_musmusculus\_5|Mus

---

piRNA|DQ571367

---

piRNA|DQ572175

---

piRNA|DQ573178

---

piRNA|DQ578315

---

piRNA|DQ579858

---

piRNA|DQ580509

---

piRNA|DQ580639

---

piRNA|DQ582243

---

piRNA|DQ584149

---

piRNA|DQ584318

---

piRNA|DQ585435

---

piRNA|DQ587619

---

piRNA|DQ588628

---

piRNA|DQ590448

---

piRNA|DQ592265

---

piRNA|DQ598806

---

piRNA|DQ598921

---

piRNA|DQ598942

---

piRNA|DQ600890

---

piRNA|DQ601938

**Elements in Over expresseed in CAF-CELL**
  


---

Grc38\_ENST00000362104|miRNA

---

Grc38\_ENST00000362105|miRNA

---

Grc38\_ENST00000362116|miRNA

---

Grc38\_ENST00000362125|miRNA

---

Grc38\_ENST00000362134|miRNA

---

Grc38\_ENST00000362147|miRNA

---

Grc38\_ENST00000362162|miRNA

---

Grc38\_ENST00000362173|miRNA

---

Grc38\_ENST00000362195|miRNA

---

Grc38\_ENST00000362215|miRNA

---

Grc38\_ENST00000362218|miRNA

---

Grc38\_ENST00000362227|miRNA

---

Grc38\_ENST00000362263|miRNA

---

Grc38\_ENST00000362265|miRNA

---

Grc38\_ENST00000362283|miRNA

---

Grc38\_ENST00000362295|miRNA

---

Grc38\_ENST00000362298|miRNA

---

Grc38\_ENST00000362317|miRNA

---

Grc38\_ENST00000364699|snoRNA

---

Grc38\_ENST00000364995|snoRNA

---

Grc38\_ENST00000365731|miRNA

---

Grc38\_ENST00000384121|snRNA

---

Grc38\_ENST00000384335|snoRNA

---

Grc38\_ENST00000384384|snoRNA

---

Grc38\_ENST00000384679|snoRNA

---

Grc38\_ENST00000384853|miRNA

---

Grc38\_ENST00000384863|miRNA

---

Grc38\_ENST00000384871|miRNA

---

Grc38\_ENST00000384879|miRNA

---

Grc38\_ENST00000384881|miRNA

---

Grc38\_ENST00000384889|miRNA

---

Grc38\_ENST00000384898|miRNA

---

Grc38\_ENST00000384901|miRNA

---

Grc38\_ENST00000384906|miRNA

---

Grc38\_ENST00000384915|miRNA

---

Grc38\_ENST00000384918|miRNA

---

Grc38\_ENST00000384965|miRNA

---

Grc38\_ENST00000384970|miRNA

---

Grc38\_ENST00000384992|miRNA

---

Grc38\_ENST00000385004|miRNA

---

Grc38\_ENST00000385009|miRNA

---

Grc38\_ENST00000385011|miRNA

---

Grc38\_ENST00000385015|miRNA

---

Grc38\_ENST00000385025|miRNA

---

Grc38\_ENST00000385026|miRNA

---

Grc38\_ENST00000385043|miRNA

---

Grc38\_ENST00000385050|miRNA

---

Grc38\_ENST00000385055|miRNA

---

Grc38\_ENST00000385060|miRNA

---

Grc38\_ENST00000385077|miRNA

---

Grc38\_ENST00000385199|miRNA

---

Grc38\_ENST00000385209|miRNA

---

Grc38\_ENST00000385223|miRNA

---

Grc38\_ENST00000385230|miRNA

---

Grc38\_ENST00000385231|miRNA

---

Grc38\_ENST00000385240|miRNA

---

Grc38\_ENST00000385258|miRNA

---

Grc38\_ENST00000385299|miRNA

---

Grc38\_ENST00000390180|miRNA

---

Grc38\_ENST00000401182|miRNA

---

Grc38\_ENST00000408492|miRNA

---

Grc38\_ENST00000408687|miRNA

---

Grc38\_ENST00000411509|lincRNA

---

Grc38\_ENST00000419813|lincRNA

---

Grc38\_ENST00000454224|lincRNA

---

Grc38\_ENST00000458220|processed\_transcript

---

Grc38\_ENST00000459126|snoRNA

---

Grc38\_ENST00000515031|lincRNA

---

Grc38\_ENST00000521276|miRNA

---

Grc38\_ENST00000532619|lincRNA

---

Grc38\_ENST00000550268|lincRNA

---

Grc38\_ENST00000551631|processed\_transcript

---

Grc38\_ENST00000559298|lincRNA

---

Grc38\_ENST00000578242|miRNA

---

Grc38\_ENST00000578311|miRNA

---

Grc38\_ENST00000579844|miRNA

---

Grc38\_ENST00000579969|snoRNA

---

Grc38\_ENST00000583823|snoRNA

---

Grc38\_ENST00000584034|miRNA

---

Grc38\_ENST00000585078|snoRNA

---

Grc38\_ENST00000597780|sense\_intronic

---

Grc38\_ENST00000606229|sense\_intronic

---

Grc38\_ENST00000606526|snoRNA

---

Grc38\_ENST00000606724|miRNA

---

Grc38\_ENST00000608229|lincRNA

---

Grc38\_ENST00000611454|snRNA

---

Grc38\_ENST00000613376|lincRNA

---

Grc38\_ENST00000617791|lincRNA

---

Grc38\_ENST00000623290|lincRNA

---

Grc38\_ENST00000624184|sense\_overlapping

---

Grc38\_ENST00000627071|lincRNA

---

Grc38\_ENST00000629919|miRNA

---

mirBASE\_sha-miR-21

---

piRNA|DQ582566

---

piRNA|DQ594740

**Elements in Over expressed in NF-EXO**
  


---

Grc38\_ENST00000315707|lincRNA

---

Grc38\_ENST00000362142|miRNA

---

Grc38\_ENST00000362160|miRNA

---

Grc38\_ENST00000362512|snRNA

---

Grc38\_ENST00000384835|miRNA

---

Grc38\_ENST00000384836|miRNA

---

Grc38\_ENST00000384886|miRNA

---

Grc38\_ENST00000385059|miRNA

---

Grc38\_ENST00000385140|miRNA

---

Grc38\_ENST00000390751|miRNA

---

Grc38\_ENST00000410695|snRNA

---

Grc38\_ENST00000436123|lincRNA

---

Grc38\_ENST00000448958|lincRNA

---

Grc38\_ENST00000452199|lincRNA

---

Grc38\_ENST00000458896|miRNA

---

Grc38\_ENST00000488425|lincRNA

---

Grc38\_ENST00000501702|lincRNA

---

Grc38\_ENST00000501855|lincRNA

---

Grc38\_ENST00000502162|lincRNA

---

Grc38\_ENST00000503051|lincRNA

---

Grc38\_ENST00000504082|lincRNA

---

Grc38\_ENST00000508406|processed\_transcript

---

Grc38\_ENST00000513358|lincRNA

---

Grc38\_ENST00000514519|lincRNA

---

Grc38\_ENST00000516461|miRNA

---

Grc38\_ENST00000516775|miRNA

---

Grc38\_ENST00000521725|lincRNA

---

Grc38\_ENST00000544420|lincRNA

---

Grc38\_ENST00000547717|processed\_transcript

---

Grc38\_ENST00000548266|lincRNA

---

Grc38\_ENST00000565181|lincRNA

---

Grc38\_ENST00000570843|lincRNA

---

Grc38\_ENST00000577323|miRNA

---

Grc38\_ENST00000577684|lincRNA

---

Grc38\_ENST00000579264|miRNA

---

Grc38\_ENST00000580761|miRNA

---

Grc38\_ENST00000580862|miRNA

---

Grc38\_ENST00000581170|lincRNA

---

Grc38\_ENST00000584345|miRNA

---

Grc38\_ENST00000599944|lincRNA

---

Grc38\_ENST00000606207|miRNA

---

Grc38\_ENST00000606837|miRNA

---

Grc38\_ENST00000616984|sRNA

---

Grc38\_ENST00000619431|processed\_transcript

---

Grc38\_ENST00000622201|sense\_intronic

---

Grc38\_ENST00000622602|lincRNA

---

Grc38\_ENST00000625347|miRNA

---

Grc38\_ENST00000628724|miRNA

---

Grc38\_ENST00000629902|sense\_intronic

---

Grc38\_ENST00000629969|lincRNA

---

Grc38\_ENST00000630984|miRNA

---

mirBASE\_mmT-miR-5100

---

piRNA|DQ570344

---

piRNA|DQ570968

---

piRNA|DQ571550

---

piRNA|DQ571823

---

piRNA|DQ573323

---

piRNA|DQ576604

---

piRNA|DQ576880

---

piRNA|DQ578783

---

piRNA|DQ580854

---

piRNA|DQ585088

---

piRNA|DQ596455

---

piRNA|DQ598175

---

Y\_RNA\_AABR05113607.1/617-718

---

Y\_RNA\_AANT01114500.1/1882-1983

---

Y\_RNA\_ABDC01212300.1/2415-2498

---

Y\_RNA\_AC199101.3/202469-202368

**Elements in Over expressed in CAF-EXO and Over expressed in NF-CELL**
  


---

Grc38\_ENST00000384619|snRNA

---

Grc38\_ENST00000605806|snRNA

**Elements in Over expressed in CAF-EXO and Over expresseed in CAF-CELL**

**Elements in Over expressed in NF-CELL and Over expresseed in CAF-CELL**
  


---

Grc38\_ENST00000229465|lincRNA

---

Grc38\_ENST00000313495|lincRNA

---

Grc38\_ENST00000349529|miRNA

---

Grc38\_ENST00000356047|lincRNA

---

Grc38\_ENST00000362102|miRNA

---

Grc38\_ENST00000362111|miRNA

---

Grc38\_ENST00000362114|miRNA

---

Grc38\_ENST00000362117|miRNA

---

Grc38\_ENST00000362135|miRNA

---

Grc38\_ENST00000362145|miRNA

---

Grc38\_ENST00000362150|miRNA

---

Grc38\_ENST00000362153|miRNA

---

Grc38\_ENST00000362155|miRNA

---

Grc38\_ENST00000362159|miRNA

---

Grc38\_ENST00000362165|miRNA

---

Grc38\_ENST00000362181|miRNA

---

Grc38\_ENST00000362183|miRNA

---

Grc38\_ENST00000362202|miRNA

---

Grc38\_ENST00000362205|miRNA

---

Grc38\_ENST00000362222|miRNA

---

Grc38\_ENST00000362224|miRNA

---

Grc38\_ENST00000362239|miRNA

---

Grc38\_ENST00000362251|miRNA

---

Grc38\_ENST00000362252|miRNA

---

Grc38\_ENST00000362260|miRNA

---

Grc38\_ENST00000362262|miRNA

---

Grc38\_ENST00000362279|miRNA

---

Grc38\_ENST00000362280|miRNA

---

Grc38\_ENST00000362281|miRNA

---

Grc38\_ENST00000362302|miRNA

---

Grc38\_ENST00000362307|miRNA

---

Grc38\_ENST00000362309|miRNA

---

Grc38\_ENST00000362310|miRNA

---

Grc38\_ENST00000362412|snoRNA

---

Grc38\_ENST00000362443|snRNA

---

Grc38\_ENST00000362477|snRNA

---

Grc38\_ENST00000362507|snRNA

---

Grc38\_ENST00000362607|snoRNA

---

Grc38\_ENST00000362698|snRNA

---

Grc38\_ENST00000362704|snoRNA

---

Grc38\_ENST00000362705|snoRNA

---

Grc38\_ENST00000362723|snoRNA

---

Grc38\_ENST00000362761|snoRNA

---

Grc38\_ENST00000362803|snoRNA

---

Grc38\_ENST00000362805|snoRNA

---

Grc38\_ENST00000362883|snoRNA

---

Grc38\_ENST00000363064|snoRNA

---

Grc38\_ENST00000363091|snoRNA

---

Grc38\_ENST00000363202|snoRNA

---

Grc38\_ENST00000363214|snoRNA

---

Grc38\_ENST00000363217|snoRNA

---

Grc38\_ENST00000363286|snRNA

---

Grc38\_ENST00000363299|snRNA

---

Grc38\_ENST00000363315|snoRNA

---

Grc38\_ENST00000363345|snoRNA

---

Grc38\_ENST00000363389|snoRNA

---

Grc38\_ENST00000363450|snoRNA

---

Grc38\_ENST00000363485|snoRNA

---

Grc38\_ENST00000363536|snoRNA

---

Grc38\_ENST00000363543|snoRNA

---

Grc38\_ENST00000363593|snoRNA

---

Grc38\_ENST00000363610|snoRNA

---

Grc38\_ENST00000363626|snoRNA

---

Grc38\_ENST00000363660|snoRNA

---

Grc38\_ENST00000363664|snoRNA

---

Grc38\_ENST00000363742|snoRNA

---

Grc38\_ENST00000363753|snoRNA

---

Grc38\_ENST00000363836|snoRNA

---

Grc38\_ENST00000363925|snRNA

---

Grc38\_ENST00000363981|snoRNA

---

Grc38\_ENST00000364009|snoRNA

---

Grc38\_ENST00000364027|snoRNA

---

Grc38\_ENST00000364043|snoRNA

---

Grc38\_ENST00000364089|snoRNA

---

Grc38\_ENST00000364113|snoRNA

---

Grc38\_ENST00000364139|snoRNA

---

Grc38\_ENST00000364259|snoRNA

---

Grc38\_ENST00000364294|snRNA

---

Grc38\_ENST00000364310|snRNA

---

Grc38\_ENST00000364370|snoRNA

---

Grc38\_ENST00000364432|snoRNA

---

Grc38\_ENST00000364533|snoRNA

---

Grc38\_ENST00000364569|snRNA

---

Grc38\_ENST00000364578|snoRNA

---

Grc38\_ENST00000364617|snoRNA

---

Grc38\_ENST00000364773|snoRNA

---

Grc38\_ENST00000364802|snoRNA

---

Grc38\_ENST00000364805|snoRNA

---

Grc38\_ENST00000364849|snoRNA

---

Grc38\_ENST00000364931|snRNA

---

Grc38\_ENST00000364938|snoRNA

---

Grc38\_ENST00000364953|snoRNA

---

Grc38\_ENST00000364969|snoRNA

---

Grc38\_ENST00000364977|snoRNA

---

Grc38\_ENST00000365028|snoRNA

---

Grc38\_ENST00000365080|snoRNA

---

Grc38\_ENST00000365128|snoRNA

---

Grc38\_ENST00000365153|snoRNA

---

Grc38\_ENST00000365161|snoRNA

---

Grc38\_ENST00000365172|snoRNA

---

Grc38\_ENST00000365178|snoRNA

---

Grc38\_ENST00000365223|snoRNA

---

Grc38\_ENST00000365382|snoRNA

---

Grc38\_ENST00000365400|snoRNA

---

Grc38\_ENST00000365423|snoRNA

---

Grc38\_ENST00000365444|snoRNA

---

Grc38\_ENST00000365530|snoRNA

---

Grc38\_ENST00000365574|snRNA

---

Grc38\_ENST00000365607|snoRNA

---

Grc38\_ENST00000365633|snoRNA

---

Grc38\_ENST00000365659|snoRNA

---

Grc38\_ENST00000365668|snRNA

---

Grc38\_ENST00000365699|miRNA

---

Grc38\_ENST00000383860|snRNA

---

Grc38\_ENST00000383870|snoRNA

---

Grc38\_ENST00000383875|snoRNA

---

Grc38\_ENST00000383885|snoRNA

---

Grc38\_ENST00000383903|snoRNA

---

Grc38\_ENST00000383953|snoRNA

---

Grc38\_ENST00000384027|snoRNA

---

Grc38\_ENST00000384033|snoRNA

---

Grc38\_ENST00000384048|snoRNA

---

Grc38\_ENST00000384084|snoRNA

---

Grc38\_ENST00000384096|snoRNA

---

Grc38\_ENST00000384136|snRNA

---

Grc38\_ENST00000384147|snoRNA

---

Grc38\_ENST00000384158|snoRNA

---

Grc38\_ENST00000384176|snoRNA

---

Grc38\_ENST00000384214|snoRNA

---

Grc38\_ENST00000384215|snoRNA

---

Grc38\_ENST00000384220|snoRNA

---

Grc38\_ENST00000384229|snoRNA

---

Grc38\_ENST00000384252|snoRNA

---

Grc38\_ENST00000384262|snoRNA

---

Grc38\_ENST00000384287|snoRNA

---

Grc38\_ENST00000384304|snoRNA

---

Grc38\_ENST00000384320|snoRNA

---

Grc38\_ENST00000384334|snoRNA

---

Grc38\_ENST00000384339|snoRNA

---

Grc38\_ENST00000384342|snoRNA

---

Grc38\_ENST00000384356|snoRNA

---

Grc38\_ENST00000384360|snoRNA

---

Grc38\_ENST00000384365|snoRNA

---

Grc38\_ENST00000384388|snRNA

---

Grc38\_ENST00000384390|snoRNA

---

Grc38\_ENST00000384401|snoRNA

---

Grc38\_ENST00000384416|snoRNA

---

Grc38\_ENST00000384437|snoRNA

---

Grc38\_ENST00000384452|snoRNA

---

Grc38\_ENST00000384512|snoRNA

---

Grc38\_ENST00000384574|snoRNA

---

Grc38\_ENST00000384581|snoRNA

---

Grc38\_ENST00000384606|snRNA

---

Grc38\_ENST00000384662|snoRNA

---

Grc38\_ENST00000384693|snoRNA

---

Grc38\_ENST00000384706|snoRNA

---

Grc38\_ENST00000384714|snoRNA

---

Grc38\_ENST00000384723|snRNA

---

Grc38\_ENST00000384756|snoRNA

---

Grc38\_ENST00000384765|snoRNA

---

Grc38\_ENST00000384769|snoRNA

---

Grc38\_ENST00000384792|snoRNA

---

Grc38\_ENST00000384816|miRNA

---

Grc38\_ENST00000384831|miRNA

---

Grc38\_ENST00000384832|miRNA

---

Grc38\_ENST00000384849|miRNA

---

Grc38\_ENST00000384850|miRNA

---

Grc38\_ENST00000384852|miRNA

---

Grc38\_ENST00000384865|miRNA

---

Grc38\_ENST00000384876|miRNA

---

Grc38\_ENST00000384885|miRNA

---

Grc38\_ENST00000384892|miRNA

---

Grc38\_ENST00000384907|miRNA

---

Grc38\_ENST00000384967|miRNA

---

Grc38\_ENST00000384976|miRNA

---

Grc38\_ENST00000384988|miRNA

---

Grc38\_ENST00000384993|miRNA

---

Grc38\_ENST00000384999|miRNA

---

Grc38\_ENST00000385006|miRNA

---

Grc38\_ENST00000385010|miRNA

---

Grc38\_ENST00000385012|miRNA

---

Grc38\_ENST00000385016|miRNA

---

Grc38\_ENST00000385019|miRNA

---

Grc38\_ENST00000385020|miRNA

---

Grc38\_ENST00000385021|miRNA

---

Grc38\_ENST00000385022|miRNA

---

Grc38\_ENST00000385024|miRNA

---

Grc38\_ENST00000385028|miRNA

---

Grc38\_ENST00000385029|miRNA

---

Grc38\_ENST00000385045|miRNA

---

Grc38\_ENST00000385051|miRNA

---

Grc38\_ENST00000385054|miRNA

---

Grc38\_ENST00000385073|miRNA

---

Grc38\_ENST00000385092|miRNA

---

Grc38\_ENST00000385128|miRNA

---

Grc38\_ENST00000385129|miRNA

---

Grc38\_ENST00000385130|miRNA

---

Grc38\_ENST00000385135|miRNA

---

Grc38\_ENST00000385207|miRNA

---

Grc38\_ENST00000385212|miRNA

---

Grc38\_ENST00000385214|miRNA

---

Grc38\_ENST00000385227|miRNA

---

Grc38\_ENST00000385233|miRNA

---

Grc38\_ENST00000385235|miRNA

---

Grc38\_ENST00000385236|miRNA

---

Grc38\_ENST00000385243|miRNA

---

Grc38\_ENST00000385245|miRNA

---

Grc38\_ENST00000385254|miRNA

---

Grc38\_ENST00000385266|miRNA

---

Grc38\_ENST00000385270|miRNA

---

Grc38\_ENST00000385271|miRNA

---

Grc38\_ENST00000385273|miRNA

---

Grc38\_ENST00000385274|miRNA

---

Grc38\_ENST00000385277|miRNA

---

Grc38\_ENST00000385280|miRNA

---

Grc38\_ENST00000385282|miRNA

---

Grc38\_ENST00000385288|miRNA

---

Grc38\_ENST00000385289|miRNA

---

Grc38\_ENST00000385300|miRNA

---

Grc38\_ENST00000385301|miRNA

---

Grc38\_ENST00000386037|snoRNA

---

Grc38\_ENST00000386062|snoRNA

---

Grc38\_ENST00000386157|snoRNA

---

Grc38\_ENST00000386307|snoRNA

---

Grc38\_ENST00000386683|snoRNA

---

Grc38\_ENST00000386745|snoRNA

---

Grc38\_ENST00000386747|snoRNA

---

Grc38\_ENST00000386847|snoRNA

---

Grc38\_ENST00000386967|snoRNA

---

Grc38\_ENST00000386972|miRNA

---

Grc38\_ENST00000390183|miRNA

---

Grc38\_ENST00000390204|miRNA

---

Grc38\_ENST00000390225|miRNA

---

Grc38\_ENST00000390227|miRNA

---

Grc38\_ENST00000390708|miRNA

---

Grc38\_ENST00000390738|miRNA

---

Grc38\_ENST00000390842|snoRNA

---

Grc38\_ENST00000390856|snoRNA

---

Grc38\_ENST00000390861|snoRNA

---

Grc38\_ENST00000390930|snoRNA

---

Grc38\_ENST00000390981|snoRNA

---

Grc38\_ENST00000391002|snoRNA

---

Grc38\_ENST00000391007|snoRNA

---

Grc38\_ENST00000391076|snoRNA

---

Grc38\_ENST00000391079|snoRNA

---

Grc38\_ENST00000391100|snoRNA

---

Grc38\_ENST00000391141|snoRNA

---

Grc38\_ENST00000391145|snoRNA

---

Grc38\_ENST00000391150|snoRNA

---

Grc38\_ENST00000391162|snoRNA

---

Grc38\_ENST00000391208|snRNA

---

Grc38\_ENST00000391232|snoRNA

---

Grc38\_ENST00000391286|snoRNA

---

Grc38\_ENST00000400436|processed\_transcript

---

Grc38\_ENST00000401190|miRNA

---

Grc38\_ENST00000408061|snoRNA

---

Grc38\_ENST00000408136|miRNA

---

Grc38\_ENST00000408189|snoRNA

---

Grc38\_ENST00000408314|snoRNA

---

Grc38\_ENST00000408373|snoRNA

---

Grc38\_ENST00000408376|snoRNA

---

Grc38\_ENST00000408564|snoRNA

---

Grc38\_ENST00000408573|snoRNA

---

Grc38\_ENST00000408587|snoRNA

---

Grc38\_ENST00000408612|snoRNA

---

Grc38\_ENST00000408749|snRNA

---

Grc38\_ENST00000408813|snoRNA

---

Grc38\_ENST00000408827|miRNA

---

Grc38\_ENST00000410144|snRNA

---

Grc38\_ENST00000410361|snRNA

---

Grc38\_ENST00000410396|snRNA

---

Grc38\_ENST00000410413|snoRNA

---

Grc38\_ENST00000410433|snoRNA

---

Grc38\_ENST00000410457|snRNA

---

Grc38\_ENST00000410482|snRNA

---

Grc38\_ENST00000410545|snRNA

---

Grc38\_ENST00000410712|snRNA

---

Grc38\_ENST00000410818|snRNA

---

Grc38\_ENST00000410991|snRNA

---

Grc38\_ENST00000411053|snRNA

---

Grc38\_ENST00000411292|snoRNA

---

Grc38\_ENST00000411315|snRNA

---

Grc38\_ENST00000411404|snRNA

---

Grc38\_ENST00000412059|processed\_transcript

---

Grc38\_ENST00000413522|snoRNA

---

Grc38\_ENST00000416952|processed\_transcript

---

Grc38\_ENST00000421068|processed\_transcript

---

Grc38\_ENST00000421202|processed\_transcript

---

Grc38\_ENST00000422183|processed\_transcript

---

Grc38\_ENST00000422207|processed\_transcript

---

Grc38\_ENST00000427501|processed\_transcript

---

Grc38\_ENST00000428514|snoRNA

---

Grc38\_ENST00000430247|lincRNA

---

Grc38\_ENST00000430728|lincRNA

---

Grc38\_ENST00000431043|processed\_transcript

---

Grc38\_ENST00000431268|processed\_transcript

---

Grc38\_ENST00000433310|lincRNA

---

Grc38\_ENST00000434796|processed\_transcript

---

Grc38\_ENST00000436656|processed\_transcript

---

Grc38\_ENST00000437681|sense\_intronic

---

Grc38\_ENST00000439232|snoRNA

---

Grc38\_ENST00000442067|processed\_transcript

---

Grc38\_ENST00000443799|processed\_transcript

---

Grc38\_ENST00000444470|processed\_transcript

---

Grc38\_ENST00000445646|lincRNA

---

Grc38\_ENST00000448188|snoRNA

---

Grc38\_ENST00000449589|processed\_transcript

---

Grc38\_ENST00000451607|processed\_transcript

---

Grc38\_ENST00000453784|lincRNA

---

Grc38\_ENST00000454068|processed\_transcript

---

Grc38\_ENST00000454813|processed\_transcript

---

Grc38\_ENST00000456293|processed\_transcript

---

Grc38\_ENST00000456812|processed\_transcript

---

Grc38\_ENST00000458770|snoRNA

---

Grc38\_ENST00000458797|scaRNA

---

Grc38\_ENST00000458838|snoRNA

---

Grc38\_ENST00000458892|snoRNA

---

Grc38\_ENST00000458893|snoRNA

---

Grc38\_ENST00000458974|snoRNA

---

Grc38\_ENST00000459083|snoRNA

---

Grc38\_ENST00000459124|snoRNA

---

Grc38\_ENST00000459155|snoRNA

---

Grc38\_ENST00000459159|snoRNA

---

Grc38\_ENST00000459163|snoRNA

---

Grc38\_ENST00000459174|snoRNA

---

Grc38\_ENST00000459187|snoRNA

---

Grc38\_ENST00000459299|snoRNA

---

Grc38\_ENST00000459342|snoRNA

---

Grc38\_ENST00000459386|snoRNA

---

Grc38\_ENST00000459433|snoRNA

---

Grc38\_ENST00000459473|snoRNA

---

Grc38\_ENST00000459475|snoRNA

---

Grc38\_ENST00000459579|snoRNA

---

Grc38\_ENST00000459584|snoRNA

---

Grc38\_ENST00000459623|snoRNA

---

Grc38\_ENST00000460249|processed\_transcript

---

Grc38\_ENST00000480811|processed\_transcript

---

Grc38\_ENST00000491009|processed\_transcript

---

Grc38\_ENST00000492250|processed\_transcript

---

Grc38\_ENST00000497774|processed\_transcript

---

Grc38\_ENST00000501122|lincRNA

---

Grc38\_ENST00000503991|scaRNA

---

Grc38\_ENST00000505089|lincRNA

---

Grc38\_ENST00000505219|snoRNA

---

Grc38\_ENST00000508832|lincRNA

---

Grc38\_ENST00000510505|lincRNA

---

Grc38\_ENST00000515909|snRNA

---

Grc38\_ENST00000516060|scaRNA

---

Grc38\_ENST00000516089|scaRNA

---

Grc38\_ENST00000516146|snRNA

---

Grc38\_ENST00000516209|snRNA

---

Grc38\_ENST00000516327|snoRNA

---

Grc38\_ENST00000516336|snRNA

---

Grc38\_ENST00000516438|snRNA

---

Grc38\_ENST00000516528|snoRNA

---

Grc38\_ENST00000516564|snRNA

---

Grc38\_ENST00000516672|scaRNA

---

Grc38\_ENST00000516768|scaRNA

---

Grc38\_ENST00000516881|scaRNA

---

Grc38\_ENST00000517038|snRNA

---

Grc38\_ENST00000517041|snRNA

---

Grc38\_ENST00000517097|scaRNA

---

Grc38\_ENST00000517138|scaRNA

---

Grc38\_ENST00000517242|snoRNA

---

Grc38\_ENST00000517277|snRNA

---

Grc38\_ENST00000517961|lincRNA

---

Grc38\_ENST00000521127|processed\_transcript

---

Grc38\_ENST00000534336|lincRNA

---

Grc38\_ENST00000535076|processed\_transcript

---

Grc38\_ENST00000537024|processed\_transcript

---

Grc38\_ENST00000537925|processed\_transcript

---

Grc38\_ENST00000537965|processed\_transcript

---

Grc38\_ENST00000538654|processed\_transcript

---

Grc38\_ENST00000540725|processed\_transcript

---

Grc38\_ENST00000540865|processed\_transcript

---

Grc38\_ENST00000540904|processed\_transcript

---

Grc38\_ENST00000541416|processed\_transcript

---

Grc38\_ENST00000541782|sense\_intronic

---

Grc38\_ENST00000544550|processed\_transcript

---

Grc38\_ENST00000544868|lincRNA

---

Grc38\_ENST00000544983|processed\_transcript

---

Grc38\_ENST00000545308|processed\_transcript

---

Grc38\_ENST00000545688|processed\_transcript

---

Grc38\_ENST00000545920|processed\_transcript

---

Grc38\_ENST00000549804|processed\_transcript

---

Grc38\_ENST00000551361|processed\_transcript

---

Grc38\_ENST00000553465|lincRNA

---

Grc38\_ENST00000554693|lincRNA

---

Grc38\_ENST00000555004|lincRNA

---

Grc38\_ENST00000561320|lincRNA

---

Grc38\_ENST00000561622|3prime\_overlapping\_ncrna

---

Grc38\_ENST00000562952|sense\_overlapping

---

Grc38\_ENST00000567488|lincRNA

---

Grc38\_ENST00000567527|lincRNA

---

Grc38\_ENST00000571722|snoRNA

---

Grc38\_ENST00000573259|processed\_transcript

---

Grc38\_ENST00000573457|processed\_transcript

---

Grc38\_ENST00000574616|3prime\_overlapping\_ncrna

---

Grc38\_ENST00000574846|processed\_transcript

---

Grc38\_ENST00000575226|processed\_transcript

---

Grc38\_ENST00000575766|processed\_transcript

---

Grc38\_ENST00000576096|processed\_transcript

---

Grc38\_ENST00000576410|processed\_transcript

---

Grc38\_ENST00000576702|processed\_transcript

---

Grc38\_ENST00000577122|processed\_transcript

---

Grc38\_ENST00000577700|lincRNA

---

Grc38\_ENST00000577887|snoRNA

---

Grc38\_ENST00000577988|snoRNA

---

Grc38\_ENST00000578757|processed\_transcript

---

Grc38\_ENST00000579017|miRNA

---

Grc38\_ENST00000579879|snoRNA

---

Grc38\_ENST00000580533|snoRNA

---

Grc38\_ENST00000580972|snRNA

---

Grc38\_ENST00000581525|snoRNA

---

Grc38\_ENST00000582661|miRNA

---

Grc38\_ENST00000582890|miRNA

---

Grc38\_ENST00000582965|lincRNA

---

Grc38\_ENST00000583032|snoRNA

---

Grc38\_ENST00000583619|snoRNA

---

Grc38\_ENST00000583861|snoRNA

---

Grc38\_ENST00000584275|snoRNA

---

Grc38\_ENST00000584923|snoRNA

---

Grc38\_ENST00000584949|snoRNA

---

Grc38\_ENST00000586185|lincRNA

---

Grc38\_ENST00000586231|lincRNA

---

Grc38\_ENST00000589496|3prime\_overlapping\_ncrna

---

Grc38\_ENST00000591384|lincRNA

---

Grc38\_ENST00000591554|miRNA

---

Grc38\_ENST00000602361|lincRNA

---

Grc38\_ENST00000602478|lincRNA

---

Grc38\_ENST00000602573|lincRNA

---

Grc38\_ENST00000602755|lincRNA

---

Grc38\_ENST00000604135|sense\_intronic

---

Grc38\_ENST00000605502|lincRNA

---

Grc38\_ENST00000605533|sense\_intronic

---

Grc38\_ENST00000606190|snRNA

---

Grc38\_ENST00000606349|miRNA

---

Grc38\_ENST00000606412|snoRNA

---

Grc38\_ENST00000606577|snoRNA

---

Grc38\_ENST00000606623|snRNA

---

Grc38\_ENST00000606769|snoRNA

---

Grc38\_ENST00000607313|snoRNA

---

Grc38\_ENST00000607520|processed\_transcript

---

Grc38\_ENST00000607707|snoRNA

---

Grc38\_ENST00000608412|lincRNA

---

Grc38\_ENST00000609276|lincRNA

---

Grc38\_ENST00000610851|lincRNA

---

Grc38\_ENST00000611296|lincRNA

---

Grc38\_ENST00000612496|lincRNA

---

Grc38\_ENST00000613527|miRNA

---

Grc38\_ENST00000613917|lincRNA

---

Grc38\_ENST00000613956|snRNA

---

Grc38\_ENST00000614083|snRNA

---

Grc38\_ENST00000615356|snRNA

---

Grc38\_ENST00000616315|lincRNA

---

Grc38\_ENST00000616527|lincRNA

---

Grc38\_ENST00000616691|lincRNA

---

Grc38\_ENST00000617320|miRNA

---

Grc38\_ENST00000618132|lincRNA

---

Grc38\_ENST00000618227|lincRNA

---

Grc38\_ENST00000618589|lincRNA

---

Grc38\_ENST00000618925|lincRNA

---

Grc38\_ENST00000618978|snRNA

---

Grc38\_ENST00000619178|snoRNA

---

Grc38\_ENST00000619225|snRNA

---

Grc38\_ENST00000619449|lincRNA

---

Grc38\_ENST00000620232|snoRNA

---

Grc38\_ENST00000620268|snRNA

---

Grc38\_ENST00000620446|snoRNA

---

Grc38\_ENST00000620465|lincRNA

---

Grc38\_ENST00000621466|miRNA

---

Grc38\_ENST00000621667|miRNA

---

Grc38\_ENST00000621753|snRNA

---

Grc38\_ENST00000622286|snRNA

---

Grc38\_ENST00000622328|lincRNA

---

Grc38\_ENST00000623391|lincRNA

---

Grc38\_ENST00000625314|snoRNA

---

Grc38\_ENST00000625845|snoRNA

---

Grc38\_ENST00000625876|snoRNA

---

Grc38\_ENST00000625943|snoRNA

---

Grc38\_ENST00000626826|macro\_lncRNA

---

Grc38\_ENST00000626830|miRNA

---

Grc38\_ENST00000626886|snoRNA

---

Grc38\_ENST00000626963|snoRNA

---

Grc38\_ENST00000627324|scaRNA

---

Grc38\_ENST00000627983|snoRNA

---

Grc38\_ENST00000628177|snoRNA

---

Grc38\_ENST00000628458|snoRNA

---

Grc38\_ENST00000628672|miRNA

---

Grc38\_ENST00000628908|miRNA

---

Grc38\_ENST00000629038|snoRNA

---

Grc38\_ENST00000629045|scaRNA

---

Grc38\_ENST00000629167|miRNA

---

Grc38\_ENST00000629259|miRNA

---

Grc38\_ENST00000629295|lincRNA

---

Grc38\_ENST00000629478|snRNA

---

Grc38\_ENST00000629536|snoRNA

---

Grc38\_ENST00000629629|snoRNA

---

Grc38\_ENST00000629784|miRNA

---

Grc38\_ENST00000630092|snoRNA

---

Grc38\_ENST00000630110|miRNA

---

Grc38\_ENST00000630429|snoRNA

---

Grc38\_ENST00000630949|snoRNA

---

Grc38\_ENST00000631292|snoRNA

---

lncrnadb\_7sk\_homosapiens\_1|Homo

---

lncrnadb\_kcnq1ot1\_homosapiens\_1|Homo

---

lncrnadb\_neat1\_homosapiens\_2|Homo

---

mirBASE\_efT-miR-26c

---

mirBASE\_tch-miR-27a-3p

---

piRNA|DQ569993

---

piRNA|DQ571511

---

piRNA|DQ582536

---

piRNA|DQ587514

---

piRNA|DQ598646

---

snoRNADB\_E3

---

snoRNADB\_HBII-135

---

snoRNADB\_HBII-202

---

snoRNADB\_U104

---

snoRNADB\_U17b

**Elements in Over expressed in CAF-EXO, Over expressed in NF-CELL and Over expresseed in CAF-CELL**

**Elements in Over expressed in CAF-EXO, Over expressed in NF-CELL, Over expressed in CAF-CELL and Over expressed in NF-EXO**

**Elements in Over expressed in CAF-EXO and Over expressed in NF-EXO**
  


---

Grc38\_ENST00000360737|lincRNA

---

Grc38\_ENST00000362291|miRNA

---

Grc38\_ENST00000363306|snRNA

---

Grc38\_ENST00000363334|snRNA

---

Grc38\_ENST00000363426|snRNA

---

Grc38\_ENST00000364300|snRNA

---

Grc38\_ENST00000364421|snRNA

---

Grc38\_ENST00000365477|snRNA

---

Grc38\_ENST00000383858|snRNA

---

Grc38\_ENST00000383975|snRNA

---

Grc38\_ENST00000384093|snRNA

---

Grc38\_ENST00000384425|snRNA

---

Grc38\_ENST00000384446|snRNA

---

Grc38\_ENST00000384499|snRNA

---

Grc38\_ENST00000384550|snoRNA

---

Grc38\_ENST00000384592|snRNA

---

Grc38\_ENST00000385044|miRNA

---

Grc38\_ENST00000385302|miRNA

---

Grc38\_ENST00000387069|snRNA

---

Grc38\_ENST00000408240|miRNA

---

Grc38\_ENST00000410794|snRNA

---

Grc38\_ENST00000415386|lincRNA

---

Grc38\_ENST00000423943|lincRNA

---

Grc38\_ENST00000427111|lincRNA

---

Grc38\_ENST00000443364|lincRNA

---

Grc38\_ENST00000445817|lincRNA

---

Grc38\_ENST00000456105|lincRNA

---

Grc38\_ENST00000459170|miRNA

---

Grc38\_ENST00000459274|snRNA

---

Grc38\_ENST00000501143|lincRNA

---

Grc38\_ENST00000511821|lincRNA

---

Grc38\_ENST00000527474|lincRNA

---

Grc38\_ENST00000559030|lincRNA

---

Grc38\_ENST00000563151|lincRNA

---

Grc38\_ENST00000565467|lincRNA

---

Grc38\_ENST00000568394|lincRNA

---

Grc38\_ENST00000569832|processed\_transcript

---

Grc38\_ENST00000573479|lincRNA

---

Grc38\_ENST00000577388|miRNA

---

Grc38\_ENST00000578924|miRNA

---

Grc38\_ENST00000579933|lincRNA

---

Grc38\_ENST00000580233|miRNA

---

Grc38\_ENST00000580344|miRNA

---

Grc38\_ENST00000581316|miRNA

---

Grc38\_ENST00000581364|miRNA

---

Grc38\_ENST00000582082|miRNA

---

Grc38\_ENST00000582090|miRNA

---

Grc38\_ENST00000582216|miRNA

---

Grc38\_ENST00000583357|miRNA

---

Grc38\_ENST00000584072|miRNA

---

Grc38\_ENST00000584178|miRNA

---

Grc38\_ENST00000584443|miRNA

---

Grc38\_ENST00000593917|processed\_transcript

---

Grc38\_ENST00000602301|vaultRNA

---

Grc38\_ENST00000604849|lincRNA

---

Grc38\_ENST00000609880|lincRNA

---

Grc38\_ENST00000610122|lincRNA

---

Grc38\_ENST00000610524|miRNA

---

Grc38\_ENST00000611066|miRNA

---

Grc38\_ENST00000611300|sRNA

---

Grc38\_ENST00000612171|miRNA

---

Grc38\_ENST00000612766|miRNA

---

Grc38\_ENST00000613558|miRNA

---

Grc38\_ENST00000617238|miRNA

---

Grc38\_ENST00000617702|lincRNA

---

Grc38\_ENST00000617883|miRNA

---

Grc38\_ENST00000619973|miRNA

---

Grc38\_ENST00000621427|miRNA

---

Grc38\_ENST00000621981|miRNA

---

Grc38\_ENST00000623395|lincRNA

---

Grc38\_ENST00000624919|sense\_overlapping

---

Grc38\_ENST00000625614|miRNA

---

Grc38\_ENST00000625656|miRNA

---

Grc38\_ENST00000626018|miRNA

---

Grc38\_ENST00000626279|miRNA

---

Grc38\_ENST00000626443|miRNA

---

Grc38\_ENST00000626841|miRNA

---

Grc38\_ENST00000626896|miRNA

---

Grc38\_ENST00000627736|miRNA

---

Grc38\_ENST00000627889|miRNA

---

Grc38\_ENST00000628052|miRNA

---

Grc38\_ENST00000628701|miRNA

---

Grc38\_ENST00000630014|miRNA

---

Grc38\_ENST00000630551|miRNA

---

Grc38\_ENST00000630620|miRNA

---

Grc38\_ENST00000630786|miRNA

---

Grc38\_ENST00000630788|miRNA

---

lncrnadb\_7sl\_homosapiens\_1|Homo

---

lncrnadb\_mt-lipcar\_hg\_1|Homo

---

lncrnadb\_Y-RNAs\_hg\_1|Homo

---

lncrnadb\_yam-1\_mm10\_1|Mus

---

mirBASE\_bta-miR-2889

---

mirBASE\_hsa-miR-4485-3p

---

piRNA|DQ570940

---

piRNA|DQ570956

---

piRNA|DQ570992

---

piRNA|DQ571031

---

piRNA|DQ571524

---

piRNA|DQ571813

---

piRNA|DQ575656

---

piRNA|DQ575658

---

piRNA|DQ575660

---

piRNA|DQ575882

---

piRNA|DQ575884

---

piRNA|DQ576605

---

piRNA|DQ576872

---

piRNA|DQ576918

---

piRNA|DQ577772

---

piRNA|DQ582264

---

piRNA|DQ582838

---

piRNA|DQ584698

---

piRNA|DQ588594

---

piRNA|DQ590013

---

piRNA|DQ590548

---

piRNA|DQ590835

---

piRNA|DQ592931

---

piRNA|DQ592932

---

piRNA|DQ592953

---

piRNA|DQ593325

---

piRNA|DQ593356

---

piRNA|DQ593358

---

piRNA|DQ593407

---

piRNA|DQ593423

---

piRNA|DQ594453

---

piRNA|DQ594465

---

piRNA|DQ595536

---

piRNA|DQ596538

---

piRNA|DQ596805

---

piRNA|DQ597110

---

piRNA|DQ597215

---

piRNA|DQ597217

---

piRNA|DQ597218

---

piRNA|DQ597341

---

piRNA|DQ597347

---

piRNA|DQ597397

---

piRNA|DQ597403

---

piRNA|DQ597482

---

piRNA|DQ597916

---

piRNA|DQ597971

---

piRNA|DQ597975

---

piRNA|DQ598008

---

piRNA|DQ598167

---

piRNA|DQ598180

---

piRNA|DQ598183

---

piRNA|DQ598252

---

piRNA|DQ598312

---

piRNA|DQ598445

---

piRNA|DQ598639

---

piRNA|DQ598675

---

piRNA|DQ598677

---

piRNA|DQ600952

---

Y\_RNA\_AADD01087475.1/2469-2552

---

Y\_RNA\_AADN03001926.1/468172-468061

---

Y\_RNA\_AAFC03099237.1/8865-8959

---

Y\_RNA\_AAFC03099238.1/121707-121818

---

Y\_RNA\_AAFR03014070.1/16387-16488

---

Y\_RNA\_AAHX01030026.1/7139-7028

---

Y\_RNA\_AAPE02039776.1/102994-103096

---

Y\_RNA\_AAPN01172659.1/53662-53775

---

Y\_RNA\_AAPY01489510.1/220-119

---

Y\_RNA\_AAQR03087348.1/2666-2565

---

Y\_RNA\_AAQR03087350.1/1071-976

---

Y\_RNA\_AAWR02036109.1/33571-33459

---

Y\_RNA\_AAYZ01133134.1/5808-5901

---

Y\_RNA\_ABDC01601884.1/580-486

---

Y\_RNA\_AC073140.5/117955-118049

---

Y\_RNA\_AC190269.3/73147-73248

---

Y\_RNA\_AC191785.1/102750-102844

---

Y\_RNA\_AC193857.1/159000-159109

---

Y\_RNA\_AC205306.6/147779-147685

---

Y\_RNA\_CT104657.1/45296-45390

---

Y\_RNA\_CT104669.1/59726-59616

**Elements in Over expressed in NF-CELL and Over expressed in NF-EXO**

**Elements in Over expressed in CAF-CELLand Over expressed in NF-EXO**

**Elements in Over expressed in NF-CELL, Over expressed in CAF-CELLand Over expressed in NF-EXO**

**Elements in Over expressed in CAF-EXO, Over expressed in NF-CELL and Over expressed in NF-EXO**

**Elements in Over expressed in CAF-EXO, Over expressed in CAF-CELLand Over expressed in NF-EXO**

Over expressed in CAF-EXO

Over expressed in NF-CELL

Over expresseed in CAF-CELL

Over expressed in NF-EXO
