# Supplementary material for: Differential distribution and enrichment of non-coding RNAs in exosomes from normal and Cancer-associated fibroblasts in colorectal cancer
Source: Mol Cancer. 2018 Aug 3;17:114. doi: 10.1186/s12943-018-0863-4 (PMC6091058; doi:10.1186/s12943-018-0863-4)
Supplement: Supplementary file 11 — Table S1. sncRNAs distributed differently in CAF-EXO samples from in NF-EXO ones. Highly significant lncRNAs and sncRNAs (FDR < 1E-04) are highlighted in bold. (DOCX 79 kb) [file 12943_2018_863_MOESM11_ESM.docx]

| Table 1.- sncRNAs differentially distributed in TEXO samples respect to NEXO | | |
| --- | --- | --- |
| Down-distributed sncRNAs | | |
| Species | | FDR |
| miRNA\|hsa-mir-335 | | 0.0423 |
| miRNA\|hsa-mir-379 | | 0.0325 |
| miRNA\|hsa-mir-126 | | 0.0093 |
| miRNA\|hsa-mir-224 | | 0.0228 |
| miRNA\|hsa-mir-146b | | 0.0425 |
| miRNA\|hsa-mir-26b | | 0.0251 |
| miRNA\|hsa-mir-98 | | 0.0499 |
| miRNA\|hsa-mir-487a | | 0.0358 |
| Over-distributed in lncRNAs | | |
| Family 1 | Species | FDR |
|  | snRNA\|RNU6-88P | 0.0188 |
|  | snRNA\|RNU6-373P | 0.0278 |
|  |  |  |
| Family 2 | piRNA\|piR-36034 | 0.0083 |
|  | piRNA\|piR-36037 | **0.0003** |
|  | piRNA\|piR-36249 | **8.70E-07** |
| Family 3 | miRNA\|AL353644.1 | 0.0423 |
|  | miRNA\|hsa-mir-6087 | 0.0048 |
| Family 4 | snRNA\|RNU1-11P | **1.95E-05** |
|  | snRNA\|RNU1-13P | **9.70E-06** |
|  | snRNA\|RNU1-16P | 0.0083 |
|  | snRNA\|NVU1-17 | **9.87E-08** |
|  | snRNA\|RNU1-20P | 0.0010 |
|  | snRNA\|RNU1-22P | 0.0010 |
|  | snRNA\|RNU1-34P | 0.0038 |
|  | snRNA\|RNU1-46P | **0.0002** |
|  | snRNA\|RNU1-67P | 0.0278 |
|  | snRNA\|RNU1-85P  snRNA\|RNU1-89P | 0.0352  **0.0002** |
|  | snRNA\|RNU1-103P | 0.0123 |
|  | snRNA\|RNU1-132P | 0.0299 |
|  | snRNA\|RNU1-148P | 0.0083 |
|  | snRNA\|RNVU1-1 | **9.87E-08** |
|  | snRNA\|RNVU1-7 | **3.57E-05** |
|  | snRNA\|RNVU1-15 | **9.87E-08** |
|  | snRNA\|RNVU1-19 | **9.87E-08** |
|  | snRNA\|U1 | 0.0101 |
|  | snRNA\|U1 | 0.0263 |
|  | snRNA\|U1 | **7.51E-07** |
|  | snRNA\|U1 | **9.87E-08** |
|  | snRNA\|U1 | **9.87E-08** |
|  |  |  |
| No Family |  |  |
|  | miRNA\|MIR2889 | 0.0309 |
|  | miRNA\|MIR4449 | 0.0083 |
|  | miRNA\|hsa-mir-4532 | 0.0194 |
|  | miRNA\|hsa-mir-4508 | 0.0278 |
|  | miRNA\|AC007216.1 | 0.0188 |
|  | miRNA\|AL390776.1 | 0.0492 |
|  | miRNA\|AC010724.1-201 | 0.0281 |
|  | snRNA\|RNU11 | **0.0001** |
|  | piRNA\|piR-31143 | 0.0482 |
|  | piRNA\|piR-37544 | 0.0195 |
|  | piRNA\|piR-40881 | 0.0011 |
|  | piRNA\|piR-57251 | **3.27E-08** |
|  |  |  |
| Names are extracted from Biomart Ensembl excepting piRNAs,which are named using to GenBank accessions. False Discovery Rates (FDRs) < 1E-04 are higlighted bold. For more details see Supplementary file 12. | | |
