# Supplementary material for: Differential distribution and enrichment of non-coding RNAs in exosomes from normal and Cancer-associated fibroblasts in colorectal cancer
Source: Mol Cancer. 2018 Aug 3;17:114. doi: 10.1186/s12943-018-0863-4 (PMC6091058; doi:10.1186/s12943-018-0863-4)
Supplement: Supplementary file 14 — : Table S2. Target genes for CAF-EXO over-distributed sncRNAs supported by FDR < 1E-04. (DOCX 14 kb) [file 12943_2018_863_MOESM14_ESM.docx]

| Supplementary Table 2.- Target genes for CAF-EXO over-distributed sncRNAs supported by FDR < 1E-04 | | | | |
| --- | --- | --- | --- | --- |
| sncRNAs | Genes | Genes Names | GOs | Paths |
| Family 2 | 65 | EPDR1 CSF1 RAD54L DPH1 PTK7 RHBDF2 RAD51D HIP1 HRAS TPD52 WNK2 TPO SMAD2 RASSF1 C2orf40 FH MDM2 MSH6 CSF1R ERBB2 XRCC3 PTPRK TCF7L2 RSPO2 DAB2 CASC15 ST13 ESR1 MAP2K4 PALLD EP300 BRCA1 CTTN BAX TSG101 NAMPT QKI CSF3 BCAR3 KLF6 CD82 CUX1 EIF4G1 STARD10 SRC CHRNA3 AKT1 AXIN2 FBXW7 NFXL1 MUTYH ATM HMMR HULC PALB2 RNF43 MAX NEMF POLD1 EPCAM FGFR4 NCOA3 MSH2 ATR VGLL3 | 57 | 22 |
| Family 4 | 15 | TCF7L2 TSG101 CD82 CHRNA3 AXIN2 MUTYH HMMR RNF43 VGLL3 TBL1XR1 VGLL3 TBL1XR1 CSF1 RAD54L RASSF1 CSF1R MSH6 CASC15 | 24 | 12 |
| RNU11 | 7 | RASSF1 MSH6 MUTYH POLD1 MSH2 TCF7L2 AXIN2 | 6 | 4 |
| piR-57251 | 62 | EPDR1 CSF1 RAD54L DPH1 PTK7 RHBDF2 RAD51D HIP1 HRAS TPD52 TPO SMAD2 RASSF1 C2orf40 FH MDM2 MSH6 CSF1R ERBB2 XRCC3 PTPRK TCF7L2 RSPO2 DAB2 CASC15 ST13 ESR1 MAP2K4 PALLD EP300 BRCA1 CTTN BAX TSG101 NAMPT QKI CSF3 BCAR3 KLF6 CD82 CUX1 EIF4G1 STARD10 SRC CHRNA3 AKT1 AXIN2 FBXW7 NFXL1 MUTYH ATM PALB2 RNF43 MAX NEMF POLD1 EPCAM FGFR4 NCOA3 MSH2 ATR VGLL3 | 55 | 22 |
| More details about target gene predictions are provided in Supplementary material 10. Number of GO and metabolic pathway annotations of all predicted target genes are summarized in the table. | | | | |
